# Supplementary material for: Long-term effects of bariatric surgery on acute kidney injury: a propensity-matched cohort in the UK Clinical Practice Research Datalink
Source: BMJ Open. 2018 May 16;8(5):e020371. doi: 10.1136/bmjopen-2017-020371 (PMC5961582; doi:10.1136/bmjopen-2017-020371)
Supplement: Supplementary data [file bmjopen-2017-020371supp001.pdf]

Long-term effects of bariatric surgery on acute kidney injury: A propensity-matched cohort in the United Kingdom Clinical Practice Research Datalink

Supporting Information

Overview

S1 Appendix – Code List for Identification of patients with bariatric surgery

S2 Appendix – Association of potential confounders with bariatric surgery and AKI

S3 Appendix – Patient selection from the original cohort

S4 Appendix – Sensitivity Analyses

S1 Appendix

Appendix 1: Code List for identification of patients with bariatric surgery from the CPRD database as published by Douglas et al. [7]

Read code	*description*
76132.00	Laparoscopic adjustable gastric banding
76134.00	Partitioning of stomach using staples
76131.11	Mason vertical banded gastroplasty
76133.00	Partitioning of stomach using band
76116.00	Laparoscopic sleeve gastrectomy
76115.00	Sleeve gastrectomy NEC
76425.00	Duodenal switch
76135.00	Partitioning of stomach NEC
76114.00	Sleeve gastrectomy and duodenal switch
76166.00	Laparoscopic gastric bypass

S2 Appendix

Appendix 2: Identification of potential confounders in the association of bariatric surgery (exposure) and the endpoint of incident AKI (outcome) in patients of the linked CPRD/HES cohort

	RR (95%CI)	Change in %	Selection for multivariable model
**Crude effect estimate**	0.62 (0.40, 0.95)		
**Effect estimates when individually adjusting for**			
**Age**	0.62 (0.40, 0.95)	0.2 %	yes (a priori)
**Sex**	0.60 (0.39, 0.92)	2.7 %	yes (a priori)
**Calendar Time**	0.61 (0.40, 0.94)	0.9%	yes (a priori)
**CKD status at baseline**	0.59 (0.38, 0.91)	4.4 %	yes (a priori)
**BMI at baseline**	0.53 (0.34, 0.83)	13.9 %	yes
**Alcohol Status**	0.61 (0.40, 0.93)	1.3 %	no
**Smoking Status**	0.61 (0.40, 0.94)	0.3 %	no
**History of cerebrovascular disease**	0.61 (0.40, 0.94)	0.6 %	no
**History of coronary heart disease**	0.60 (0.39, 0.91)	3.3 %	no
**History of peripheral vascular disease**	0.64 (0.41, 0.98)	3.2 %	no
**History of other atheroma**	0.62 (0.40, 0.95)	0.0 %	no
**History of diabetes**	0.60 (0.39, 0.92)	2.7%	no
**History of taking oral antidiabetics**	0.55 (0.36, 0.85)	10.4%	yes
**History of taking insulin**	0.57 (0.37, 0.87)	7.9 %	no
**History of hypertension**	0.61 (0.40, 0.94)	1.1 %	no
**History of statin use**	0.58 (0.38, 0.89)	5.5 %	no
**History of AKI**	0.42 (0.26, 0.67)	31.9 %	yes

Variables were added individually to the univariable model testing the association between bariatric surgery and AKI. If the addition of the respective variable changed the model ≥10% then the variable was selected to be included in the multivariable model.

AKI = acute kidney injury, BMI = body mass index, CKD = chronic kidney disease

S3 Appendix

Appendix 3: Patient selection from the original cohort as described in Douglas et al [7]

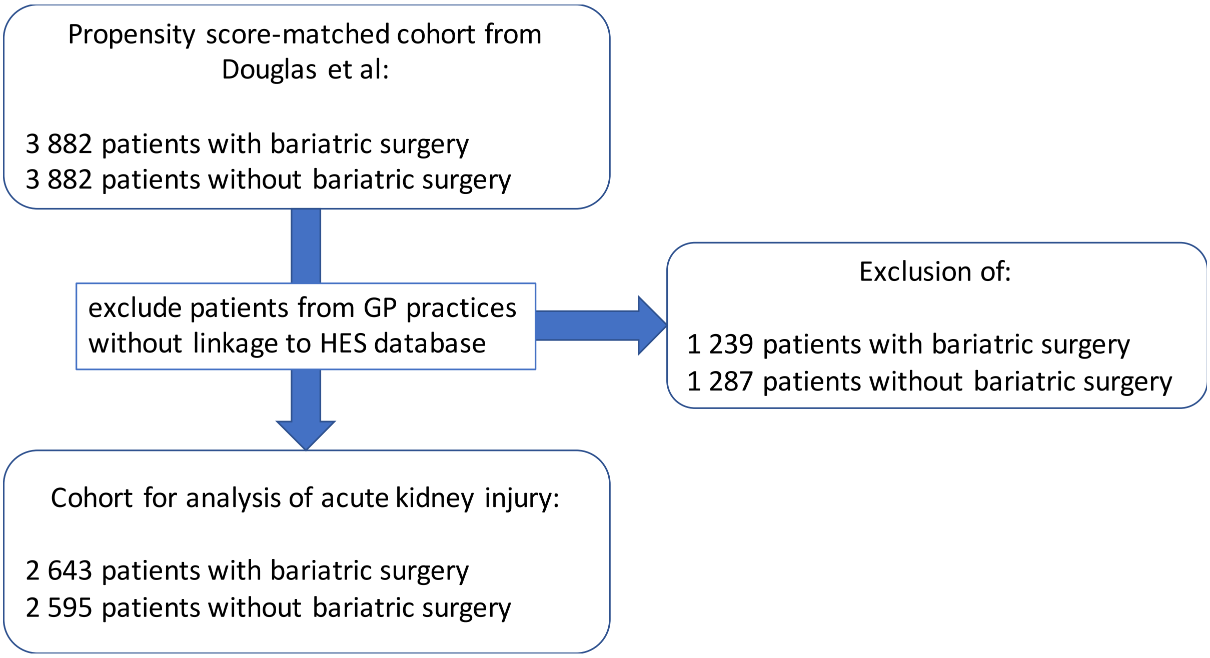

S4 Appendix

Appendix 4: Sensitivity analyses for the association of bariatric surgery with acute kidney injury

	PY	Events	Rate per 1000 PY (95% CI)	Crude RR (95% CI)[1]	p-value[2]	Adjusted RR (95% CI)[3]	p-value[2]
**Restricted to patients without CKD at baseline (available serum creatinine measures + eGFR ≥60)**							
**Day 1-30**							
Unexposed	98	0	0	-			
Bariatric surgery	111	<5[6]	36.2 (13.6, 96.3)	-			
**>Day 30**							
Unexposed	3,550	27	7.6 (5.2, 11.1)	-			
Bariatric surgery	4,311	22	5.1 (3.4, 7.7)	0.67 (0.38, 1.18)	0.165	0.53 (0.29, 1.00)	0.050
**Restricted to patients without known CKD at baseline (available serum creatinine measures + eGFR ≥60 or missing eGFR at baseline)**							
**Day 1-30**							
Unexposed	199	0	0	-			
Bariatric surgery	195	<5[6]	20.5 (7.7, 54.7)	-			
**>Day 30**							
Unexposed	7,735	42	5.4 (4.0, 7.3)	-			
Bariatric surgery	7,930	27	3.4 (2.3, 5.0)	0.63 (0.39, 1.02)	0.058	0.42 (0.25, 0.73)	0.002
**Excluding patients with CKD stage 4**							
**Day 1-30**							
Unexposed	203	0	0	-			
Bariatric surgery	198	5	25.2 (10.5, 60.6)	-			
> **Day 30**							
Unexposed	7,875	52	6.6 (5.0, 8.7)	-			
Bariatric surgery	8,037	32	4.0 (2.8, 5.6)	0.60 (0.39, 0.94)	0.024	0.35 (0.21, 0.59)	<0.001
**Restricted to patients with T2DM**							
**Day 1-30**							
Unexposed	65	0	0	-			
Bariatric surgery	69	<5[6]	43.6 (14.1, 135.1)	-			
**>Day 30**							
Unexposed	2,325	33	14.2 (10.1, 20.0)	-			
Bariatric surgery	2,548	18	7.1 (4.5, 11.2)	0.50 (0.28, 0.88)	0.017	0.25 (0.13, 0.51)	<0.001
**Restricted to patients with a history of taking insulin**							
**Day 1-30**							
Unexposed	11	0	0	-			
Bariatric surgery	13	0	0	-			
**>Day 30**							
Unexposed	321	11	34.3 (19.0, 61.9)	-			
Bariatric surgery	502	9	17.9 (9.3, 34.5)	0.52 (0.22, 1.26)	0.150	0.22 (0.08, 0.64)	0.005
**Restricted to ICD-10 codes N17.0 and N17.9**							
**Day 1-30**							
Unexposed	202	0	0	-			
Bariatric surgery	199	5	25.2 (10.5, 60.5)	-			
**>Day 30**							
Unexposed	7,871	48	6.1 (4.6, 8.1)	-			
Bariatric surgery	8,055	31	3.8 (2.7, 5.5)	0.63 (0.40, 0.99)	0.046	0.40 (0.24, 0.67)	<0.001
**Having an initial post-surgery time span of 60 days instead of 30**							
**Day 1-60**							
Unexposed	403	<5[6]	2.5 (0.3, 17.6)	-			
Bariatric surgery	395	6	15.2 (6.8, 33.8)	6.11 (0.74, 50.8)	0.094	[4]	
> **Day 60**							
Unexposed	7,682	53	6.9 (5.3, 9.0)	-			
Bariatric surgery	7,864	33	4.2 (3.0, 5.9)	0.61 (0.39, 0.94)	0.025	0.38 (0.23, 0.63)	<0.001
				Test for interaction[5]	0.011		

**Including patients with extreme propensity scores**							
**Day 1-30**							
Unexposed	208	0	0	-			
Bariatric surgery	206	5	24.3 (10.1, 58.3)	-			
> **Day 30**							
Unexposed	8,054	59	7.3 (5.7, 9.5)	-			
Bariatric surgery	8,324	34	4.1 (2.9, 5.7)	0.56 (0.37, 0.85)	0.007	0.33 (0.20, 0.54)	<0.001
**Excluding patients with BMI < 35 kg/m² at baseline**							
**Day 1-30**							
Unexposed	180	0	0	-			
Bariatric surgery	175	<5[6]	22.8 (8.6, 60.9)	-			
> **Day 30**							
Unexposed	6,714	48	7.1 (5.4, 9.5)	-			
Bariatric surgery	7,100	32	4.5 (3.2, 6.4)	0.63 (0.40, 0.99)	0.043	0.39 (0.23, 0.65)	<0.001

[1] Poisson regression model
[2] Wald test for RR, Likelihood-Ratio Test for interaction
[3] Poisson regression model adjusted for age at baseline, sex, calendar time, CKD at baseline, history of AKI, history of taking oral antidiabetics, and BMI at baseline
[4] No analysis for day 1-30 owing to sparse data
[5] Test for interaction of the effect estimate with the time periods 1-30 days and >30 days
[6] cell counts <5 have been suppressed to ensure anonymity
AKI = acute kidney injury, CKD = chronic kidney disease, PY = person-years, RR = rate ratio
