# Supplementary material for: Long-term effects of bariatric surgery on acute kidney injury: a propensity-matched cohort in the UK Clinical Practice Research Datalink
Source: BMJ Open. 2018 May 16;8(5):e020371. doi: 10.1136/bmjopen-2017-020371 (PMC5961582; doi:10.1136/bmjopen-2017-020371)
Supplement: Author's manuscript [file bmjopen-2017-020371.draft_revisions.pdf]

BMJ Open is committed to open peer review. As part of this commitment we make the peer review history of every article we publish publicly available.

When an article is published we post the peer reviewers' comments and the authors' responses online. We also post the versions of the paper that were used during peer review. These are the versions that the peer review comments apply to.

The versions of the paper that follow are the versions that were submitted during the peer review process. They are not the versions of record or the final published versions. They should not be cited or distributed as the published version of this manuscript.

BMJ Open is an open access journal and the full, final, typeset and author-corrected version of record of the manuscript is available on our site with no access controls, subscription charges or pay-per-view fees (http://bmjopen.bmj.com).

If you have any questions on BMJ Open's open peer review process please email info.bmjopen@bmj.com

BMJ Open

Long-term effects of bariatric surgery on acute kidney injury: A propensity-matched cohort in the United Kingdom Clinical Practice Research Datalink

Journal:	*BMJ Open*
Manuscript ID	bmjopen-2017-020371
Article Type:	Research
Date Submitted by the Author:	02-Nov-2017

Complete List of Authors:	Koppe, Uwe; Robert Koch Institut,<br>Nitsch, Dorothea; LSHTM<br>Mansfield, Kathryn; London School of Hygiene and Tropical Medicine, Non-communicable Disease Epidemiology<br>Mathur, Rohini; London School of Hygiene and Tropical Medicine, Department of Non-Communicable Disease Epidemiology; Queen Mary University of London, Centre for Primary Care & Public Health<br>Bhaskaran, Krishnan; LSHTM, NCDE<br>Batterham, Rachel; UCL, Centre for Obesity Research, Rayne Institute, Department of Medicine<br>Smeeth, Liam; London School of Hygiene and Tropical Medicine, Epidemiology and Population Health<br>Douglas, Ian; London School of Hygiene and Tropical Medicine, Epidemiology and Population Health

**Primary Subject Heading**:	Epidemiology
Secondary Subject Heading:	Renal medicine
Keywords:	Clinical Practice Research Datalink, Acute Kidney Injury, Obesity, Bariatric Surgery

SCHOLARONE™ Manuscripts

Title Long-term effects of bariatric surgery on acute kidney injury: A propensity-matched cohort in the United Kingdom Clinical Practice Research Datalink

Running headline Bariatric surgery and acute kidney injury

Authors Uwe Koppe[1,2*], Dorothea Nitsch[1], Kathryn E. Mansfield[1], Rohini Mathur[1], Krishnan Bhaskaran[1], Rachel L. Batterham[3,4,5], Liam Smeeth[1], Ian J. Douglas[1]

[1] Faculty of Epidemiology and Population Health, London School of Hygiene & Tropical Medicine, London, United Kingdom,

[2] Department of Infectious Disease Epidemiology, Robert Koch Institute, Berlin, Germany,

[3] Centre for Obesity Research, Rayne Institute, Department of Medicine, University College London, London, United Kingdom,

[4] University College London Hospital Bariatric Centre for Weight Management and Metabolic Surgery, London, United Kingdom,

[5] National Institute of Health Research, University College London Hospital Biomedical Research Centre, London, United Kingdom

* Correspondence to:

Dr. Uwe Koppe
Robert Koch Institut
Seestr. 10
13353 Berlin, Germany
Tel: +49 30 18754 2262
Fax: +49 30 18754 3533
koppeu@rki.de

Word count: 3,358

For peer review only

ABSTRACT

Objective: Bariatric surgery is an effective method of weight reduction and has been associated with acute kidney injury (AKI) as a perioperative event. However, the long-term effects of the weight reduction after surgery on AKI are unknown. The objective of this study is to quantify the association of bariatric surgery with later risk of AKI.

Design: This study uses a propensity-score matched cohort of patients from the United Kingdom Clinical Practice Research Datalink database with and without bariatric surgery to compare rates of AKI episodes derived from linkage to the Hospital Episode Statistics.

Setting: England, United Kingdom

Participants: We included 2,643 patients with bariatric surgery and 2,595 patients without.

Results: Results were compatible with an increased risk of AKI in the first 30 days following surgery compared with patients without surgery, but AKI incidence was substantially decreased in patients with bariatric surgery during long-term follow-up (rate ratio 0.37, 95% CI 0.23, 0.61) even after accounting for chronic kidney disease status at baseline. Over the whole period of follow-up, bariatric surgery had a net protective effect on risk of AKI (rate ratio 0.45, 95% CI 0.28, 0.72).

Conclusions: Bariatric surgery was associated with strong protective effects on AKI incidence during long-term follow-up. While the risk of AKI may be increased within the first 30 days, the net effect seen was beneficial.

Keywords: Acute Kidney Injury; Obesity; Bariatric Surgery; Clinical Practice Research Datalink

For peer review only

STRENGTHS AND LIMITATIONS OF THIS STUDY

- This study uses high quality data from linked databases in England (Clinical Practice Research Datalink and Hospital Episode Statistics) to describe long-term effects of bariatric surgery on acute kidney injury (AKI) for the first time.
- Data are captured prospectively and continuously thus allowing follow-up of patients over long time periods.
- Outcome measures are obtained with standardised ICD-10 codes, which have been shown to accurately identify AKI.
- Only AKI events recorded during a hospital admission were included in the analysis likely representing the more serious events of AKI.
- The study population was mostly female, of middle age, and had a history of type 2 diabetes mellitus. Thus the results might not be applicable for other groups suffering from obesity such as adolescents.

For peer review only

INTRODUCTION

The proportions of overweight and obese adults in England in 2014 are estimated to be 61.7% and 25.6%, respectively, and are increasing over time[1]. Obesity is associated with serious health consequences including type 2 diabetes mellitus (T2DM), cardiovascular diseases, cancers, and chronic kidney disease (CKD)[2-4]. Bariatric surgery has been shown to be a highly effective intervention for achieving weight loss and reducing the burden of co-morbidities, such as T2DM, metabolic syndrome, and hypertension[5 6]. A recent observational study on recipients of bariatric surgery from the United Kingdom (UK) confirmed sustained weight loss as well as resolution of T2DM and hypertension over a period of 4 years[7].

Acute kidney injury (AKI) is defined as a sudden (over hours or days) drop in kidney function characterised by increased serum creatinine and/or reduced urine output. AKI has been linked to increased in-hospital mortality, length of hospital stay, and subsequent development of CKD[8]. While T2DM, CKD, and obesity have been described as risk factors for AKI, it can also be precipitated by nephrotoxic drugs, surgical interventions, and sepsis[8-10]. AKI has been described as a short-term complication of bariatric surgery, stemming from rhabdomyolysis[10-16]. In addition, AKI has been linked to nephrolithiasis, which can develop over time after Roux-En-Y Gastric Bypass surgery[11 17]. To the best of our knowledge, no studies have been published examining the long-term effects of bariatric surgery on AKI.

In this study, we investigate the long-term effects of bariatric surgery on AKI to see whether the expected reduction in BMI has any impact on subsequent renal health. We used routinely collected electronic health record data from primary and secondary care. For this, we conducted a matched cohort study using prospectively collected data from patients in the United Kingdom Clinical Practice Research Datalink (CPRD).

For peer review only

METHODS

Study design

We undertook a matched cohort study using prospectively collected data from CPRD patients registered before 31st December 2014 linked to the Hospital Episodes Statistics (HES) database to investigate long-term effects of bariatric surgery on AKI.

Data source

The CPRD database contains anonymised, routinely collected data on approximately 10 million patients in participating primary care practices in the UK, including demographic characteristics, current and previous diagnoses, prescribing, test results, and lifestyle factors. Diagnoses, signs, and symptoms are recorded using Read codes[18]. Patients are broadly representative of the UK population and the data have been validated for a wide range of outcomes[19-21]. The HES database contains patient data from hospital admissions to English hospitals within the National Health Service[22]. For each hospital admission, the diagnoses are recorded using standardised codes of the International Classification of Diseases, Tenth Revision (ICD-10)[23 24]. Data from 70% of CPRD practices in England has been linked at patient level with HES admission data thus allowing the combined analysis of data from primary and acute hospital care for a subset of patients[19].

Cohort design and propensity matching

A detailed description of how the cohort was constructed is described elsewhere[7]. In brief, records of patients who underwent bariatric surgery (n=3,882) between 1997 and 2015 were matched to individuals who did not undergo surgery (n=3,882) using propensity scores.

For peer review only

For peer review only - http://bmjopen.bmj.com/site/about/guidelines.xhtml

Study population matching and the propensity score incorporated information on age, sex, calendar period, history of T2DM, hypertension, coronary heart disease, cerebrovascular disease, peripheral vascular disease, other atheroma, use of insulin, use of oral antidiabetic medication, use of statins, smoking status, and alcohol consumption.

Patients with bariatric surgery were identified using Read codes for surgery in the CPRD database (S1 Appendix) and were included in the study if they had been registered in the CPRD ≥12 months prior to the intervention. We excluded those with a record of prior bariatric surgery reversal.

For the comparison group, the inclusion criteria were to have at least one BMI measurement ≥40 kg/m^2, ≥12 months of follow-up prior to the index date in the database, and no prior record of bariatric surgery or bariatric surgery reversal.

The study sample was restricted to eligible patients registered at practices linked to the HES database and information on AKI events was obtained, resulting in a final cohort comprising 2,643 patients who underwent bariatric surgery, and 2,595 patients who did not.

Follow-up started on the day of surgery for those with bariatric surgery, and for the comparison group who did not undergo bariatric surgery, on the surgery date of their matched case. Patient records were censored at the earliest of: AKI, death, leaving the practice, latest data collection from current practice, or end of linkage period to the HES database.

Outcomes and covariates

The primary outcome of this study was the incidence rate of the first AKI episode during follow-up in patients with and without bariatric surgery. AKI episodes were obtained from the HES database using ICD-10 codes: N17.0 (“Acute kidney failure with tubular necrosis”),

For peer review only

N17.1 ("Acute renal failure with acute cortical necrosis), N17.2 ("Acute renal failure with medullary necrosis"), N17.8 ("Other acute renal failure"), N17.9 ("Acute kidney failure, unspecified"), and N19 ("Unspecified kidney failure"). In this cohort, events coded with N17.1, N17.2, and N17.8 were not found. AKI events that occurred before the start of follow-up were recorded as a binary variable "history of AKI", while AKI events occurring during follow-up were used to analyse AKI incidence.

Recorded serum creatinine values from the CPRD database were not routinely standardised with isotope-dilution mass spectrometry before 2013. Thus, we assumed all measurements to be unstandardized and multiplied the creatinine measures with the factor 0.95 before calculating the estimated glomerular filtration rate (eGFR) using the "Chronic Kidney Disease Epidemiology Collaboration" (CKD-EPI) equation[25]. Ethnicity was not considered in the eGFR calculation due to incomplete recording in the database and the low proportion of Afro-Caribbean people in the population. CKD stages were defined according to eGFR values in ml/min/1.73m^2 according to current guidelines[26]: eGFR ≥60 = no known CKD; eGFR 45-59 = stage 3a; eGFR 30-44 = stage 3b; eGFR 15-29 = stage 4; eGFR <15 = stage 5. Baseline CKD status was derived from eGFR measurements in the year prior to start of follow-up by: 1) taking the last two measurements before the index date ≥90 days apart – with the higher eGFR value corresponding to the CKD baseline status, or 2) taking the most recent serum creatinine result if only one suitable test result was available. Since serum creatinine is more likely to be tested in the acutely unwell or in people who are routinely monitored as part of incentivised programs (e.g. people with diabetes), patients without measurements of CKD baseline status were assumed to have no CKD[27] and were analysed as such.

For peer review only

Statistical Analysis

Though propensity score matching was employed to minimise confounding, we compared the distribution of baseline characteristics between the exposed and unexposed groups to check for any imbalances that may be relevant to the outcome of AKI. The baseline distribution of categorical variables was analysed using percentages and χ^2-tests. Continuous variables were analysed as means with standard deviations for normally distributed variables and medians with interquartile ranges for non-normally distributed variables. Differences in continuous variables were analysed with Student's t-tests or Wilcoxon rank sum tests for normally and non-normally distributed data, respectively.

The association between bariatric surgery and AKI was analysed using a Poisson regression model with a time to first event analysis. P-values were calculated using Wald tests. In order to separate short-term effects of the surgery from potential long-term effects, we analysed the association separately for: a) events within the first 30 days, and b) events after 30 days. When the cohort was initially constructed, propensity score matching was used to deal with confounding [7]. This study uses a subset of this cohort since patients from practices without linkage between the CPRD and HES databases had to be excluded (as AKI was assessed using hospital admission data). To identify variables for the multivariable model, potential confounders that were not deemed to be on the causal pathway were added individually to the univariable model. If the addition changed the effect estimate ≥10% these variables were included in the multivariable model. Consequently, history of AKI, history of taking oral antidiabetics, and BMI at baseline were included (S2 Appendix). In addition, age at baseline, sex, calendar period (1997-2005, 2006-2010, 2011-2015), and CKD status at baseline were selected *a priori* as forced variables. For models with <40 outcomes, only age and sex were included in the multivariable model due to data sparsity.

For peer review only

187 The 5% bands of patients with the highest and lowest propensity scores were excluded from the primary analysis ("trimming") since these contain patients that are treated in stark contrast to their health status, potentially causing bias [28].

190 Heterogeneity of effect estimates between the calendar periods was tested with a Likelihood Ratio Test.

192 The analysis was performed for all patients with bariatric surgery and also further stratified by type of surgery. Patients with stage 5 CKD (baseline eGFR < 15 ml/min/1.73m^2) were excluded from the analyses since this constitutes end-stage renal failure (ESRD). In addition, patients with missing data in ≥1 variable of the multivariable model were excluded from both uni- and multivariable analyses.

197 All analyses were performed with Stata 14.1.

198

199 **Subgroup analyses**

200 Several planned sensitivity analyses were undertaken: 1) To determine the net effect of the intervention we calculated the risk of AKI over the whole period of follow-up; 2) The prevalence of decreased kidney function in the CPRD database was similar to that in a nationally representative kidney disease registry [27] indicating that patients with missing eGFR measurements are unlikely to have CKD. To identify potential differences in the effect between patients with known and unknown eGFR measurements, we restricted the analysis to a) patients known to have no CKD at baseline (baseline eGFR ≥ 60 ml/min/1.73m^2), b) patients without known CKD at baseline (as above but including patients with missing creatinine values at baseline and assuming these individuals to have no CKD), and c) patients with known CKD at baseline.[27]; 3) Moreover, to investigate the effect in a group of particular interest which is under more scrutiny for measuring kidney function we restricted

For peer review only

the analysis to patients with: a) T2DM, and b) a history of taking insulin; 4) To avoid misclassification of low eGFR values as AKI [29] we excluded patients with stage 4 CKD at baseline; 5) We restricted the analysis to ICD-10 codes N17.0 and N17.9, which have a high positive predictive value for AKI [24]; 6) We increased the immediate post-surgery time span from 30 to 60 days; and 7) We included people with extreme propensity scores.

Ethical approval

This study was approved by the London School of Hygiene & Tropical Medicine ethics committee (LSHTM MSc Ethics Ref: 11065) and the Independent Scientific Advisory Committee on Medicines & Healthcare Products Regulatory Agency database research (approval number: 16_106R).

For peer review only

222 **RESULTS**

223 Since linkage to the HES-database was only possible for patients whose GPs had agreed for
224 their practice data to be linked to HES (S3 Appendix), there were 2,643 patients with
225 bariatric surgery and 2,595 people without surgery resulting in a cohort of overall 5,238
226 people with a median follow-up of 2.9 years (Table 1).

For peer review only

Table 1: Baseline data for CPRD/HES-linked cohort study of people with bariatric surgery and the corresponding propensity score-matched comparison cohort (data are n (%) unless otherwise specified)*

	Bariatric Surgery (n = 2,643)	Matched Comparison group without surgery (n = 2,595)	p-value[1]
**Follow-up (years), median (IQR)**	2.9 (3.2)	2.9 (3.4)	0.616
**Age (years), mean (SD)**	45.2 (10.7)	45.0 (10.8)	0.417
**17 – 39, n (%)**	818 (31.0)	826 (31.8)	
**40 – 49, n (%)**	945 (35.8)	928 (35.8)	0.727
**50 – 85, n (%)**	880 (33.3)	841 (32.4)	
**BMI at baseline, mean (SD)**	44.9 (8.9)	42.2 (6.5)	<0.001
**13 – 34, n (%)**	297 (11.2)	287 (11.1)	
**35 – 39, n (%)**	448 (17.0)	456 (17.6)	
**40 – 44, n (%)**	625 (23.7)	1,118 (43.1)	<0.001
**45 – 49, n (%)**	571 (21.6)	438 (16.9)	
**50 – 94, n (%)**	667 (25.2)	253 (9.8)	
**Missing, n (%)**	35 (1.3)	43 (1.7)	
**Female**	2,131 (80.6)	2,131 (82.1)	0.166
**History of**			
**Cerebrovascular disease**	37 (1.4)	26 (1.0)	0.186
**Coronary heart disease**	104 (3.9)	82 (3.2)	0.130
**Peripheral vascular disease**	11 (0.4)	15 (0.6)	0.405
**Other atheroma**	0	<5[2]	0.313
**T2DM**	900 (34.1)	853 (32.9)	0.365
**Taking oral antidiabetic**	571 (21.6)	455 (17.5)	<0.001
**Taking insulin**	180 (6.8)	156 (6.0)	0.238
**Hypertension**	890 (33.7)	869 (33.5)	0.886
**Statin use**	699 (26.4)	640 (24.7)	0.139
**AKI**	30 (1.1)	11 (0.4)	0.003
**Alcohol status**			
**Non-drinker**	435 (16.5)	397 (15.3)	
**Ex-drinker**	278 (10.5)	236 (9.1)	
**Current drinker (amount unknown)**	15 (0.6)	13 (0.5)	
**<2 units/day**	659 (24.9)	644 (24.8)	0.366
**3-6 units/day**	862 (32.6)	909 (35.0)	
**>6 units/day**	170 (6.4)	164 (6.3)	
**Unknown**	224 (8.5)	232 (8.9)	
**Smoking status**			
**Non-smoker**	1,126 (42.6)	1,151 (44.4)	
**Current smoker**	403 (15.3)	345 (13.3)	0.093
**Ex-smoker**	1,112 (42.1)	1,099 (42.4)	
**Unknown**	<5[2]	0	
**CKD at baseline**			
**Baseline CKD status absent**	1,119 (42.3)	1,299 (50.1)	
**No CKD**	1,470 (55.6)	1,242 (47.9)	
**Stage 3a**	27 (1.0)	37 (1.4)	<0.001
**Stage 3b**	16 (0.6)	10 (0.4)	
**Stage 4**	10 (0.4)	5 (0.2)	
**Stage 5**	<5[2]	<5[2]	
**Type of bariatric surgery**			
**Gastric band**	1,193 (45.1)		
**Sleeve gastrectomy**	364 (13.8)		
**Gastric bypass**	1,075 (40.7)		
**Other**	11 (0.4)		
**ICD-10 code for AKI during follow-up**	**n = 44**	**n = 62**	
**N17.0 (Acute kidney failure with tubular necrosis)**	<5[2]	<5[2]	
**N17.9 (Acute kidney failure, unspecified)**	38 (86.4)	52 (83.9)	0.927
**N19 (Unspecified kidney failure)**	5 (11.4)	8 (12.9)	

[1] categorical variables: χ^2-test; continuous variables: t-test + SD if normally distributed, rank sum test + IQR if non-normally distributed
[2]cell counts <5 have been suppressed to ensure anonymity
*In the original study, each surgery patient was matched 1:1 to the person without surgery with the closest propensity score, choosing matches at random where more than one possible match had the same score [7]
AKI = acute kidney injury, BMI = body mass index, CKD = chronic kidney disease, ICD-10 = International Classification of Diseases, Tenth Revision, IQR = interquartile range, SD = standard deviation, T2DM = type 2 diabetes mellitus

For peer review only

This cohort was comparable to the cohort from the original study regarding sex, mean age, mean BMI, history of T2DM, type of bariatric surgery and the imbalance of BMI at baseline [7]. More patients in the intervention group had a history of AKI compared to the comparison group (1.1% vs. 0.4%). Of the 106 included events during follow-up, 84.9% were classified with the ICD-10 code N17.9 (“acute kidney failure, unspecified”), 12.3% were coded as N19 (“unspecified kidney failure”), and 2.8% had a code of N17.0 (“Acute kidney failure with tubular necrosis”). CKD status at baseline was unknown for about half of the patients in each group with a slightly higher proportion in the unexposed group (50.1% vs. 42.3%). The majority of the patients with creatinine tests at baseline did not have CKD (96.2 %).

The number of AKI events recorded in the first 30 days of follow-up was low. All five events happened in patients with bariatric surgery and none were recorded in the control group, which is consistent with the possibility of an increased risk of AKI directly after surgery (Table 2).

For peer review only

Table 2: Association of bariatric surgery with first incident AKI, stratified by length of follow-up. Unexposed refers to the propensity matched comparison group

	PY	Events	Rate per 1000 PY (95% CI)	Crude RR (95% CI)[1]	p-value[2]	Adjusted RR (95% CI)[3]	p-value[2]
**All patients**							
**Day 1-30**							
Unexposed	203	0	0	-			
Bariatric surgery	199	5	25.1 (10.5, 60.4)	-			
**> Day 30**							
Unexposed	7,882	54	6.9 (5.2, 8.9)	-			
Bariatric surgery	8,061	34	4.2 (3.0, 5.9)	0.62 (0.40, 0.95)	0.027	0.37 (0.23, 0.61)	<0.001
**All patients analysed by type of surgery[4]**							
**Day 1-30**							
Unexposed							
Gastric band							
Sleeve gastrectomy							
Gastric bypass							
Other							
**> Day 30**							
Unexposed	7,882	54	6.9 (5.2, 8.9)	-			
Gastric band	4,614	17	3.7 (2.3, 5.9)	0.54 (0.31, 0.93)	0.026		
Sleeve gastrectomy	728	<5[5]	5.5 (2.1, 14.6)	0.80 (0.29, 2.21)	0.670		
Gastric bypass	2,655	13	4.9 (2.8, 8.4)	0.71 (0.39, 1.31)	0.277		
Other	63	0	-	-			
**All patients over whole period of follow-up**							
Unexposed	8,085	54	6.7 (5.1, 8.7)	-			
Bariatric surgery	8,259	39	4.7 (3.5, 6.5)	0.71 (0.47, 1.07)	0.099	0.45 (0.28, 0.72)	0.001

[1] Poisson regression model
[2] Wald test
[3] Poisson regression model adjusted for age at baseline, sex, calendar time, CKD at baseline, history of AKI, history of taking oral antidiabetics, and BMI at baseline
[4] No analysis for day 1-30 owing to sparse data
[5] Cell counts <5 have been suppressed to ensure anonymity
AKI = acute kidney injury, CKD = chronic kidney disease, PY = person-years, RR = rate ratio

From 30 days onwards, bariatric surgery had a protective association with AKI risk (crude RR = 0.62, 95% CI 0.40, 0.95). The effect estimate of the multivariable model indicated an even stronger protective effect associated with bariatric surgery (RR = 0.37, 95% CI 0.23, 0.61), largely due to the confounding by AKI prior to baseline.

The analysis by type of surgery yielded protective effect estimates for all types but the confidence intervals were wide and no comparison between individual procedures was feasible. Sensitivity analyses yielded similar results (S4 Appendix). A sensitivity analysis restricted to patients with known CKD at baseline could not be done owing to sparse data. Investigation of the effect of bariatric surgery over the whole follow-up period resulted in a protective net effect associated with the intervention in univariable (RR = 0.71, 95% CI 0.47, 1.07) and multivariable (RR = 0.45, 95% CI 0.28, 0.72) analyses.

For peer review only

DISCUSSION

In this study using prospectively recorded routine healthcare data from a representative sample in the UK, bariatric surgery was associated with a potentially increased risk of AKI within the first 30 days after surgery (5 events in patients with bariatric surgery, no events in control patients) but a strongly protective association thereafter (adjusted RR = 0.37, 95% CI 0.23, 0.61). The association was consistent across subgroups and sensitivity analyses. To the best of our knowledge, this is the first study to describe long-term effects of bariatric surgery on AKI.

AKI has been described as a perioperative event for bariatric surgery [12 13 15 16]. Our results are consistent with an increased risk in the early stages after surgery, however our analysis lacked enough early events to rule out chance as a reason for the results observed. Since patients do not have kidney function measures routinely checked by their family physician after bariatric surgery, many events could remain unnoticed. Patients with known CKD are more thoroughly checked for AKI and are a valuable subgroup to investigate, but the numbers in this dataset were too low to analyse.

This study uses high quality data from routine medical care in the UK. The healthcare system allows universal patient access to primary and secondary care so that the data is representative of the population. Patients are followed continuously while they are registered with a general practitioner allowing prospective data capture over long observation periods and avoiding problems with reverse causality. For the classification of AKI episodes in the HES database, the ICD-10 codes N17.0 and N17.9 comprised 87.7% of all events and have previously been shown to accurately identify AKI in a single centre study [24].

Some limitations need to be considered. Even though the data is taken from a representative sample of the UK population, the baseline data indicate that patients who

For peer review only

282 undergo bariatric surgery are mostly female, of middle age, and with a history of T2DM. 283 While the results were adjusted for age and sex they might not be applicable for other 284 groups suffering from obesity like adolescents. Linkage between the CPRD and HES 285 databases was restricted to England. However, there is no cogent reason why the results 286 should not be applicable to regions with similar healthcare systems, both in the UK and 287 internationally. We had insufficient data to determine whether the association with AKI 288 varied between different types of bariatric surgery; we found a protective effect for gastric 289 band but results were inconclusive for sleeve gastrectomy and gastric bypass.

290 Any misclassification of diagnostic codes is likely non-differential between the bariatric 291 surgery patients and the matched comparison group and would bias the effect towards the 292 null value. Another problem of primary care data is that not every patient is routinely 293 checked for their kidney function, as incentives of testing apply primarily for those at risk of 294 kidney disease due to diabetes and hypertension. The study relied on AKI events recorded in 295 HES as part of a hospital admission and over time, the awareness of the importance of AKI 296 has likely changed resulting in secular changes in recording of AKI [30]; analyses have adjusted 297 for calendar period to account for this [23 31]. Future studies with hospital creatinine data 298 should compare the AKI severity between the groups to investigate this issue. In general, 299 AKI diagnosed during hospitalisation is likely to represent more serious AKI events, though 300 we would argue these are the most clinically relevant outcomes. Moreover, a patient who 301 experienced a previous AKI episode might be under more scrutiny for detection of future 302 episodes. Since more patients in the bariatric surgery group had a history of AKI they might 303 have a higher chance of detection of an AKI episode during follow-up. This would bias the 304 estimate towards the null value and could indicate that the association we report is an 305 under-estimate.

For peer review only

In addition, CKD status at baseline was missing in almost half of the patient population. However, a recent study indicated that the prevalence of CKD in the CPRD database was comparable to that found in nationally representative registry studies [27]. This indicates that patients without eGFR-measurements at baseline are unlikely to have CKD. In addition, sensitivity analyses investigating the effect in patients with known or unknown CKD status at baseline yielded comparable results.

Since access to bariatric surgery is restricted within the UK healthcare system, some patients might have funded their operation privately, resulting in selection bias. In a recent analysis about 40% of bariatric surgery operations in the UK were privately funded [32]. Thus, the intervention group might have a higher socioeconomic status than the non-exposed group, in which similar patients would not be able to afford surgery. Since the socioeconomic background is an important determinant of health outcomes and was an unmeasured potential confounder not considered in the matching process, this could have led to more positive health outcomes in the intervention group irrespective of surgery and to an overestimation of the effect. In this study setting it was not possible to determine which patients had privately funded surgery.

Even though most baseline variables were evenly distributed due to the matching process this does not guarantee that unmeasured variables are evenly distributed as well, which can constitute residual confounding. Incorrect, imprecise, or missing measurements of covariates could also have led to residual confounding. For the multivariable model, adjusting for history of AKI led to the strongest change of the effect estimate. AKI events are likely under-recorded in the HES database, for reasons described above, and thus residual confounding is possible. Since adjusting for AKI history led to a stronger effect estimate, the

For peer review only

For peer review only - http://bmjopen.bmj.com/site/about/guidelines.xhtml

329 protective effect we report here may be an underestimate if AKI history is missing to the same degree in surgery and non-surgery patients.

This study adds to the evidence of long term effects of bariatric surgery, and appears to be the first study to quantify a long-term beneficial effect on AKI. Future studies with higher patient numbers may be able to determine differences in effect between types of surgery, investigate the effect in patients with CKD, and elucidate mechanisms of the association between bariatric surgery and AKI.

For peer review only

SUPPLEMENTARY INFORMATION

Supplementary information is available at the *BMJ open* website.

FUNDING

There was no specific funding to conduct this research project.

RM is supported by a Sir Henry Wellcome Postdoctoral Fellowship from the Wellcome Trust. KB holds a Sir Henry Dale fellowship jointly funded by the Wellcome Trust and the Royal Society. RLB is an NIHR Research Professor and supported by funding from the Rosetrees Trust and the Sir Jules Thorn Charitable Trust. LS is supported by a senior clinical fellowship from the Wellcome Trust. IJD is funded by an unrestricted grant from GlaxoSmithKline. None of the funders had any involvement in the design of the study, the data collection and analysis, the writing of the report, or the decision to submit the paper for publication.

CONFLICT OF INTEREST

The authors have no conflicts of interest to disclose.

DATA SHARING

The data were obtained from the Clinical Practice Research Datalink (CPRD). CPRD data governance does not allow us to distribute patient data to other parties. Researchers may apply for data access at www.CPRD.com. The codes used to produce the data for this study are provided in the Supporting Information.

For peer review only

361 **CONTRIBUTIONS**

362 UK, DN, RLB, IJD, and LS were responsible for conceptualisation of the study and formulate
363 the research goals and aims. UK, DN, KEM, RM, KB, RLB, LS, and IJD developed the
364 methodology and models. UK, KEM, KB, IJD, and RM worked on the data curation. UK
365 performed the statistical analysis and wrote the original draft. UK, DN, KEM, RM, KB, RLB,
366 LS, and IJD reviewed and commented the draft and gave input on editing.

For peer review only

REFERENCES

1. Public Health England. Obesity - UK and Ireland prevalence and trends. Available from: https://wwwnooorguk/NOO_about_obesity/adult_obesity/UK_prevalence_and_trends, accessed 25th August 2016.
2. Burton JO, Gray LJ, Webb DR, et al. Association of anthropometric obesity measures with chronic kidney disease risk in a non-diabetic patient population. Nephrol Dial Transplant 2012;**27**(5):1860-6.
3. Nguyen S, Hsu CY. Excess weight as a risk factor for kidney failure. Current Opinion in Nephrology and Hypertension 2007;**16**(2):71-76.
4. World Health Organisation. Obesity and overweight - Fact sheet. Available from: http://wwwwhoint/mediacentre/factsheets/fs311/en/, accessed 5th August 2016.
5. Colquitt JL, Pickett K, Loveman E, et al. Surgery for weight loss in adults. Cochrane Database Syst Rev 2014(8):CD003641.
6. Gloy VL, Briel M, Bhatt DL, et al. Bariatric surgery versus non-surgical treatment for obesity: a systematic review and meta-analysis of randomised controlled trials. BMJ 2013;**347**:f5934.
7. Douglas IJ, Bhaskaran K, Batterham RL, et al. Bariatric Surgery in the United Kingdom: A Cohort Study of Weight Loss and Clinical Outcomes in Routine Clinical Care. PLoS Med 2015;**12**(12):e1001925.
8. Lewington AK, S. Clinical Practice Guidelines - Acute Kidney Injury. UK Renal Association 2011.
9. Shashaty MG, Meyer NJ, Localio AR, et al. African American race, obesity, and blood product transfusion are risk factors for acute kidney injury in critically ill trauma patients. J Crit Care 2012;**27**(5):496-504.
10. Suneja M, Kumar AB. Obesity and perioperative acute kidney injury: A focused review. Journal of Critical Care 2014;**29**(4).
11. Currie A, Chetwood A, Ahmed AR. Bariatric surgery and renal function. Obesity Surgery 2011;**21**(4):528-39.
12. Thakar CV, Kharat V, Blanck S, et al. Acute kidney injury after gastric bypass surgery. Clin J Am Soc Nephrol 2007;**2**(3):426-30.
13. Weingarten TN, Gurrieri C, McCaffrey JM, et al. Acute kidney injury following bariatric surgery. Obesity Surgery 2013;**23**(1):64-70.
14. Chakravartty S, Sarma DR, Patel AG. Rhabdomyolysis in bariatric surgery: A Systematic review. Obesity Surgery 2013;**23**(8):1333-40.
15. Abdullah HR, Tan TP, Vaez M, et al. Predictors of Perioperative Acute Kidney Injury in Obese Patients Undergoing Laparoscopic Bariatric Surgery: a Single-Centre Retrospective Cohort Study. Obes Surg 2016;**26**(7):1493-9.
16. Sharma SK, McCauley J, Cottam D, et al. Acute changes in renal function after laparoscopic gastric surgery for morbid obesity. Surg Obes Relat Dis 2006;**2**(3):389-92.
17. Bhatti UH, Duffy AJ, Roberts KE, et al. Nephrolithiasis after bariatric surgery: A review of pathophysiologic mechanisms and procedural risk. Int J Surg 2016;**36**(Pt D):618-23.
18. Chisholm J. The Read clinical classification. BMJ 1990;**300**(6732):1092.
19. Williams T, van Staa T, Puri S, et al. Recent advances in the utility and use of the General Practice Research Database as an example of a UK Primary Care Data resource. Ther Adv Drug Saf 2012;**3**(2):89-99.

For peer review only - http://bmjopen.bmj.com/site/about/guidelines.xhtml

20. Herrett E, Gallagher AM, Bhaskaran K, et al. Data Resource Profile: Clinical Practice Research Datalink (CPRD). Int J Epidemiol 2015;**44**(3):827-36.
21. Herrett E, Thomas SL, Schoonen WM, et al. Validation and validity of diagnoses in the General Practice Research Database: a systematic review. Br J Clin Pharmacol 2010;**69**(1):4-14.
22. National Health Service. The Information Centre. Hospital Episode Statistics. Available from: http://digitalnhsuk/hes, accessed 12th August 2016.
23. McDonald HI, Shaw C, Thomas SL, et al. Methodological challenges when carrying out research on CKD and AKI using routine electronic health records. Kidney Int 2016.
24. Tomlinson LA, Riding AM, Payne RA, et al. The accuracy of diagnostic coding for acute kidney injury in England - a single centre study. BMC Nephrol 2013;**14**:58.
25. Levey AS, Stevens LA, Schmid CH, et al. A new equation to estimate glomerular filtration rate. Ann Intern Med 2009;**150**(9):604-12.
26. National Institute for Health and Care Excellence. Chronic kidney disease in adults: assessment and management, Clinical guideline. 2014.
27. Iwagami M, Tomlinson LA, Mansfield KE, et al. Validity of estimated prevalence of decreased kidney function and renal replacement therapy from primary care electronic health records compared with national survey and registry data in the United Kingdom. Nephrol Dial Transplant 2017.
28. Sturmer T, Rothman KJ, Avorn J, et al. Treatment effects in the presence of unmeasured confounding: dealing with observations in the tails of the propensity score distribution--a simulation study. Am J Epidemiol 2010;**172**(7):843-54.
29. Hill RS. Acute Kidney Injury Warning Algorithm - Best Practice Guidance. Secondary Acute Kidney Injury Warning Algorithm - Best Practice Guidance 2014. https://www.thinkkidneys.nhs.uk/wp-content/uploads/2014/12/AKI-Warning-Algorithm-Best-Practice-Guidance-final-publication-0112141.pdf.
30. McDonald HI, Shaw C, Thomas SL, et al. Methodological challenges when carrying out research on CKD and AKI using routine electronic health records. Kidney Int 2016;**90**(5):943-49.
31. James M, Pannu N. Methodological considerations for observational studies of acute kidney injury using existing data sources. J Nephrol 2009;**22**(3):295-305.
32. Gulliford MC, Charlton J, Booth HP, et al. Costs and outcomes of increasing access to bariatric surgery for obesity: cohort study and cost-effectiveness analysis using electronic health records. Southampton (UK), 2016.

For peer review only

Long-term effects of bariatric surgery on acute kidney injury: A propensity-matched cohort in the United Kingdom Clinical Practice Research Datalink

Supporting Information

Overview

S1 Appendix – Code List for Identification of patients with bariatric surgery

S2 Appendix – Association of potential confounders with bariatric surgery and AKI

S3 Appendix – Patient selection from the original cohort

S4 Appendix – Sensitivity Analyses

For peer review only

S1 Appendix

Appendix 1: Code List for identification of patients with bariatric surgery from the CPRD database as published by Douglas et al. [7]

Read code	*description*
76132.00	Laparoscopic adjustable gastric banding
76134.00	Partitioning of stomach using staples
76131.11	Mason vertical banded gastroplasty
76133.00	Partitioning of stomach using band
76116.00	Laparoscopic sleeve gastrectomy
76115.00	Sleeve gastrectomy NEC
76425.00	Duodenal switch
76135.00	Partitioning of stomach NEC
76114.00	Sleeve gastrectomy and duodenal switch
76166.00	Laparoscopic gastric bypass

For peer review only

S2 Appendix

Appendix 2: Identification of potential confounders in the association of bariatric surgery (exposure) and the endpoint of incident AKI (outcome) in patients of the linked CPRD/HES cohort

	RR (95%CI)	Change in %	Selection for multivariable model
**Crude effect estimate**	0.62 (0.40, 0.95)		
**Effect estimates when individually adjusting for**			
**Age**	0.62 (0.40, 0.95)	0.2 %	yes (a priori)
**Sex**	0.60 (0.39, 0.92)	2.7 %	yes (a priori)
**Calendar Time**	0.61 (0.40, 0.94)	0.9%	yes (a priori)
**CKD status at baseline**	0.59 (0.38, 0.91)	4.4 %	yes (a priori)
**BMI at baseline**	0.53 (0.34, 0.83)	13.9 %	yes
**Alcohol Status**	0.61 (0.40, 0.93)	1.3 %	no
**Smoking Status**	0.61 (0.40, 0.94)	0.3 %	no
**History of cerebrovascular disease**	0.61 (0.40, 0.94)	0.6 %	no
**History of coronary heart disease**	0.60 (0.39, 0.91)	3.3 %	no
**History of peripheral vascular disease**	0.64 (0.41, 0.98)	3.2 %	no
**History of other atheroma**	0.62 (0.40, 0.95)	0.0 %	no
**History of diabetes**	0.60 (0.39, 0.92)	2.7%	no
**History of taking oral antidiabetics**	0.55 (0.36, 0.85)	10.4%	yes
**History of taking insulin**	0.57 (0.37, 0.87)	7.9 %	no
**History of hypertension**	0.61 (0.40, 0.94)	1.1 %	no
**History of statin use**	0.58 (0.38, 0.89)	5.5 %	no
**History of AKI**	0.42 (0.26, 0.67)	31.9 %	yes

Variables were added individually to the univariable model testing the association between bariatric surgery and AKI. If the addition of the respective variable changed the model ≥10% then the variable was selected to be included in the multivariable model.
AKI = acute kidney injury, BMI = body mass index, CKD = chronic kidney disease

For peer review only

S3 Appendix

Appendix 3: Patient selection from the original cohort as described in Douglas et al [7]

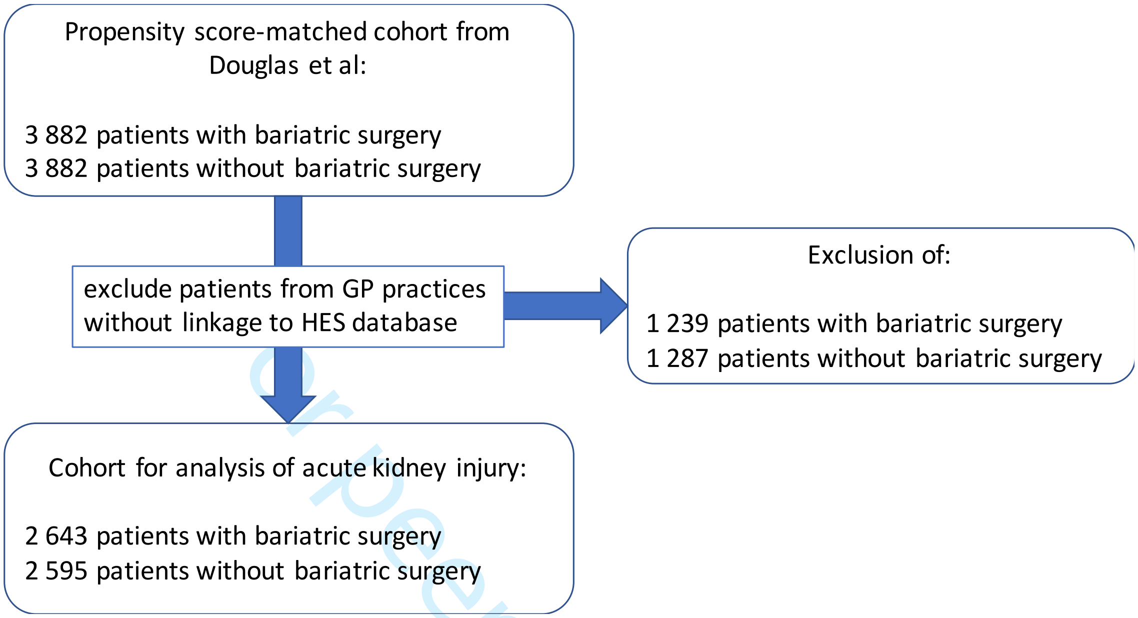

For peer review only

For peer review only - http://bmjopen.bmj.com/site/about/guidelines.xhtml

S4 Appendix

Appendix 4: Sensitivity analyses for the association of bariatric surgery with acute kidney injury

	PY	Events	Rate per 1000 PY (95% CI)	Crude RR (95% CI)[1]	p-value[2]	Adjusted RR (95% CI)[3]	p-value[2]
**Restricted to patients without CKD at baseline (available serum creatinine measures + eGFR ≥60)**							
**Day 1-30**							
Unexposed	98	0	0	-			
Bariatric surgery	111	<5[6]	36.2 (13.6, 96.3)	-			
**>Day 30**							
Unexposed	3,550	27	7.6 (5.2, 11.1)	-			
Bariatric surgery	4,311	22	5.1 (3.4, 7.7)	0.67 (0.38, 1.18)	0.165	0.53 (0.29, 1.00)	0.050
**Restricted to patients without known CKD at baseline (available serum creatinine measures + eGFR ≥60 or missing eGFR at baseline)**							
**Day 1-30**							
Unexposed	199	0	0	-			
Bariatric surgery	195	<5[6]	20.5 (7.7, 54.7)	-			
**>Day 30**							
Unexposed	7,735	42	5.4 (4.0, 7.3)	-			
Bariatric surgery	7,930	27	3.4 (2.3, 5.0)	0.63 (0.39, 1.02)	0.058	0.42 (0.25, 0.73)	0.002
**Excluding patients with CKD stage 4**							
**Day 1-30**							
Unexposed	203	0	0	-			
Bariatric surgery	198	5	25.2 (10.5, 60.6)	-			
**> Day 30**							
Unexposed	7,875	52	6.6 (5.0, 8.7)	-			
Bariatric surgery	8,037	32	4.0 (2.8, 5.6)	0.60 (0.39, 0.94)	0.024	0.35 (0.21, 0.59)	<0.001
**Restricted to patients with T2DM**							
**Day 1-30**							
Unexposed	65	0	0	-			
Bariatric surgery	69	<5[6]	43.6 (14.1, 135.1)	-			
**>Day 30**							
Unexposed	2,325	33	14.2 (10.1, 20.0)	-			
Bariatric surgery	2,548	18	7.1 (4.5, 11.2)	0.50 (0.28, 0.88)	0.017	0.25 (0.13, 0.51)	<0.001
**Restricted to patients with a history of taking insulin**							
**Day 1-30**							
Unexposed	11	0	0	-			
Bariatric surgery	13	0	0	-			
**>Day 30**							
Unexposed	321	11	34.3 (19.0, 61.9)	-			
Bariatric surgery	502	9	17.9 (9.3, 34.5)	0.52 (0.22, 1.26)	0.150	0.22 (0.08, 0.64)	0.005
**Restricted to ICD-10 codes N17.0 and N17.9**							
**Day 1-30**							
Unexposed	202	0	0	-			
Bariatric surgery	199	5	25.2 (10.5, 60.5)	-			
**>Day 30**							
Unexposed	7,871	48	6.1 (4.6, 8.1)	-			
Bariatric surgery	8,055	31	3.8 (2.7, 5.5)	0.63 (0.40, 0.99)	0.046	0.40 (0.24, 0.67)	<0.001
**Having an initial post-surgery time span of 60 days instead of 30**							
**Day 1-60**							
Unexposed	403	<5[6]	2.5 (0.3, 17.6)	-			
Bariatric surgery	395	6	15.2 (6.8, 33.8)	6.11 (0.74, 50.8)	0.094	[4]	
**> Day 60**							
Unexposed	7,682	53	6.9 (5.3, 9.0)	-			
Bariatric surgery	7,864	33	4.2 (3.0, 5.9)	0.61 (0.39, 0.94)	0.025	0.38 (0.23, 0.63)	<0.001
				Test for interaction[5]	0.011		

For peer review only

**Including patients with extreme propensity scores**							
**Day 1-30**							
Unexposed	208	0	0	-			
Bariatric surgery	206	5	24.3 (10.1, 58.3)	-			
**> Day 30**							
Unexposed	8,054	59	7.3 (5.7, 9.5)	-			
Bariatric surgery	8,324	34	4.1 (2.9, 5.7)	0.56 (0.37, 0.85)	0.007	0.33 (0.20, 0.54)	<0.001

[1] Poisson regression model
[2] Wald test for RR, Likelihood-Ratio Test for interaction
[3] Poisson regression model adjusted for age at baseline, sex, calendar time, CKD at baseline, history of AKI, history of taking oral antidiabetics, and BMI at baseline
[4] No analysis for day 1-30 owing to sparse data
[5] Test for interaction of the effect estimate with the time periods 1-30 days and >30 days
[6] cell counts <5 have been suppressed to ensure anonymity
AKI = acute kidney injury, CKD = chronic kidney disease, PY = person-years, RR = rate ratio

For peer review only

Supporting Information:
STROBE statement checklist to ensure appropriate reporting of study information of long-term effects of acute kidney injury for the propensity-matched cohort study of patients with and without bariatric surgery

	Item No	Report
**Title and abstract**	1	(*a*) Indicate the study's design with a commonly used term in the title or the abstract<br><br>"Long-term effects of bariatric surgery on acute kidney injury: A propensity-matched cohort in the United Kingdom Clinical Practice Research Datalink"
		b) Provide in the abstract an informative and balanced summary of what was done and what was found<br><br>Abstract: on page 2 containing Background, Methods, Results and Conclusions
**Introduction**		
Background/rationale	2	Explain the scientific background and rationale for the investigation being reported<br><br>See page 4 for description of background;<br>Rationale (p4): "To the best of our knowledge, no studies have been published examining the long-term effects of bariatric surgery on AKI."
Objectives	3	State specific objectives, including any prespecified hypotheses<br><br>Page 4: "In this study, we investigate the long-term effects of bariatric surgery on AKI to see whether the expected reduction in BMI has any impact on subsequent renal health."
**Methods**		
Study design	4	Present key elements of study design early in the paper<br><br>See page 5: "We undertook a matched cohort study using prospectively collected data from CPRD patients registered before 31st December 2014 linked to the Hospital Episodes Statistics (HES) database to investigate long-term effects of bariatric surgery on AKI."
Setting	5	Describe the setting, locations, and relevant dates, including periods of recruitment, exposure, follow-up, and data collection<br><br>Page 5: "The CPRD database contains anonymised, routinely collected data on approximately 10 million patients in participating primary care practices in the UK, including demographic characteristics, current and previous diagnoses, prescribing, test results, and lifestyle factors. […].The HES database contains patient data from hospital admissions to English hospitals within the National Health Service […].Data from 70% of CPRD practices in England has been linked at patient level with HES admission data thus allowing the combined analysis of data from primary and acute hospital care for a subset of patients."<br>"A detailed description of how the cohort was constructed is described elsewhere. In brief, records of patients who underwent bariatric surgery (n=3,882) between 1997 and 2015 were matched to individuals who did not undergo surgery (n=3,882) using propensity scores."<br>Page 6: "Follow-up started on the day of surgery for those with bariatric surgery, and for the comparison group who did not undergo bariatric surgery, on the surgery date

For peer review only - http://bmjopen.bmj.com/site/about/guidelines.xhtml

		of their matched case. Patient records were censored at the earliest of: AKI, death, leaving the practice, latest data collection from current practice, or end of linkage period to the HES database.”
Participants	6	(*a*) Give the eligibility criteria, and the sources and methods of selection of participants. Describe methods of follow-up<br><br>Page 6: “Patients with bariatric surgery were identified using Read codes for surgery in the CPRD database (S1 Appendix) and were included in the study if they had been registered in the CPRD ≥12 months prior to the intervention. We excluded those with a record of prior bariatric surgery reversal.<br>For the comparison group, the inclusion criteria were to have at least one BMI measurement ≥40 kg/m2, ≥12 months of follow-up prior to the index date in the database, and no prior record of bariatric surgery or bariatric surgery reversal.”<br><br>Page 5: “The CPRD database contains anonymised, routinely collected data on approximately 10 million patients in participating primary care practices in the UK, including demographic characteristics, current and previous diagnoses, prescribing, test results, and lifestyle factors. Diagnoses, signs, and symptoms are recorded using Read codes […]. The HES database contains patient data from hospital admissions to English hospitals within the National Health Service “
		(*b*) For matched studies, give matching criteria and number of exposed and unexposed<br><br>Page 6: “Study population matching and the propensity score incorporated information on age, sex, calendar period, history of T2DM, hypertension, coronary heart disease, cerebrovascular disease, peripheral vascular disease, other atheroma, use of insulin, use of oral antidiabetic medication, use of statins, smoking status, and alcohol consumption.”<br>“The study sample was restricted to eligible patients registered at practices linked to the HES database and information on AKI events was obtained, resulting in a final cohort comprising 2,643 patients who underwent bariatric surgery, and 2,595 patients who did not.”
Variables	7	Clearly define all outcomes, exposures, predictors, potential confounders, and effect modifiers. Give diagnostic criteria, if applicable<br><br>Page 6/7: “AKI episodes were obtained from the HES database using ICD-10 codes: N17.0 (“Acute kidney failure with tubular necrosis”), N17.1 (“Acute renal failure with acute cortical necrosis), N17.2 (“Acute renal failure with medullary necrosis”), N17.8 (“Other acute renal failure”), N17.9 (“Acute kidney failure, unspecified”), and N19 (“Unspecified kidney failure”). In this cohort, events coded with N17.1, N17.2, and N17.8 were not found. AKI events that occurred before the start of follow-up were recorded as a binary variable “history of AKI”, while AKI events occurring during follow-up were used to analyse AKI incidence.<br>Recorded serum creatinine values from the CPRD database were not routinely standardised with isotope-dilution mass spectrometry before 2013. Thus, we assumed all measurements to be unstandardized and multiplied the creatinine measures with the factor 0.95 before calculating the estimated glomerular filtration rate (eGFR) using the “Chronic Kidney Disease Epidemiology Collaboration” (CKD-EPI) equation.

For peer review only

For peer review only - http://bmjopen.bmj.com/site/about/guidelines.xhtml

		Ethnicity was not considered in the eGFR calculation due to incomplete recording in the database and the low proportion of Afro-Caribbean people in the population. CKD stages were defined according to eGFR values in ml/min/1.73m^2 according to current guidelines: eGFR ≥60 = no known CKD; eGFR 45-59 = stage 3a; eGFR 30-44 = stage 3b; eGFR 15-29 = stage 4; eGFR <15 = stage 5. Baseline CKD status was derived from eGFR measurements in the year prior to start of follow-up by: 1) taking the last two measurements before the index date ≥90 days apart – with the higher eGFR value corresponding to the CKD baseline status, or 2) taking the most recent serum creatinine result if only one suitable test result was available. Since serum creatinine is more likely to be tested in the acutely unwell or in people who are routinely monitored as part of incentivised programs (e.g. people with diabetes), patients without measurements of CKD baseline status were assumed to have no CKD and were analysed as such."<br>Page 6: "Study population matching and the propensity score incorporated information on age, sex, calendar period, history of T2DM, hypertension, coronary heart disease, cerebrovascular disease, peripheral vascular disease, other atheroma, use of insulin, use of oral antidiabetic medication, use of statins, smoking status, and alcohol consumption."<br>Page 8: "When the cohort was initially constructed, propensity score matching was used to deal with confounding. This study uses a subset of this cohort since patients from practices without linkage between the CPRD and HES databases had to be excluded (as AKI was assessed using hospital admission data). To identify variables for the multivariable model, potential confounders that were not deemed to be on the causal pathway were added individually to the univariable model. If the addition changed the effect estimate ≥10% these variables were included in the multivariable model. Consequently, history of AKI, history of taking oral antidiabetics, and BMI at baseline were included (S2 Appendix). In addition, age at baseline, sex, calendar period (1997-2005, 2006-2010, 2011-2015), and CKD status at baseline were selected a priori as forced variables."<br>Page 8: "In order to separate short-term effects of the surgery from potential long-term effects, we analysed the association separately for: a) events within the first 30 days, and b) events after 30 days."
Data sources/ measurement	8*	For each variable of interest, give sources of data and details of methods of assessment (measurement). Describe comparability of assessment methods if there is more than one group<br><br>Page 5: "The CPRD database contains anonymised, routinely collected data on approximately 10 million patients in participating primary care practices in the UK, including demographic characteristics, current and previous diagnoses, prescribing, test results, and lifestyle factors. Diagnoses, signs, and symptoms are recorded using Read codes […]The HES database contains patient data from hospital admissions to English hospitals within the National Health Service. For each hospital admission, the diagnoses are recorded using standardised codes of the International Classification of Diseases, Tenth Revision (ICD-10). Data from 70% of CPRD practices in England has been linked at patient level with HES admission data thus allowing the combined analysis of data from primary and acute hospital care for a subset of patients."<br>Page 5: "Patients with bariatric surgery were identified using Read codes for surgery in the CPRD database (S1 Appendix) and were included in the study if they had been

For peer review only

		registered in the CPRD ≥12 months prior to the intervention. We excluded those with a record of prior bariatric surgery reversal."<br>Page 6/7: "AKI episodes were obtained from the HES database using ICD-10 codes: N17.0 ("Acute kidney failure with tubular necrosis"), N17.1 ("Acute renal failure with acute cortical necrosis), N17.2 ("Acute renal failure with medullary necrosis"), N17.8 ("Other acute renal failure"), N17.9 ("Acute kidney failure, unspecified"), and N19 ("Unspecified kidney failure")."
Bias	9	Describe any efforts to address potential sources of bias<br><br>Page 9: "The 5% bands of patients with the highest and lowest propensity scores were excluded from the primary analysis ("trimming") since these contain patients that are treated in stark contrast to their health status, potentially causing bias."
Study size	10	Explain how the study size was arrived at<br><br>Pages 5/6: "A detailed description of how the cohort was constructed is described elsewhere. In brief, records of patients who underwent bariatric surgery (n=3,882) between 1997 and 2015 were matched to individuals who did not undergo surgery (n=3,882) using propensity scores." […] "The study sample was restricted to eligible patients registered at practices linked to the HES database and information on AKI events was obtained, resulting in a final cohort comprising 2,643 patients who underwent bariatric surgery, and 2,595 patients who did not."
Quantitative variables	11	Explain how quantitative variables were handled in the analyses. If applicable, describe which groupings were chosen and why<br><br>Page 7: "Recorded serum creatinine values from the CPRD database were not routinely standardised with isotope-dilution mass spectrometry before 2013. Thus, we assumed all measurements to be unstandardized and multiplied the creatinine measures with the factor 0.95 before calculating the estimated glomerular filtration rate (eGFR) using the "Chronic Kidney Disease Epidemiology Collaboration" (CKD-EPI) equation. Ethnicity was not considered in the eGFR calculation due to incomplete recording in the database and the low proportion of Afro-Caribbean people in the population. CKD stages were defined according to eGFR values in ml/min/1.73m2 according to current guidelines: eGFR ≥60 = no known CKD; eGFR 45-59 = stage 3a; eGFR 30-44 = stage 3b; eGFR 15-29 = stage 4; eGFR <15 = stage 5. Baseline CKD status was derived from eGFR measurements in the year prior to start of follow-up by: 1) taking the last two measurements before the index date ≥90 days apart – with the higher eGFR value corresponding to the CKD baseline status, or 2) taking the most recent serum creatinine result if only one suitable test result was available. Since serum creatinine is more likely to be tested in the acutely unwell or in people who are routinely monitored as part of incentivised programs (e.g. people with diabetes), patients without measurements of CKD baseline status were assumed to have no CKD and were analysed as such."<br>Page 8: "In addition, age at baseline, sex, calendar period (1997-2005, 2006-2010, 2011-2015), and CKD status at baseline were selected a priori as forced variables."
Statistical methods	12	(*a*) Describe all statistical methods, including those used to control for confounding<br><br>Page 8/9: "The association between bariatric surgery and AKI was analysed using a Poisson regression model with a time to first event analysis. P-values were calculated

For peer review only - http://bmjopen.bmj.com/site/about/guidelines.xhtml

using Wald tests. In order to separate short-term effects of the surgery from potential long-term effects, we analysed the association separately for: a) events within the first 30 days, and b) events after 30 days. When the cohort was initially constructed, propensity score matching was used to deal with confounding. This study uses a subset of this cohort since patients from practices without linkage between the CPRD and HES databases had to be excluded (as AKI was assessed using hospital admission data). To identify variables for the multivariable model, potential confounders that were not deemed to be on the causal pathway were added individually to the univariable model. If the addition changed the effect estimate ≥10% these variables were included in the multivariable model. Consequently, history of AKI, history of taking oral antidiabetics, and BMI at baseline were included (S2 Appendix). In addition, age at baseline, sex, calendar period (1997-2005, 2006-2010, 2011-2015), and CKD status at baseline were selected a priori as forced variables. For models with <40 outcomes, only age and sex were included in the multivariable model due to data sparsity.

The 5% bands of patients with the highest and lowest propensity scores were excluded from the primary analysis ("trimming") since these contain patients that are treated in stark contrast to their health status, potentially causing bias.

Heterogeneity of effect estimates between the calendar periods was tested with a Likelihood Ratio Test.

The analysis was performed for all patients with bariatric surgery and also further stratified by type of surgery. Patients with stage 5 CKD (baseline eGFR < 15 ml/min/1.73m2) were excluded from the analyses since this constitutes end-stage renal failure (ESRD). In addition, patients with missing data in ≥1 variable of the multivariable model were excluded from both uni- and multivariable analyses."

(*b*) Describe any methods used to examine subgroups and interactions

page 8: "In order to separate short-term effects of the surgery from potential long-term effects, we analysed the association separately for: a) events within the first 30 days, and b) events after 30 days."

page 9: "Heterogeneity of effect estimates between the calendar periods was tested with a Likelihood Ratio Test."

Page 9: "The analysis was performed for all patients with bariatric surgery and also further stratified by type of surgery."

(*c*) Explain how missing data were addressed

page 7: "Since serum creatinine is more likely to be tested in the acutely unwell or in people who are routinely monitored as part of incentivised programs (e.g. people with diabetes), patients without measurements of CKD baseline status were assumed to have no CKD and were analysed as such."

page 9: "In addition, patients with missing data in ≥1 variable of the multivariable model were excluded from both uni- and multivariable analyses."

(*d*) If applicable, explain how loss to follow-up was addressed

page 6: "Patient records were censored at the earliest of: AKI, death, leaving the practice, latest data collection from current practice, or end of linkage period to the

For peer review only - http://bmjopen.bmj.com/site/about/guidelines.xhtml

		HES database."
		(*e*) Describe any sensitivity analyses<br><br>Pages 9/10: "Several planned sensitivity analyses were undertaken: 1) To determine the net effect of the intervention we calculated the risk of AKI over the whole period of follow-up; 2) The prevalence of decreased kidney function in the CPRD database was similar to that in a nationally representative kidney disease registry indicating that patients with missing eGFR measurements are unlikely to have CKD. To identify potential differences in the effect between patients with known and unknown eGFR measurements, we restricted the analysis to a) patients known to have no CKD at baseline (baseline eGFR ≥ 60 ml/min/1.73m^2), b) patients without known CKD at baseline (as above but including patients with missing creatinine values at baseline and assuming these individuals to have no CKD), and c) patients with known CKD at baseline.; 3) Moreover, to investigate the effect in a group of particular interest which is under more scrutiny for measuring kidney function we restricted the analysis to patients with: a) T2DM, and b) a history of taking insulin; 4) To avoid misclassification of low eGFR values as AKI we excluded patients with stage 4 CKD at baseline; 5) We restricted the analysis to ICD-10 codes N17.0 and N17.9, which have a high positive predictive value for AKI; 6) We increased the immediate post-surgery time span from 30 to 60 days; and 7) We included people with extreme propensity scores."
**Results**		
Participants	13*	(a) Report numbers of individuals at each stage of study—eg numbers potentially eligible, examined for eligibility, confirmed eligible, included in the study, completing follow-up, and analysed<br><br>see S3 Appendix
		(b) Give reasons for non-participation at each stage<br><br>not applicable
		(c) Consider use of a flow diagram<br><br>see S3 Appendix
Descriptive data	14*	(a) Give characteristics of study participants (eg demographic, clinical, social) and information on exposures and potential confounders<br><br>page 12: see Table 1
		(b) Indicate number of participants with missing data for each variable of interest<br><br>page 12: see Table 1
		(c) Summarise follow-up time (eg, average and total amount)<br><br>page 12: see Table 1
Outcome data	15*	Report numbers of outcome events or summary measures over time<br><br>page 12: see Table 1
Main results	16	(*a*) Give unadjusted estimates and, if applicable, confounder-adjusted estimates and their precision (eg, 95% confidence interval). Make clear which confounders were

For peer review only - http://bmjopen.bmj.com/site/about/guidelines.xhtml

		adjusted for and why they were included<br><br>page 13: see Table 2
		(*b*) Report category boundaries when continuous variables were categorized<br><br>page 12: see Table 1
		(*c*) If relevant, consider translating estimates of relative risk into absolute risk for a meaningful time period<br><br>not applicable
Other analyses	17	Report other analyses done—eg analyses of subgroups and interactions, and sensitivity analyses<br><br>Page 13: see table 2<br>see S4 Appendix
**Discussion**		
Key results	18	Summarise key results with reference to study objectives<br><br>Page 15: "In this study using prospectively recorded routine healthcare data from a representative sample in the UK, bariatric surgery was associated with a potentially increased risk of AKI within the first 30 days after surgery (5 events in patients with bariatric surgery, no events in control patients) but a strongly protective association thereafter (adjusted RR = 0.37, 95% CI 0.23, 0.61). The association was consistent across subgroups and sensitivity analyses. To the best of our knowledge, this is the first study to describe long-term effects of bariatric surgery on AKI."
Limitations	19	Discuss limitations of the study, taking into account sources of potential bias or imprecision. Discuss both direction and magnitude of any potential bias<br><br>Pages 15-18: "Some limitations need to be considered. Even though the data is taken from a representative sample of the UK population, the baseline data indicate that patients who undergo bariatric surgery are mostly female, of middle age, and with a history of T2DM. While the results were adjusted for age and sex they might not be applicable for other groups suffering from obesity like adolescents. Linkage between the CPRD and HES databases was restricted to England. However, there is no cogent reason why the results should not be applicable to regions with similar healthcare systems, both in the UK and internationally. We had insufficient data to determine whether the association with AKI varied between different types of bariatric surgery; we found a protective effect for gastric band but results were inconclusive for sleeve gastrectomy and gastric bypass.<br>Any misclassification of diagnostic codes is likely non-differential between the bariatric surgery patients and the matched comparison group and would bias the effect towards the null value. Another problem of primary care data is that not every patient is routinely checked for their kidney function, as incentives of testing apply primarily for those at risk of kidney disease due to diabetes and hypertension. The study relied on AKI events recorded in HES as part of a hospital admission and over time, the awareness of the importance of AKI has likely changed resulting in secular changes in recording of AKI ; analyses have adjusted for calendar period to account for this. Future studies with hospital creatinine data should compare the AKI severity between the groups to investigate this issue. In general, AKI diagnosed during hospitalisation

For peer review only

		is likely to represent more serious AKI events, though we would argue these are the most clinically relevant outcomes. Moreover, a patient who experienced a previous AKI episode might be under more scrutiny for detection of future episodes. Since more patients in the bariatric surgery group had a history of AKI they might have a higher chance of detection of an AKI episode during follow-up. This would bias the estimate towards the null value and could indicate that the association we report is an under-estimate.<br>In addition, CKD status at baseline was missing in almost half of the patient population. However, a recent study indicated that the prevalence of CKD in the CPRD database was comparable to that found in nationally representative registry studies. This indicates that patients without eGFR-measurements at baseline are unlikely to have CKD. In addition, sensitivity analyses investigating the effect in patients with known or unknown CKD status at baseline yielded comparable results.<br>Since access to bariatric surgery is restricted within the UK healthcare system, some patients might have funded their operation privately, resulting in selection bias. In a recent analysis about 40% of bariatric surgery operations in the UK were privately funded. Thus, the intervention group might have a higher socioeconomic status than the non-exposed group, in which similar patients would not be able to afford surgery. Since the socioeconomic background is an important determinant of health outcomes and was an unmeasured potential confounder not considered in the matching process, this could have led to more positive health outcomes in the intervention group irrespective of surgery and to an overestimation of the effect. In this study setting it was not possible to determine which patients had privately funded surgery.<br>Even though most baseline variables were evenly distributed due to the matching process this does not guarantee that unmeasured variables are evenly distributed as well, which can constitute residual confounding. Incorrect, imprecise, or missing measurements of covariates could also have led to residual confounding. For the multivariable model, adjusting for history of AKI led to the strongest change of the effect estimate. AKI events are likely under-recorded in the HES database, for reasons described above, and thus residual confounding is possible. Since adjusting for AKI history led to a stronger effect estimate, the protective effect we report here may be an underestimate if AKI history is missing to the same degree in surgery and non-surgery patients.“
Interpretation	20	Give a cautious overall interpretation of results considering objectives, limitations, multiplicity of analyses, results from similar studies, and other relevant evidence<br><br>Page 18: “This study adds to the evidence of long term effects of bariatric surgery, and appears to be the first study to quantify a long-term beneficial effect on AKI. Future studies with higher patient numbers may be able to determine differences in effect between types of surgery, investigate the effect in patients with CKD, and elucidate mechanisms of the association between bariatric surgery and AKI.”
Generalisability	21	Discuss the generalisability (external validity) of the study results<br><br>Page 15/16: “Even though the data is taken from a representative sample of the UK population, the baseline data indicate that patients who undergo bariatric surgery are mostly female, of middle age, and with a history of T2DM. While the results were adjusted for age and sex they might not be applicable for other groups suffering from obesity like adolescents. Linkage between the CPRD and HES databases was restricted to England. However, there is no cogent reason why the results should not

For peer review only

For peer review only - http://bmjopen.bmj.com/site/about/guidelines.xhtml

		be applicable to regions with similar healthcare systems, both in the UK and internationally."
**Other information**		
Funding	22	Give the source of funding and the role of the funders for the present study and, if applicable, for the original study on which the present article is based<br><br>RM is supported by a Sir Henry Wellcome Postdoctoral Fellowship from the Wellcome Trust. KB holds a Sir Henry Dale fellowship jointly funded by the Wellcome Trust and the Royal Society. RLB is an NIHR Research Professor and supported by funding from the Rosetrees Trust and the Sir Jules Thorn Charitable Trust. LS is supported by a senior clinical fellowship from the Wellcome Trust. IJD is funded by an unrestricted grant from GlaxoSmithKline.

*Give information separately for exposed and unexposed groups.

Note: An Explanation and Elaboration article discusses each checklist item and gives methodological background and published examples of transparent reporting. The STROBE checklist is best used in conjunction with this article (freely available on the Web sites of PLoS Medicine at http://www.plosmedicine.org/, Annals of Internal Medicine at http://www.annals.org/, and Epidemiology at http://www.epidem.com/). Information on the STROBE Initiative is available at http://www.strobe-statement.org.

For peer review only

BMJ Open

Long-term effects of bariatric surgery on acute kidney injury: A propensity-matched cohort in the United Kingdom Clinical Practice Research Datalink

Journal:	*BMJ Open*
Manuscript ID	bmjopen-2017-020371.R1
Article Type:	Research
Date Submitted by the Author:	15-Dec-2017

Complete List of Authors:	Koppe, Uwe; Robert Koch Institut,<br>Nitsch, Dorothea; LSHTM; Royal Free London NHS Foundation Trust, Honorary consultant nephrologist<br>Mansfield, Kathryn; London School of Hygiene and Tropical Medicine, Non-communicable Disease Epidemiology<br>Mathur, Rohini; London School of Hygiene and Tropical Medicine, Department of Non-Communicable Disease Epidemiology; Queen Mary University of London, Centre for Primary Care & Public Health<br>Bhaskaran, Krishnan; LSHTM, NCDE<br>Batterham, Rachel; UCL, Centre for Obesity Research, Rayne Institute, Department of Medicine<br>Smeeth, Liam; London School of Hygiene and Tropical Medicine, Epidemiology and Population Health<br>Douglas, Ian; London School of Hygiene and Tropical Medicine, Epidemiology and Population Health

**Primary Subject Heading**:	Epidemiology
Secondary Subject Heading:	Renal medicine
Keywords:	Clinical Practice Research Datalink, Acute Kidney Injury, Obesity, Bariatric Surgery

SCHOLARONE™ Manuscripts

Title Long-term effects of bariatric surgery on acute kidney injury: A propensity-matched cohort in the United Kingdom Clinical Practice Research Datalink

Running headline Bariatric surgery and acute kidney injury

Authors Uwe Koppe[1,2*], Dorothea Nitsch[1,3], Kathryn E. Mansfield[1], Rohini Mathur[1], Krishnan Bhaskaran[1], Rachel L. Batterham[4,5,6], Liam Smeeth[1], Ian J. Douglas[1]

[1] Faculty of Epidemiology and Population Health, London School of Hygiene & Tropical Medicine, London, United Kingdom,

[2] Department of Infectious Disease Epidemiology, Robert Koch Institute, Berlin, Germany,

[3] Royal Free London NHS Trust, Honorary consultant nephrologist

[4] Centre for Obesity Research, Rayne Institute, Department of Medicine, University College London, London, United Kingdom,

[5] University College London Hospital Bariatric Centre for Weight Management and Metabolic Surgery, London, United Kingdom,

[6] National Institute of Health Research, University College London Hospital Biomedical Research Centre, London, United Kingdom

* Correspondence to:

Dr. Uwe Koppe
Robert Koch Institut
Seestr. 10
13353 Berlin, Germany
Tel: +49 30 18754 2262
Fax: +49 30 18754 3533
koppeu@rki.de

Word count: 3,421

For peer review only

ABSTRACT

Objective: Bariatric surgery is an effective method of weight reduction and has been associated with acute kidney injury (AKI) as a perioperative event. However, the long-term effects of the weight reduction after surgery on AKI are unknown. The objective of this study is to quantify the association of bariatric surgery with later risk of AKI.

Design: This study uses a propensity-score matched cohort of patients from the United Kingdom Clinical Practice Research Datalink database with and without bariatric surgery to compare rates of AKI episodes derived from linkage to the Hospital Episode Statistics.

Setting: England, United Kingdom

Participants: We included 2,643 patients with bariatric surgery and 2,595 patients without.

Results: Results were compatible with an increased risk of AKI in the first 30 days following surgery compared with patients without surgery, but AKI incidence was substantially decreased in patients with bariatric surgery during long-term follow-up (rate ratio 0.37, 95% CI 0.23, 0.61) even after accounting for chronic kidney disease status at baseline. Over the whole period of follow-up, bariatric surgery had a net protective effect on risk of AKI (rate ratio 0.45, 95% CI 0.28, 0.72).

Conclusions: Bariatric surgery was associated with strong protective effects on AKI incidence during long-term follow-up. While the risk of AKI may be increased within the first 30 days, the net effect seen was beneficial.

Keywords: Acute Kidney Injury; Obesity; Bariatric Surgery; Clinical Practice Research Datalink

For peer review only

STRENGTHS AND LIMITATIONS OF THIS STUDY

- This study uses high quality data from linked databases in England (Clinical Practice Research Datalink and Hospital Episode Statistics) to describe long-term effects of bariatric surgery on acute kidney injury (AKI) for the first time.
- Data are captured prospectively and continuously thus allowing follow-up of patients over long time periods.
- Outcome measures are obtained with standardised ICD-10 codes, which have been shown to accurately identify AKI.
- Only AKI events recorded during a hospital admission were included in the analysis likely representing the more serious events of AKI.
- The study population was mostly female, of middle age, and had a history of type 2 diabetes mellitus. Thus the results might not be applicable for other groups suffering from obesity such as adolescents.

For peer review only

INTRODUCTION

The proportions of overweight and obese adults in England in 2014 are estimated to be 61.7% and 25.6%, respectively, and are increasing over time[1]. Obesity is associated with serious health consequences including type 2 diabetes mellitus (T2DM), cardiovascular diseases, cancers, and chronic kidney disease (CKD)[2-4]. Bariatric surgery has been shown to be a highly effective intervention for achieving weight loss and reducing the burden of co-morbidities, such as T2DM, metabolic syndrome, and hypertension[5 6]. A recent observational study on recipients of bariatric surgery from the United Kingdom (UK) confirmed sustained weight loss as well as resolution of T2DM and hypertension over a period of 4 years[7].

Acute kidney injury (AKI) is defined as a sudden (over hours or days) drop in kidney function characterised by increased serum creatinine and/or reduced urine output. AKI has been linked to increased in-hospital mortality, length of hospital stay, and subsequent development of CKD[8]. While T2DM, CKD, and obesity have been described as risk factors for AKI, it can also be precipitated by nephrotoxic drugs, surgical interventions, and sepsis[8-10]. AKI has been described as a short-term complication of bariatric surgery, stemming from rhabdomyolysis[10-16]. In addition, AKI has been linked to nephrolithiasis, which can develop over time after Roux-En-Y Gastric Bypass surgery[11 17]. To the best of our knowledge, no studies have been published examining the long-term effects of bariatric surgery on AKI.

In this study, we investigate the long-term effects of bariatric surgery on AKI to see whether the expected reduction in BMI has any impact on subsequent renal health. We used routinely collected electronic health record data from primary and secondary care. For this, we conducted a matched cohort study using prospectively collected data from patients in the United Kingdom Clinical Practice Research Datalink (CPRD).

For peer review only

METHODS

Study design

We undertook a matched cohort study using prospectively collected data from CPRD patients registered before 31st December 2014 linked to the Hospital Episodes Statistics (HES) database to investigate long-term effects of bariatric surgery on AKI.

Data source

The CPRD database contains anonymised, routinely collected data on approximately 10 million patients in participating primary care practices in the UK, including demographic characteristics, current and previous diagnoses, prescribing, test results, and lifestyle factors. Diagnoses, signs, and symptoms are recorded using Read codes[18]. Patients are broadly representative of the UK population and the data have been validated for a wide range of outcomes[19-21]. The HES database contains patient data from hospital admissions to English hospitals within the National Health Service[22]. For each hospital admission, the diagnoses are recorded using standardised codes of the International Classification of Diseases, Tenth Revision (ICD-10)[23 24]. Data from 70% of CPRD practices in England has been linked at patient level with HES admission data thus allowing the combined analysis of data from primary and acute hospital care for a subset of patients[19].

Cohort design and propensity matching

A detailed description of how the cohort was constructed is described elsewhere[7]. In brief, records of patients who underwent bariatric surgery (n=3,882) between 1997 and 2015 were matched to individuals who did not undergo surgery (n=3,882) using propensity scores.

For peer review only

Study population matching and the propensity score incorporated information on age, sex, calendar period, history of T2DM, hypertension, coronary heart disease, cerebrovascular disease, peripheral vascular disease, other atheroma, use of insulin, use of oral antidiabetic medication, use of statins, smoking status, and alcohol consumption.

Patients with bariatric surgery were identified using Read codes for surgery in the CPRD database (S1 Appendix) and were included in the study if they had been registered in the CPRD ≥12 months prior to the intervention. We excluded those with a record of prior bariatric surgery reversal.

For the comparison group, the inclusion criteria were to have at least one BMI measurement ≥40 kg/m^2 during their CPRD registration, which could span 10 years or more, ≥12 months of follow-up prior to the index date in the database, and no prior record of bariatric surgery or bariatric surgery reversal. Based on this, it is therefore possible that the BMI recorded closest to the index date was lower than 40 kg/m^2.

The study sample was restricted to eligible patients registered at practices linked to the HES database and information on AKI events was obtained, resulting in a final cohort comprising 2,643 patients who underwent bariatric surgery, and 2,595 patients who did not.

Follow-up started on the day of surgery for those with bariatric surgery, and for the comparison group who did not undergo bariatric surgery, on the surgery date of their matched case. Patient records were censored at the earliest of: AKI, death, leaving the practice, latest data collection from current practice, or end of linkage period to the HES database.

For peer review only - http://bmjopen.bmj.com/site/about/guidelines.xhtml

Outcomes and covariates

The primary outcome of this study was the incidence rate of the first AKI episode during follow-up in patients with and without bariatric surgery. AKI episodes were obtained from the HES database using ICD-10 codes: N17.0 (“Acute kidney failure with tubular necrosis”), N17.1 (“Acute renal failure with acute cortical necrosis), N17.2 (“Acute renal failure with medullary necrosis”), N17.8 (“Other acute renal failure”), N17.9 (“Acute kidney failure, unspecified”), and N19 (“Unspecified kidney failure”). In this cohort, events coded with N17.1, N17.2, and N17.8 were not found. AKI events that occurred before the start of follow-up were recorded as a binary variable “history of AKI”, while AKI events occurring during follow-up were used to analyse AKI incidence.

Recorded serum creatinine values from the CPRD database were not routinely standardised with isotope-dilution mass spectrometry before 2013. Thus, we assumed all measurements to be unstandardized and multiplied the creatinine measures with the factor 0.95 before calculating the estimated glomerular filtration rate (eGFR) using the “Chronic Kidney Disease Epidemiology Collaboration” (CKD-EPI) equation[25]. Ethnicity was not considered in the eGFR calculation due to incomplete recording in the database and the low proportion of Afro-Caribbean people in the population. CKD stages were defined according to eGFR values in ml/min/1.73m^2 according to current guidelines[26]: eGFR ≥60 = no known CKD; eGFR 45-59 = stage 3a; eGFR 30-44 = stage 3b; eGFR 15-29 = stage 4; eGFR <15 = stage 5. Baseline CKD status was derived from eGFR measurements in the year prior to start of follow-up by: 1) taking the last two measurements before the index date ≥90 days apart – with the higher eGFR value corresponding to the CKD baseline status, or 2) taking the most recent serum creatinine result if only one suitable test result was available. Since serum creatinine is more likely to be tested in the acutely unwell or in people who are routinely monitored as part of

For peer review only

incentivised programs (e.g. people with diabetes), patients without measurements of CKD baseline status were assumed to have no CKD[27] and were analysed as such.

Statistical Analysis

Though propensity score matching was employed to minimise confounding, we compared the distribution of baseline characteristics between the exposed and unexposed groups to check for any imbalances that may be relevant to the outcome of AKI. The baseline distribution of categorical variables was analysed using percentages and χ^2-tests. Continuous variables were analysed as means with standard deviations for normally distributed variables and medians with interquartile ranges for non-normally distributed variables. Differences in continuous variables were analysed with Student's t-tests or Wilcoxon rank sum tests for normally and non-normally distributed data, respectively.

The association between bariatric surgery and AKI was analysed using a Poisson regression model with a time to first event analysis. P-values were calculated using Wald tests. In order to separate short-term effects of the surgery from potential long-term effects, we analysed the association separately for: a) events within the first 30 days, and b) events after 30 days. When the cohort was initially constructed, propensity score matching was used to deal with confounding [7]. This study uses a subset of this cohort since patients from practices without linkage between the CPRD and HES databases had to be excluded (as AKI was assessed using hospital admission data). To identify variables for the multivariable model, potential confounders that were not deemed to be on the causal pathway were added individually to the univariable model. If the addition changed the effect estimate ≥10% these variables were included in the multivariable model. Consequently, history of AKI, history of taking oral

antidiabetics, and BMI at baseline were included (S2 Appendix). In addition, age at baseline, sex, calendar period (1997-2005, 2006-2010, 2011-2015), and CKD status at baseline were selected *a priori* as forced variables. For models with <40 outcomes, only age and sex were included in the multivariable model due to data sparsity.

The 5% bands of patients with the highest and lowest propensity scores were excluded from the primary analysis ("trimming") since these contain patients that are treated in stark contrast to their health status, potentially causing bias [28].

Heterogeneity of effect estimates between the calendar periods was tested with a Likelihood Ratio Test.

The analysis was performed for all patients with bariatric surgery and also further stratified by type of surgery. Patients with stage 5 CKD (baseline eGFR < 15 ml/min/1.73m^2) were excluded from the analyses since this constitutes end-stage renal failure (ESRD). In addition, patients with missing data in ≥1 variable of the multivariable model were excluded from both uni- and multivariable analyses.

All analyses were performed with Stata 14.1.

Subgroup analyses

Several planned sensitivity analyses were undertaken: 1) To determine the net effect of the intervention we calculated the risk of AKI over the whole period of follow-up; 2) The prevalence of decreased kidney function in the CPRD database was similar to that in a nationally representative kidney disease registry [27] indicating that patients with missing eGFR measurements are unlikely to have CKD. To identify potential differences in the effect between patients with known and unknown eGFR measurements, we restricted the analysis to a) patients known to have no CKD at baseline (baseline eGFR ≥ 60 ml/min/1.73m^2), b)

For peer review only

patients without known CKD at baseline (as above but including patients with missing creatinine values at baseline and assuming these individuals to have no CKD), and c) patients with known CKD at baseline.[27]; 3) Moreover, to investigate the effect in a group of particular interest which is under more scrutiny for measuring kidney function we restricted the analysis to patients with: a) T2DM, and b) a history of taking insulin; 4) To avoid misclassification of low eGFR values as AKI [29] we excluded patients with stage 4 CKD at baseline; 5) We restricted the analysis to ICD-10 codes N17.0 and N17.9, which have a high positive predictive value for AKI [24]; 6) We increased the immediate post-surgery time span from 30 to 60 days; 7) We included people with extreme propensity scores; and 8)We excluded patients with a BMI<35 kg/m² at baseline.

Ethical approval

This study was approved by the London School of Hygiene & Tropical Medicine ethics committee (LSHTM MSc Ethics Ref: 11065) and the Independent Scientific Advisory Committee on Medicines & Healthcare Products Regulatory Agency database research (approval number: 16_106R).

For peer review only

RESULTS

Since linkage to the HES-database was only possible for patients whose GPs had agreed for their practice data to be linked to HES (S3 Appendix), there were 2,643 patients with bariatric surgery and 2,595 people without surgery resulting in a cohort of overall 5,238 people with a median follow-up of 2.9 years (Table 1). The median follow-up prior to baseline was similar between the groups: 8.8 years (IQR: 8.1 years) for patients with bariatric surgery and 9.3 years (IQR: 8.0 years) for people without surgery.

For peer review only

Table 1: Baseline data for CPRD/HES-linked cohort study of people with bariatric surgery and the corresponding propensity score-matched comparison cohort (data are n (%) unless otherwise specified)*

	Bariatric Surgery (n = 2,643)	Matched Comparison group without surgery (n = 2,595)	p-value[1]
**Follow-up (years), median (IQR)**	2.9 (3.2)	2.9 (3.4)	0.616
**Age (years), mean (SD)**	45.2 (10.7)	45.0 (10.8)	0.417
**17 – 39, n (%)**	818 (31.0)	826 (31.8)	
**40 – 49, n (%)**	945 (35.8)	928 (35.8)	0.727
**50 – 85, n (%)**	880 (33.3)	841 (32.4)	
**BMI at baseline, mean (SD)**	44.9 (8.9)	42.2 (6.5)	<0.001
**13 – 34, n (%)**	297 (11.2)	287 (11.1)	
**35 – 39, n (%)**	448 (17.0)	456 (17.6)	
**40 – 44, n (%)**	625 (23.7)	1,118 (43.1)	<0.001
**45 – 49, n (%)**	571 (21.6)	438 (16.9)	
**50 – 94, n (%)**	667 (25.2)	253 (9.8)	
**Missing, n (%)**	35 (1.3)	43 (1.7)	
**Female**	2,131 (80.6)	2,131 (82.1)	0.166
**History of**			
**Cerebrovascular disease**	37 (1.4)	26 (1.0)	0.186
**Coronary heart disease**	104 (3.9)	82 (3.2)	0.130
**Peripheral vascular disease**	11 (0.4)	15 (0.6)	0.405
**Other atheroma**	0	<5[2]	0.313
**T2DM**	900 (34.1)	853 (32.9)	0.365
**Taking oral antidiabetic**	571 (21.6)	455 (17.5)	<0.001
**Taking insulin**	180 (6.8)	156 (6.0)	0.238
**Hypertension**	890 (33.7)	869 (33.5)	0.886
**Statin use**	699 (26.4)	640 (24.7)	0.139
**AKI**	30 (1.1)	11 (0.4)	0.003
**Alcohol status**			
**Non-drinker**	435 (16.5)	397 (15.3)	
**Ex-drinker**	278 (10.5)	236 (9.1)	
**Current drinker (amount unknown)**	15 (0.6)	13 (0.5)	
**<2 units/day**	659 (24.9)	644 (24.8)	0.366
**3-6 units/day**	862 (32.6)	909 (35.0)	
**>6 units/day**	170 (6.4)	164 (6.3)	
**Unknown**	224 (8.5)	232 (8.9)	
**Smoking status**			
**Non-smoker**	1,126 (42.6)	1,151 (44.4)	
**Current smoker**	403 (15.3)	345 (13.3)	0.093
**Ex-smoker**	1,112 (42.1)	1,099 (42.4)	
**Unknown**	<5[2]	0	
**CKD at baseline**			
**Baseline CKD status absent**	1,119 (42.3)	1,299 (50.1)	
**No CKD**	1,470 (55.6)	1,242 (47.9)	
**Stage 3a**	27 (1.0)	37 (1.4)	<0.001
**Stage 3b**	16 (0.6)	10 (0.4)	
**Stage 4**	10 (0.4)	5 (0.2)	
**Stage 5**	<5[2]	<5[2]	
**Type of bariatric surgery**			
**Gastric band**	1,193 (45.1)		
**Sleeve gastrectomy**	364 (13.8)		
**Gastric bypass**	1,075 (40.7)		
**Other**	11 (0.4)		
**ICD-10 code for AKI during follow-up**	**n = 44**	**n = 62**	
**N17.0 (Acute kidney failure with tubular necrosis)**	<5[2]	<5[2]	
**N17.9 (Acute kidney failure, unspecified)**	38 (86.4)	52 (83.9)	0.927
**N19 (Unspecified kidney failure)**	5 (11.4)	8 (12.9)	

[1] categorical variables: χ^2-test; continuous variables: t-test + SD if normally distributed, rank sum test + IQR if non-normally distributed
[2]cell counts <5 have been suppressed to ensure anonymity
*In the original study, each surgery patient was matched 1:1 to the person without surgery with the closest propensity score, choosing matches at random where more than one possible match had the same score [7]
AKI = acute kidney injury, BMI = body mass index, CKD = chronic kidney disease, ICD-10 = International Classification of Diseases, Tenth Revision, IQR = interquartile range, SD = standard deviation, T2DM = type 2 diabetes mellitus

For peer review only

238 This cohort was comparable to the cohort from the original study regarding sex, mean age,
239 mean BMI, history of T2DM, type of bariatric surgery and the imbalance of BMI at baseline
240 [7]. More patients in the intervention group had a history of AKI compared to the comparison
241 group (1.1% vs. 0.4%). Of the 106 included events during follow-up, 84.9% were classified
242 with the ICD-10 code N17.9 ("acute kidney failure, unspecified"), 12.3% were coded as N19
243 ("unspecified kidney failure"), and 2.8% had a code of N17.0 ("Acute kidney failure with
244 tubular necrosis"). CKD status at baseline was unknown for about half of the patients in
245 each group with a slightly higher proportion in the unexposed group (50.1% vs. 42.3%). The
246 majority of the patients with creatinine tests at baseline did not have CKD (96.2 %).
247 The number of AKI events recorded in the first 30 days of follow-up was low. All five events
248 happened in patients with bariatric surgery and none were recorded in the control group,
249 which is consistent with the possibility of an increased risk of AKI directly after surgery
250 (Table 2).

For peer review only

Table 2: Association of bariatric surgery with first incident AKI, stratified by length of follow-up. Unexposed refers to the propensity matched comparison group

	PY	Events	Rate per 1000 PY (95% CI)	Crude RR (95% CI)[1]	p-value[2]	Adjusted RR (95% CI)[3]	p-value[2]
**All patients**							
**Day 1-30**							
Unexposed	203	0	0	-			
Bariatric surgery	199	5	25.1 (10.5, 60.4)	-			
> **Day 30**							
Unexposed	7,882	54	6.9 (5.2, 8.9)	-			
Bariatric surgery	8,061	34	4.2 (3.0, 5.9)	0.62 (0.40, 0.95)	0.027	0.37 (0.23, 0.61)	<0.001
**All patients analysed by type of surgery[4]**							
**Day 1-30**							
Unexposed							
Gastric band							
Sleeve gastrectomy							
Gastric bypass							
Other							
> **Day 30**							
Unexposed	7,882	54	6.9 (5.2, 8.9)	-			
Gastric band	4,614	17	3.7 (2.3, 5.9)	0.54 (0.31, 0.93)	0.026		
Sleeve gastrectomy	728	<5[5]	5.5 (2.1, 14.6)	0.80 (0.29, 2.21)	0.670		
Gastric bypass	2,655	13	4.9 (2.8, 8.4)	0.71 (0.39, 1.31)	0.277		
Other	63	0	-	-			
**All patients over whole period of follow-up**							
Unexposed	8,085	54	6.7 (5.1, 8.7)	-			
Bariatric surgery	8,259	39	4.7 (3.5, 6.5)	0.71 (0.47, 1.07)	0.099	0.45 (0.28, 0.72)	0.001

[1] Poisson regression model
[2] Wald test
[3] Poisson regression model adjusted for age at baseline, sex, calendar time, CKD at baseline, history of AKI, history of taking oral antidiabetics, and BMI at baseline
[4] No analysis for day 1-30 owing to sparse data
[5] Cell counts <5 have been suppressed to ensure anonymity
AKI = acute kidney injury, CKD = chronic kidney disease, PY = person-years, RR = rate ratio

From 30 days onwards, bariatric surgery had a protective association with AKI risk (crude RR = 0.62, 95% CI 0.40, 0.95). The effect estimate of the multivariable model indicated an even stronger protective effect associated with bariatric surgery (RR = 0.37, 95% CI 0.23, 0.61), largely due to the confounding by AKI prior to baseline.

The analysis by type of surgery yielded protective effect estimates for all types but the confidence intervals were wide and no comparison between individual procedures was feasible. Sensitivity analyses yielded similar results (S4 Appendix). A sensitivity analysis restricted to patients with known CKD at baseline could not be done owing to sparse data.

Investigation of the effect of bariatric surgery over the whole follow-up period resulted in a protective net effect associated with the intervention in univariable (RR = 0.71, 95% CI 0.47, 1.07) and multivariable (RR = 0.45, 95% CI 0.28, 0.72) analyses.

For peer review only

DISCUSSION

In this study using prospectively recorded routine healthcare data from a representative sample in the UK, bariatric surgery was associated with a potentially increased risk of AKI within the first 30 days after surgery (5 events in patients with bariatric surgery, no events in control patients) but a strongly protective association thereafter (adjusted RR = 0.37, 95% CI 0.23, 0.61). The association was consistent across subgroups and sensitivity analyses. To the best of our knowledge, this is the first study to describe long-term effects of bariatric surgery on AKI.

AKI has been described as a perioperative event for bariatric surgery [12 13 15 16]. Our results are consistent with an increased risk in the early stages after surgery, however our analysis lacked enough early events to rule out chance as a reason for the results observed. Since patients do not have kidney function measures routinely checked by their family physician after bariatric surgery, many events could remain unnoticed. Patients with known CKD are more thoroughly checked for AKI and are a valuable subgroup to investigate, but the numbers in this dataset were too low to analyse.

This study uses high quality data from routine medical care in the UK. The healthcare system allows universal patient access to primary and secondary care so that the data is representative of the population. Patients are followed continuously while they are registered with a general practitioner allowing prospective data capture over long observation periods and avoiding problems with reverse causality. For the classification of AKI episodes in the HES database, the ICD-10 codes N17.0 and N17.9 comprised 87.7% of all events and have previously been shown to accurately identify AKI in a single centre study [24].

Some limitations need to be considered. Even though the data is taken from a representative sample of the UK population, the baseline data indicate that patients who

For peer review only

undergo bariatric surgery are mostly female, of middle age, and with a history of T2DM. While the results were adjusted for age and sex they might not be applicable for other groups suffering from obesity like adolescents. Linkage between the CPRD and HES databases was restricted to England. However, there is no cogent reason why the results should not be applicable to regions with similar healthcare systems, both in the UK and internationally. We had insufficient data to determine whether the association with AKI varied between different types of bariatric surgery; we found a protective effect for gastric band but results were inconclusive for sleeve gastrectomy and gastric bypass.

Misclassification of diagnostic codes is likely non-differential between the bariatric surgery patients and the matched comparison group and would bias the effect towards the null value. However, it is also conceivable during the immediate post-operative period those undergoing bariatric surgery might have been under more scrutiny to detect potential AKI events than people without surgery. In this case our current relative risk estimate for the immediate postoperative period would be an overestimate. Another problem of primary care data is that not every patient is routinely checked for their kidney function, as incentives of testing apply primarily for those at risk of kidney disease due to diabetes and hypertension. The study relied on AKI events recorded in HES as part of a hospital admission and over time, the awareness of the importance of AKI has likely changed resulting in secular changes in recording of AKI [30]; analyses have adjusted for calendar period to account for this [23 31]. Future studies with hospital creatinine data should compare the AKI severity between the groups to investigate this issue. In general, AKI diagnosed during hospitalisation is likely to represent more serious AKI events, though we would argue these are the most clinically relevant outcomes. Moreover, a patient who experienced a previous AKI episode might be under more scrutiny for detection of future episodes. Since more

For peer review only

patients in the bariatric surgery group had a history of AKI they might have a higher chance of detection of an AKI episode during follow-up, which we adjusted for in our analyses.

In addition, CKD status at baseline was missing in almost half of the patient population. However, a recent study indicated that the prevalence of CKD in the CPRD database was comparable to that found in nationally representative registry studies [27]. This indicates that patients without a GP record of eGFR-measurements at baseline are unlikely to have CKD. In addition, sensitivity analyses investigating the effect in patients with known or unknown CKD status at baseline yielded comparable results.

Since access to bariatric surgery is restricted within the UK healthcare system, some patients might have funded their operation privately, resulting in selection bias. In a recent analysis about 40% of bariatric surgery operations in the UK were privately funded [32]. Thus, the intervention group might have a higher socioeconomic status than the non-exposed group, in which similar patients would not be able to afford surgery. Since the socioeconomic background is an important determinant of health outcomes and was an unmeasured potential confounder not considered in the matching process, this could have led to more positive health outcomes in the intervention group irrespective of surgery and to an overestimation of the effect. In this study setting it was not possible to determine which patients had privately funded surgery.

Even though most baseline variables were evenly distributed due to the matching process this does not guarantee that unmeasured variables are evenly distributed as well, which can constitute residual confounding. Incorrect, imprecise, or missing measurements of covariates could also have led to residual confounding. For the multivariable model, adjusting for history of AKI led to the strongest change of the effect estimate. AKI events are likely under-recorded in the HES database, for reasons described above, and thus residual

For peer review only

337 confounding is possible. Since adjusting for AKI history led to a stronger effect estimate, the protective effect we report here may be an underestimate if AKI history is missing to the same degree in surgery and non-surgery patients.

This study adds to the evidence of long term effects of bariatric surgery, and appears to be the first study to quantify a long-term beneficial effect on AKI. Future studies with higher patient numbers may be able to determine differences in effect between types of surgery, investigate the effect in patients with CKD, and elucidate mechanisms of the association between bariatric surgery and AKI.

For peer review only

SUPPLEMENTARY INFORMATION

Supplementary information is available at the *BMJ open* website.

FUNDING

There was no specific funding to conduct this research project.

RM is supported by a Sir Henry Wellcome Postdoctoral Fellowship from the Wellcome Trust. KB holds a Sir Henry Dale fellowship jointly funded by the Wellcome Trust and the Royal Society. RLB is an NIHR Research Professor and supported by funding from the Rosetrees Trust and the Sir Jules Thorn Charitable Trust. LS is supported by a senior clinical fellowship from the Wellcome Trust. IJD is funded by an unrestricted grant from GlaxoSmithKline. None of the funders had any involvement in the design of the study, the data collection and analysis, the writing of the report, or the decision to submit the paper for publication.

CONFLICT OF INTEREST

The authors have no conflicts of interest to disclose.

DATA SHARING

The data were obtained from the Clinical Practice Research Datalink (CPRD). CPRD data governance does not allow us to distribute patient data to other parties. Researchers may apply for data access at www.CPRD.com. The codes used to produce the data for this study are provided in the Supporting Information.

For peer review only

CONTRIBUTIONS

UK, DN, RLB, IJD, and LS were responsible for conceptualisation of the study and formulate the research goals and aims. UK, DN, KEM, RM, KB, RLB, LS, and IJD developed the methodology and models. UK, KEM, KB, IJD, and RM worked on the data curation. UK performed the statistical analysis and wrote the original draft. UK, DN, KEM, RM, KB, RLB, LS, and IJD reviewed and commented the draft and gave input on editing.

For peer review only

REFERENCES

1. Public Health England. Obesity - UK and Ireland prevalence and trends. Available from: https://wwwnooorguk/NOO_about_obesity/adult_obesity/UK_prevalence_and_trends, accessed 25th August 2016.
2. Burton JO, Gray LJ, Webb DR, et al. Association of anthropometric obesity measures with chronic kidney disease risk in a non-diabetic patient population. Nephrol Dial Transplant 2012;**27**(5):1860-6.
3. Nguyen S, Hsu CY. Excess weight as a risk factor for kidney failure. Current Opinion in Nephrology and Hypertension 2007;**16**(2):71-76.
4. World Health Organisation. Obesity and overweight - Fact sheet. Available from: http://wwwwhoint/mediacentre/factsheets/fs311/en/, accessed 5th August 2016.
5. Colquitt JL, Pickett K, Loveman E, et al. Surgery for weight loss in adults. Cochrane Database Syst Rev 2014(8):CD003641.
6. Gloy VL, Briel M, Bhatt DL, et al. Bariatric surgery versus non-surgical treatment for obesity: a systematic review and meta-analysis of randomised controlled trials. BMJ 2013;**347**:f5934.
7. Douglas IJ, Bhaskaran K, Batterham RL, et al. Bariatric Surgery in the United Kingdom: A Cohort Study of Weight Loss and Clinical Outcomes in Routine Clinical Care. PLoS Med 2015;**12**(12):e1001925.
8. Lewington AK, S. Clinical Practice Guidelines - Acute Kidney Injury. UK Renal Association 2011.
9. Shashaty MG, Meyer NJ, Localio AR, et al. African American race, obesity, and blood product transfusion are risk factors for acute kidney injury in critically ill trauma patients. J Crit Care 2012;**27**(5):496-504.
10. Suneja M, Kumar AB. Obesity and perioperative acute kidney injury: A focused review. Journal of Critical Care 2014;**29**(4).
11. Currie A, Chetwood A, Ahmed AR. Bariatric surgery and renal function. Obesity Surgery 2011;**21**(4):528-39.
12. Thakar CV, Kharat V, Blanck S, et al. Acute kidney injury after gastric bypass surgery. Clin J Am Soc Nephrol 2007;**2**(3):426-30.
13. Weingarten TN, Gurrieri C, McCaffrey JM, et al. Acute kidney injury following bariatric surgery. Obesity Surgery 2013;**23**(1):64-70.
14. Chakravartty S, Sarma DR, Patel AG. Rhabdomyolysis in bariatric surgery: A Systematic review. Obesity Surgery 2013;**23**(8):1333-40.
15. Abdullah HR, Tan TP, Vaez M, et al. Predictors of Perioperative Acute Kidney Injury in Obese Patients Undergoing Laparoscopic Bariatric Surgery: a Single-Centre Retrospective Cohort Study. Obes Surg 2016;**26**(7):1493-9.
16. Sharma SK, McCauley J, Cottam D, et al. Acute changes in renal function after laparoscopic gastric surgery for morbid obesity. Surg Obes Relat Dis 2006;**2**(3):389-92.
17. Bhatti UH, Duffy AJ, Roberts KE, et al. Nephrolithiasis after bariatric surgery: A review of pathophysiologic mechanisms and procedural risk. Int J Surg 2016;**36**(Pt D):618-23.
18. Chisholm J. The Read clinical classification. BMJ 1990;**300**(6732):1092.
19. Williams T, van Staa T, Puri S, et al. Recent advances in the utility and use of the General Practice Research Database as an example of a UK Primary Care Data resource. Ther Adv Drug Saf 2012;**3**(2):89-99.

For peer review only

20. Herrett E, Gallagher AM, Bhaskaran K, et al. Data Resource Profile: Clinical Practice Research Datalink (CPRD). Int J Epidemiol 2015;**44**(3):827-36.
21. Herrett E, Thomas SL, Schoonen WM, et al. Validation and validity of diagnoses in the General Practice Research Database: a systematic review. Br J Clin Pharmacol 2010;**69**(1):4-14.
22. National Health Service. The Information Centre. Hospital Episode Statistics. Available from: http://digitalnhsuk/hes, accessed 12th August 2016.
23. McDonald HI, Shaw C, Thomas SL, et al. Methodological challenges when carrying out research on CKD and AKI using routine electronic health records. Kidney Int 2016.
24. Tomlinson LA, Riding AM, Payne RA, et al. The accuracy of diagnostic coding for acute kidney injury in England - a single centre study. BMC Nephrol 2013;**14**:58.
25. Levey AS, Stevens LA, Schmid CH, et al. A new equation to estimate glomerular filtration rate. Ann Intern Med 2009;**150**(9):604-12.
26. National Institute for Health and Care Excellence. Chronic kidney disease in adults: assessment and management, Clinical guideline. 2014.
27. Iwagami M, Tomlinson LA, Mansfield KE, et al. Validity of estimated prevalence of decreased kidney function and renal replacement therapy from primary care electronic health records compared with national survey and registry data in the United Kingdom. Nephrol Dial Transplant 2017.
28. Sturmer T, Rothman KJ, Avorn J, et al. Treatment effects in the presence of unmeasured confounding: dealing with observations in the tails of the propensity score distribution--a simulation study. Am J Epidemiol 2010;**172**(7):843-54.
29. Hill RS. Acute Kidney Injury Warning Algorithm - Best Practice Guidance. Secondary Acute Kidney Injury Warning Algorithm - Best Practice Guidance 2014. https://www.thinkkidneys.nhs.uk/wp-content/uploads/2014/12/AKI-Warning-Algorithm-Best-Practice-Guidance-final-publication-0112141.pdf.
30. McDonald HI, Shaw C, Thomas SL, et al. Methodological challenges when carrying out research on CKD and AKI using routine electronic health records. Kidney Int 2016;**90**(5):943-49.
31. James M, Pannu N. Methodological considerations for observational studies of acute kidney injury using existing data sources. J Nephrol 2009;**22**(3):295-305.
32. Gulliford MC, Charlton J, Booth HP, et al. Costs and outcomes of increasing access to bariatric surgery for obesity: cohort study and cost-effectiveness analysis using electronic health records. Southampton (UK), 2016.

For peer review only

Long-term effects of bariatric surgery on acute kidney injury: A propensity-matched cohort in the United Kingdom Clinical Practice Research Datalink

Supporting Information

Overview

S1 Appendix – Code List for Identification of patients with bariatric surgery

S2 Appendix – Association of potential confounders with bariatric surgery and AKI

S3 Appendix – Patient selection from the original cohort

S4 Appendix – Sensitivity Analyses

For peer review only

S1 Appendix

Appendix 1: Code List for identification of patients with bariatric surgery from the CPRD database as published by Douglas et al. [7]

Read code	*description*
76132.00	Laparoscopic adjustable gastric banding
76134.00	Partitioning of stomach using staples
76131.11	Mason vertical banded gastroplasty
76133.00	Partitioning of stomach using band
76116.00	Laparoscopic sleeve gastrectomy
76115.00	Sleeve gastrectomy NEC
76425.00	Duodenal switch
76135.00	Partitioning of stomach NEC
76114.00	Sleeve gastrectomy and duodenal switch
76166.00	Laparoscopic gastric bypass

For peer review only

S2 Appendix

Appendix 2: Identification of potential confounders in the association of bariatric surgery (exposure) and the endpoint of incident AKI (outcome) in patients of the linked CPRD/HES cohort

	RR (95%CI)	Change in %	Selection for multivariable model
**Crude effect estimate**	0.62 (0.40, 0.95)		
**Effect estimates when individually adjusting for**			
**Age**	0.62 (0.40, 0.95)	0.2 %	yes (a priori)
**Sex**	0.60 (0.39, 0.92)	2.7 %	yes (a priori)
**Calendar Time**	0.61 (0.40, 0.94)	0.9%	yes (a priori)
**CKD status at baseline**	0.59 (0.38, 0.91)	4.4 %	yes (a priori)
**BMI at baseline**	0.53 (0.34, 0.83)	13.9 %	yes
**Alcohol Status**	0.61 (0.40, 0.93)	1.3 %	no
**Smoking Status**	0.61 (0.40, 0.94)	0.3 %	no
**History of cerebrovascular disease**	0.61 (0.40, 0.94)	0.6 %	no
**History of coronary heart disease**	0.60 (0.39, 0.91)	3.3 %	no
**History of peripheral vascular disease**	0.64 (0.41, 0.98)	3.2 %	no
**History of other atheroma**	0.62 (0.40, 0.95)	0.0 %	no
**History of diabetes**	0.60 (0.39, 0.92)	2.7%	no
**History of taking oral antidiabetics**	0.55 (0.36, 0.85)	10.4%	yes
**History of taking insulin**	0.57 (0.37, 0.87)	7.9 %	no
**History of hypertension**	0.61 (0.40, 0.94)	1.1 %	no
**History of statin use**	0.58 (0.38, 0.89)	5.5 %	no
**History of AKI**	0.42 (0.26, 0.67)	31.9 %	yes

Variables were added individually to the univariable model testing the association between bariatric surgery and AKI. If the addition of the respective variable changed the model ≥10% then the variable was selected to be included in the multivariable model.
AKI = acute kidney injury, BMI = body mass index, CKD = chronic kidney disease

For peer review only

S3 Appendix

Appendix 3: Patient selection from the original cohort as described in Douglas et al [7]

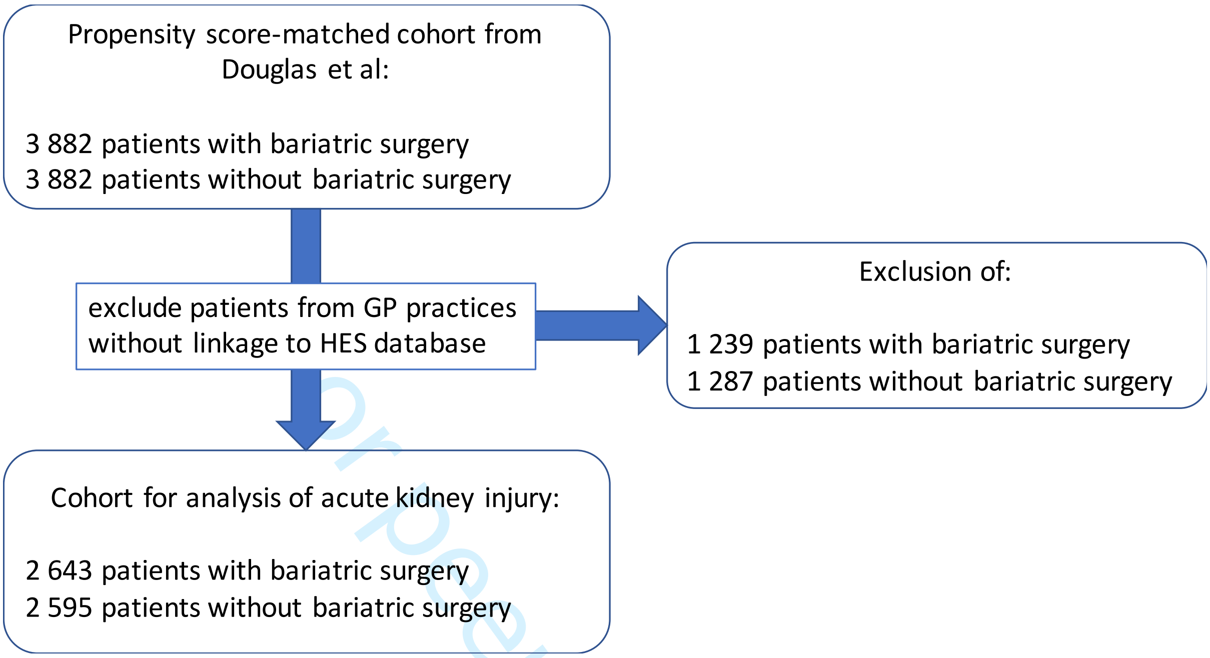

For peer review only

S4 Appendix

Appendix 4: Sensitivity analyses for the association of bariatric surgery with acute kidney injury

	PY	Events	Rate per 1000 PY (95% CI)	Crude RR (95% CI)[1]	p-value[2]	Adjusted RR (95% CI)[3]	p-value[2]
**Restricted to patients without CKD at baseline (available serum creatinine measures + eGFR ≥60)**							
**Day 1-30**							
Unexposed	98	0	0	-			
Bariatric surgery	111	<5[6]	36.2 (13.6, 96.3)	-			
**>Day 30**							
Unexposed	3,550	27	7.6 (5.2, 11.1)	-			
Bariatric surgery	4,311	22	5.1 (3.4, 7.7)	0.67 (0.38, 1.18)	0.165	0.53 (0.29, 1.00)	0.050
**Restricted to patients without known CKD at baseline (available serum creatinine measures + eGFR ≥60 or missing eGFR at baseline)**							
**Day 1-30**							
Unexposed	199	0	0	-			
Bariatric surgery	195	<5[6]	20.5 (7.7, 54.7)	-			
**>Day 30**							
Unexposed	7,735	42	5.4 (4.0, 7.3)	-			
Bariatric surgery	7,930	27	3.4 (2.3, 5.0)	0.63 (0.39, 1.02)	0.058	0.42 (0.25, 0.73)	0.002
**Excluding patients with CKD stage 4**							
**Day 1-30**							
Unexposed	203	0	0	-			
Bariatric surgery	198	5	25.2 (10.5, 60.6)	-			
**> Day 30**							
Unexposed	7,875	52	6.6 (5.0, 8.7)	-			
Bariatric surgery	8,037	32	4.0 (2.8, 5.6)	0.60 (0.39, 0.94)	0.024	0.35 (0.21, 0.59)	<0.001
**Restricted to patients with T2DM**							
**Day 1-30**							
Unexposed	65	0	0	-			
Bariatric surgery	69	<5[6]	43.6 (14.1, 135.1)	-			
**>Day 30**							
Unexposed	2,325	33	14.2 (10.1, 20.0)	-			
Bariatric surgery	2,548	18	7.1 (4.5, 11.2)	0.50 (0.28, 0.88)	0.017	0.25 (0.13, 0.51)	<0.001
**Restricted to patients with a history of taking insulin**							
**Day 1-30**							
Unexposed	11	0	0	-			
Bariatric surgery	13	0	0	-			
**>Day 30**							
Unexposed	321	11	34.3 (19.0, 61.9)	-			
Bariatric surgery	502	9	17.9 (9.3, 34.5)	0.52 (0.22, 1.26)	0.150	0.22 (0.08, 0.64)	0.005
**Restricted to ICD-10 codes N17.0 and N17.9**							
**Day 1-30**							
Unexposed	202	0	0	-			
Bariatric surgery	199	5	25.2 (10.5, 60.5)	-			
**>Day 30**							
Unexposed	7,871	48	6.1 (4.6, 8.1)	-			
Bariatric surgery	8,055	31	3.8 (2.7, 5.5)	0.63 (0.40, 0.99)	0.046	0.40 (0.24, 0.67)	<0.001
**Having an initial post-surgery time span of 60 days instead of 30**							
**Day 1-60**							
Unexposed	403	<5[6]	2.5 (0.3, 17.6)	-			
Bariatric surgery	395	6	15.2 (6.8, 33.8)	6.11 (0.74, 50.8)	0.094	[4]	
**> Day 60**							
Unexposed	7,682	53	6.9 (5.3, 9.0)	-			
Bariatric surgery	7,864	33	4.2 (3.0, 5.9)	0.61 (0.39, 0.94)	0.025	0.38 (0.23, 0.63)	<0.001
				Test for interaction[5]	0.011		

**Including patients with extreme propensity scores**							
**Day 1-30**							
Unexposed	208	0	0	-			
Bariatric surgery	206	5	24.3 (10.1, 58.3)	-			
> **Day 30**							
Unexposed	8,054	59	7.3 (5.7, 9.5)	-			
Bariatric surgery	8,324	34	4.1 (2.9, 5.7)	0.56 (0.37, 0.85)	0.007	0.33 (0.20, 0.54)	<0.001
**Excluding patients with BMI < 35 kg/m² at baseline**							
**Day 1-30**							
Unexposed	180	0	0	-			
Bariatric surgery	175	<5[6]	22.8 (8.6, 60.9)	-			
> **Day 30**							
Unexposed	6,714	48	7.1 (5.4, 9.5)	-			
Bariatric surgery	7,100	32	4.5 (3.2, 6.4)	0.63 (0.40, 0.99)	0.043	0.39 (0.23, 0.65)	<0.001

[1] Poisson regression model
[2] Wald test for RR, Likelihood-Ratio Test for interaction
[3] Poisson regression model adjusted for age at baseline, sex, calendar time, CKD at baseline, history of AKI, history of taking oral antidiabetics, and BMI at baseline
[4] No analysis for day 1-30 owing to sparse data
[5] Test for interaction of the effect estimate with the time periods 1-30 days and >30 days
[6] cell counts <5 have been suppressed to ensure anonymity
AKI = acute kidney injury, CKD = chronic kidney disease, PY = person-years, RR = rate ratio

For peer review only

Supporting Information:
STROBE statement checklist to ensure appropriate reporting of study information of long-term effects of acute kidney injury for the propensity-matched cohort study of patients with and without bariatric surgery

	Item No	Report
**Title and abstract**	1	(*a*) Indicate the study's design with a commonly used term in the title or the abstract<br><br>"Long-term effects of bariatric surgery on acute kidney injury: A propensity-matched cohort in the United Kingdom Clinical Practice Research Datalink"
		b) Provide in the abstract an informative and balanced summary of what was done and what was found<br><br>Abstract: on page 2 containing Background, Methods, Results and Conclusions
**Introduction**		
Background/rationale	2	Explain the scientific background and rationale for the investigation being reported<br><br>See page 4 for description of background;<br>Rationale (p4): "To the best of our knowledge, no studies have been published examining the long-term effects of bariatric surgery on AKI."
Objectives	3	State specific objectives, including any prespecified hypotheses<br><br>Page 4: "In this study, we investigate the long-term effects of bariatric surgery on AKI to see whether the expected reduction in BMI has any impact on subsequent renal health."
**Methods**		
Study design	4	Present key elements of study design early in the paper<br><br>See page 5: "We undertook a matched cohort study using prospectively collected data from CPRD patients registered before 31st December 2014 linked to the Hospital Episodes Statistics (HES) database to investigate long-term effects of bariatric surgery on AKI."
Setting	5	Describe the setting, locations, and relevant dates, including periods of recruitment, exposure, follow-up, and data collection<br><br>Page 5: "The CPRD database contains anonymised, routinely collected data on approximately 10 million patients in participating primary care practices in the UK, including demographic characteristics, current and previous diagnoses, prescribing, test results, and lifestyle factors. […].The HES database contains patient data from hospital admissions to English hospitals within the National Health Service […].Data from 70% of CPRD practices in England has been linked at patient level with HES admission data thus allowing the combined analysis of data from primary and acute hospital care for a subset of patients."<br>"A detailed description of how the cohort was constructed is described elsewhere. In brief, records of patients who underwent bariatric surgery (n=3,882) between 1997 and 2015 were matched to individuals who did not undergo surgery (n=3,882) using propensity scores."<br>Page 6: "Follow-up started on the day of surgery for those with bariatric surgery, and for the comparison group who did not undergo bariatric surgery, on the surgery date

For peer review only - http://bmjopen.bmj.com/site/about/guidelines.xhtml

		of their matched case. Patient records were censored at the earliest of: AKI, death, leaving the practice, latest data collection from current practice, or end of linkage period to the HES database."
Participants	6	(*a*) Give the eligibility criteria, and the sources and methods of selection of participants. Describe methods of follow-up<br><br>Page 6: "Patients with bariatric surgery were identified using Read codes for surgery in the CPRD database (S1 Appendix) and were included in the study if they had been registered in the CPRD ≥12 months prior to the intervention. We excluded those with a record of prior bariatric surgery reversal.<br>For the comparison group, the inclusion criteria were to have at least one BMI measurement ≥40 kg/m2, ≥12 months of follow-up prior to the index date in the database, and no prior record of bariatric surgery or bariatric surgery reversal."<br><br>Page 5: "The CPRD database contains anonymised, routinely collected data on approximately 10 million patients in participating primary care practices in the UK, including demographic characteristics, current and previous diagnoses, prescribing, test results, and lifestyle factors. Diagnoses, signs, and symptoms are recorded using Read codes […]. The HES database contains patient data from hospital admissions to English hospitals within the National Health Service "
		(*b*) For matched studies, give matching criteria and number of exposed and unexposed<br><br>Page 6: "Study population matching and the propensity score incorporated information on age, sex, calendar period, history of T2DM, hypertension, coronary heart disease, cerebrovascular disease, peripheral vascular disease, other atheroma, use of insulin, use of oral antidiabetic medication, use of statins, smoking status, and alcohol consumption."<br>"The study sample was restricted to eligible patients registered at practices linked to the HES database and information on AKI events was obtained, resulting in a final cohort comprising 2,643 patients who underwent bariatric surgery, and 2,595 patients who did not."
Variables	7	Clearly define all outcomes, exposures, predictors, potential confounders, and effect modifiers. Give diagnostic criteria, if applicable<br><br>Page 6/7: "AKI episodes were obtained from the HES database using ICD-10 codes: N17.0 ("Acute kidney failure with tubular necrosis"), N17.1 ("Acute renal failure with acute cortical necrosis), N17.2 ("Acute renal failure with medullary necrosis"), N17.8 ("Other acute renal failure"), N17.9 ("Acute kidney failure, unspecified"), and N19 ("Unspecified kidney failure"). In this cohort, events coded with N17.1, N17.2, and N17.8 were not found. AKI events that occurred before the start of follow-up were recorded as a binary variable "history of AKI", while AKI events occurring during follow-up were used to analyse AKI incidence.<br>Recorded serum creatinine values from the CPRD database were not routinely standardised with isotope-dilution mass spectrometry before 2013. Thus, we assumed all measurements to be unstandardized and multiplied the creatinine measures with the factor 0.95 before calculating the estimated glomerular filtration rate (eGFR) using the "Chronic Kidney Disease Epidemiology Collaboration" (CKD-EPI) equation.

For peer review only - http://bmjopen.bmj.com/site/about/guidelines.xhtml
For peer review only

		Ethnicity was not considered in the eGFR calculation due to incomplete recording in the database and the low proportion of Afro-Caribbean people in the population. CKD stages were defined according to eGFR values in ml/min/1.73m^2 according to current guidelines: eGFR ≥60 = no known CKD; eGFR 45-59 = stage 3a; eGFR 30-44 = stage 3b; eGFR 15-29 = stage 4; eGFR <15 = stage 5. Baseline CKD status was derived from eGFR measurements in the year prior to start of follow-up by: 1) taking the last two measurements before the index date ≥90 days apart – with the higher eGFR value corresponding to the CKD baseline status, or 2) taking the most recent serum creatinine result if only one suitable test result was available. Since serum creatinine is more likely to be tested in the acutely unwell or in people who are routinely monitored as part of incentivised programs (e.g. people with diabetes), patients without measurements of CKD baseline status were assumed to have no CKD and were analysed as such."<br>Page 6: "Study population matching and the propensity score incorporated information on age, sex, calendar period, history of T2DM, hypertension, coronary heart disease, cerebrovascular disease, peripheral vascular disease, other atheroma, use of insulin, use of oral antidiabetic medication, use of statins, smoking status, and alcohol consumption."<br>Page 8: "When the cohort was initially constructed, propensity score matching was used to deal with confounding. This study uses a subset of this cohort since patients from practices without linkage between the CPRD and HES databases had to be excluded (as AKI was assessed using hospital admission data). To identify variables for the multivariable model, potential confounders that were not deemed to be on the causal pathway were added individually to the univariable model. If the addition changed the effect estimate ≥10% these variables were included in the multivariable model. Consequently, history of AKI, history of taking oral antidiabetics, and BMI at baseline were included (S2 Appendix). In addition, age at baseline, sex, calendar period (1997-2005, 2006-2010, 2011-2015), and CKD status at baseline were selected a priori as forced variables."<br>Page 8: "In order to separate short-term effects of the surgery from potential long-term effects, we analysed the association separately for: a) events within the first 30 days, and b) events after 30 days."
Data sources/ measurement	8*	For each variable of interest, give sources of data and details of methods of assessment (measurement). Describe comparability of assessment methods if there is more than one group<br><br>Page 5: "The CPRD database contains anonymised, routinely collected data on approximately 10 million patients in participating primary care practices in the UK, including demographic characteristics, current and previous diagnoses, prescribing, test results, and lifestyle factors. Diagnoses, signs, and symptoms are recorded using Read codes […]The HES database contains patient data from hospital admissions to English hospitals within the National Health Service. For each hospital admission, the diagnoses are recorded using standardised codes of the International Classification of Diseases, Tenth Revision (ICD-10). Data from 70% of CPRD practices in England has been linked at patient level with HES admission data thus allowing the combined analysis of data from primary and acute hospital care for a subset of patients."<br>Page 5: "Patients with bariatric surgery were identified using Read codes for surgery in the CPRD database (S1 Appendix) and were included in the study if they had been

For peer review only

		registered in the CPRD ≥12 months prior to the intervention. We excluded those with a record of prior bariatric surgery reversal."<br>Page 6/7: "AKI episodes were obtained from the HES database using ICD-10 codes: N17.0 ("Acute kidney failure with tubular necrosis"), N17.1 ("Acute renal failure with acute cortical necrosis), N17.2 ("Acute renal failure with medullary necrosis"), N17.8 ("Other acute renal failure"), N17.9 ("Acute kidney failure, unspecified"), and N19 ("Unspecified kidney failure")."
Bias	9	Describe any efforts to address potential sources of bias<br><br>Page 9: "The 5% bands of patients with the highest and lowest propensity scores were excluded from the primary analysis ("trimming") since these contain patients that are treated in stark contrast to their health status, potentially causing bias."
Study size	10	Explain how the study size was arrived at<br><br>Pages 5/6: "A detailed description of how the cohort was constructed is described elsewhere. In brief, records of patients who underwent bariatric surgery (n=3,882) between 1997 and 2015 were matched to individuals who did not undergo surgery (n=3,882) using propensity scores." […] "The study sample was restricted to eligible patients registered at practices linked to the HES database and information on AKI events was obtained, resulting in a final cohort comprising 2,643 patients who underwent bariatric surgery, and 2,595 patients who did not."
Quantitative variables	11	Explain how quantitative variables were handled in the analyses. If applicable, describe which groupings were chosen and why<br><br>Page 7: "Recorded serum creatinine values from the CPRD database were not routinely standardised with isotope-dilution mass spectrometry before 2013. Thus, we assumed all measurements to be unstandardized and multiplied the creatinine measures with the factor 0.95 before calculating the estimated glomerular filtration rate (eGFR) using the "Chronic Kidney Disease Epidemiology Collaboration" (CKD-EPI) equation. Ethnicity was not considered in the eGFR calculation due to incomplete recording in the database and the low proportion of Afro-Caribbean people in the population. CKD stages were defined according to eGFR values in ml/min/1.73m2 according to current guidelines: eGFR ≥60 = no known CKD; eGFR 45-59 = stage 3a; eGFR 30-44 = stage 3b; eGFR 15-29 = stage 4; eGFR <15 = stage 5. Baseline CKD status was derived from eGFR measurements in the year prior to start of follow-up by: 1) taking the last two measurements before the index date ≥90 days apart – with the higher eGFR value corresponding to the CKD baseline status, or 2) taking the most recent serum creatinine result if only one suitable test result was available. Since serum creatinine is more likely to be tested in the acutely unwell or in people who are routinely monitored as part of incentivised programs (e.g. people with diabetes), patients without measurements of CKD baseline status were assumed to have no CKD and were analysed as such."<br>Page 8: "In addition, age at baseline, sex, calendar period (1997-2005, 2006-2010, 2011-2015), and CKD status at baseline were selected a priori as forced variables."
Statistical methods	12	(*a*) Describe all statistical methods, including those used to control for confounding<br><br>Page 8/9: "The association between bariatric surgery and AKI was analysed using a Poisson regression model with a time to first event analysis. P-values were calculated

For peer review only - http://bmjopen.bmj.com/site/about/guidelines.xhtml

using Wald tests. In order to separate short-term effects of the surgery from potential long-term effects, we analysed the association separately for: a) events within the first 30 days, and b) events after 30 days. When the cohort was initially constructed, propensity score matching was used to deal with confounding. This study uses a subset of this cohort since patients from practices without linkage between the CPRD and HES databases had to be excluded (as AKI was assessed using hospital admission data). To identify variables for the multivariable model, potential confounders that were not deemed to be on the causal pathway were added individually to the univariable model. If the addition changed the effect estimate ≥10% these variables were included in the multivariable model. Consequently, history of AKI, history of taking oral antidiabetics, and BMI at baseline were included (S2 Appendix). In addition, age at baseline, sex, calendar period (1997-2005, 2006-2010, 2011-2015), and CKD status at baseline were selected a priori as forced variables. For models with <40 outcomes, only age and sex were included in the multivariable model due to data sparsity.

The 5% bands of patients with the highest and lowest propensity scores were excluded from the primary analysis ("trimming") since these contain patients that are treated in stark contrast to their health status, potentially causing bias.

Heterogeneity of effect estimates between the calendar periods was tested with a Likelihood Ratio Test.

The analysis was performed for all patients with bariatric surgery and also further stratified by type of surgery. Patients with stage 5 CKD (baseline eGFR < 15 ml/min/1.73m2) were excluded from the analyses since this constitutes end-stage renal failure (ESRD). In addition, patients with missing data in ≥1 variable of the multivariable model were excluded from both uni- and multivariable analyses."

(*b*) Describe any methods used to examine subgroups and interactions

page 8: "In order to separate short-term effects of the surgery from potential long-term effects, we analysed the association separately for: a) events within the first 30 days, and b) events after 30 days."

page 9: "Heterogeneity of effect estimates between the calendar periods was tested with a Likelihood Ratio Test."

Page 9: "The analysis was performed for all patients with bariatric surgery and also further stratified by type of surgery."

(*c*) Explain how missing data were addressed

page 7: "Since serum creatinine is more likely to be tested in the acutely unwell or in people who are routinely monitored as part of incentivised programs (e.g. people with diabetes), patients without measurements of CKD baseline status were assumed to have no CKD and were analysed as such."

page 9: "In addition, patients with missing data in ≥1 variable of the multivariable model were excluded from both uni- and multivariable analyses."

(*d*) If applicable, explain how loss to follow-up was addressed

page 6: "Patient records were censored at the earliest of: AKI, death, leaving the practice, latest data collection from current practice, or end of linkage period to the

For peer review only - http://bmjopen.bmj.com/site/about/guidelines.xhtml

		HES database."
		(*e*) Describe any sensitivity analyses<br><br>Pages 9/10: "Several planned sensitivity analyses were undertaken: 1) To determine the net effect of the intervention we calculated the risk of AKI over the whole period of follow-up; 2) The prevalence of decreased kidney function in the CPRD database was similar to that in a nationally representative kidney disease registry indicating that patients with missing eGFR measurements are unlikely to have CKD. To identify potential differences in the effect between patients with known and unknown eGFR measurements, we restricted the analysis to a) patients known to have no CKD at baseline (baseline eGFR ≥ 60 ml/min/1.73m^2), b) patients without known CKD at baseline (as above but including patients with missing creatinine values at baseline and assuming these individuals to have no CKD), and c) patients with known CKD at baseline.; 3) Moreover, to investigate the effect in a group of particular interest which is under more scrutiny for measuring kidney function we restricted the analysis to patients with: a) T2DM, and b) a history of taking insulin; 4) To avoid misclassification of low eGFR values as AKI we excluded patients with stage 4 CKD at baseline; 5) We restricted the analysis to ICD-10 codes N17.0 and N17.9, which have a high positive predictive value for AKI; 6) We increased the immediate post-surgery time span from 30 to 60 days; and 7) We included people with extreme propensity scores."
**Results**		
Participants	13*	(a) Report numbers of individuals at each stage of study—eg numbers potentially eligible, examined for eligibility, confirmed eligible, included in the study, completing follow-up, and analysed<br><br>see S3 Appendix
		(b) Give reasons for non-participation at each stage<br><br>not applicable
		(c) Consider use of a flow diagram<br><br>see S3 Appendix
Descriptive data	14*	(a) Give characteristics of study participants (eg demographic, clinical, social) and information on exposures and potential confounders<br><br>page 12: see Table 1
		(b) Indicate number of participants with missing data for each variable of interest<br><br>page 12: see Table 1
		(c) Summarise follow-up time (eg, average and total amount)<br><br>page 12: see Table 1
Outcome data	15*	Report numbers of outcome events or summary measures over time<br><br>page 12: see Table 1
Main results	16	(*a*) Give unadjusted estimates and, if applicable, confounder-adjusted estimates and their precision (eg, 95% confidence interval). Make clear which confounders were

For peer review only - http://bmjopen.bmj.com/site/about/guidelines.xhtml

		adjusted for and why they were included<br><br>page 13: see Table 2
		(*b*) Report category boundaries when continuous variables were categorized<br><br>page 12: see Table 1
		(*c*) If relevant, consider translating estimates of relative risk into absolute risk for a meaningful time period<br><br>not applicable
Other analyses	17	Report other analyses done—eg analyses of subgroups and interactions, and sensitivity analyses<br><br>Page 13: see table 2<br>see S4 Appendix
**Discussion**		
Key results	18	Summarise key results with reference to study objectives<br><br>Page 15: "In this study using prospectively recorded routine healthcare data from a representative sample in the UK, bariatric surgery was associated with a potentially increased risk of AKI within the first 30 days after surgery (5 events in patients with bariatric surgery, no events in control patients) but a strongly protective association thereafter (adjusted RR = 0.37, 95% CI 0.23, 0.61). The association was consistent across subgroups and sensitivity analyses. To the best of our knowledge, this is the first study to describe long-term effects of bariatric surgery on AKI."
Limitations	19	Discuss limitations of the study, taking into account sources of potential bias or imprecision. Discuss both direction and magnitude of any potential bias<br><br>Pages 15-18: "Some limitations need to be considered. Even though the data is taken from a representative sample of the UK population, the baseline data indicate that patients who undergo bariatric surgery are mostly female, of middle age, and with a history of T2DM. While the results were adjusted for age and sex they might not be applicable for other groups suffering from obesity like adolescents. Linkage between the CPRD and HES databases was restricted to England. However, there is no cogent reason why the results should not be applicable to regions with similar healthcare systems, both in the UK and internationally. We had insufficient data to determine whether the association with AKI varied between different types of bariatric surgery; we found a protective effect for gastric band but results were inconclusive for sleeve gastrectomy and gastric bypass.<br>Any misclassification of diagnostic codes is likely non-differential between the bariatric surgery patients and the matched comparison group and would bias the effect towards the null value. Another problem of primary care data is that not every patient is routinely checked for their kidney function, as incentives of testing apply primarily for those at risk of kidney disease due to diabetes and hypertension. The study relied on AKI events recorded in HES as part of a hospital admission and over time, the awareness of the importance of AKI has likely changed resulting in secular changes in recording of AKI ; analyses have adjusted for calendar period to account for this. Future studies with hospital creatinine data should compare the AKI severity between the groups to investigate this issue. In general, AKI diagnosed during hospitalisation

For peer review only

		is likely to represent more serious AKI events, though we would argue these are the most clinically relevant outcomes. Moreover, a patient who experienced a previous AKI episode might be under more scrutiny for detection of future episodes. Since more patients in the bariatric surgery group had a history of AKI they might have a higher chance of detection of an AKI episode during follow-up. This would bias the estimate towards the null value and could indicate that the association we report is an under-estimate.<br>In addition, CKD status at baseline was missing in almost half of the patient population. However, a recent study indicated that the prevalence of CKD in the CPRD database was comparable to that found in nationally representative registry studies. This indicates that patients without eGFR-measurements at baseline are unlikely to have CKD. In addition, sensitivity analyses investigating the effect in patients with known or unknown CKD status at baseline yielded comparable results.<br>Since access to bariatric surgery is restricted within the UK healthcare system, some patients might have funded their operation privately, resulting in selection bias. In a recent analysis about 40% of bariatric surgery operations in the UK were privately funded. Thus, the intervention group might have a higher socioeconomic status than the non-exposed group, in which similar patients would not be able to afford surgery. Since the socioeconomic background is an important determinant of health outcomes and was an unmeasured potential confounder not considered in the matching process, this could have led to more positive health outcomes in the intervention group irrespective of surgery and to an overestimation of the effect. In this study setting it was not possible to determine which patients had privately funded surgery.<br>Even though most baseline variables were evenly distributed due to the matching process this does not guarantee that unmeasured variables are evenly distributed as well, which can constitute residual confounding. Incorrect, imprecise, or missing measurements of covariates could also have led to residual confounding. For the multivariable model, adjusting for history of AKI led to the strongest change of the effect estimate. AKI events are likely under-recorded in the HES database, for reasons described above, and thus residual confounding is possible. Since adjusting for AKI history led to a stronger effect estimate, the protective effect we report here may be an underestimate if AKI history is missing to the same degree in surgery and non-surgery patients."
Interpretation	20	Give a cautious overall interpretation of results considering objectives, limitations, multiplicity of analyses, results from similar studies, and other relevant evidence<br><br>Page 18: "This study adds to the evidence of long term effects of bariatric surgery, and appears to be the first study to quantify a long-term beneficial effect on AKI. Future studies with higher patient numbers may be able to determine differences in effect between types of surgery, investigate the effect in patients with CKD, and elucidate mechanisms of the association between bariatric surgery and AKI."
Generalisability	21	Discuss the generalisability (external validity) of the study results<br><br>Page 15/16: "Even though the data is taken from a representative sample of the UK population, the baseline data indicate that patients who undergo bariatric surgery are mostly female, of middle age, and with a history of T2DM. While the results were adjusted for age and sex they might not be applicable for other groups suffering from obesity like adolescents. Linkage between the CPRD and HES databases was restricted to England. However, there is no cogent reason why the results should not

For peer review only

		be applicable to regions with similar healthcare systems, both in the UK and internationally."
**Other information**		
Funding	22	Give the source of funding and the role of the funders for the present study and, if applicable, for the original study on which the present article is based<br><br>RM is supported by a Sir Henry Wellcome Postdoctoral Fellowship from the Wellcome Trust. KB holds a Sir Henry Dale fellowship jointly funded by the Wellcome Trust and the Royal Society. RLB is an NIHR Research Professor and supported by funding from the Rosetrees Trust and the Sir Jules Thorn Charitable Trust. LS is supported by a senior clinical fellowship from the Wellcome Trust. IJD is funded by an unrestricted grant from GlaxoSmithKline.

*Give information separately for exposed and unexposed groups.

Note: An Explanation and Elaboration article discusses each checklist item and gives methodological background and published examples of transparent reporting. The STROBE checklist is best used in conjunction with this article (freely available on the Web sites of PLoS Medicine at http://www.plosmedicine.org/, Annals of Internal Medicine at http://www.annals.org/, and Epidemiology at http://www.epidem.com/). Information on the STROBE Initiative is available at http://www.strobe-statement.org.

For peer review only

BMJ Open

Long-term effects of bariatric surgery on acute kidney injury: A propensity-matched cohort in the United Kingdom Clinical Practice Research Datalink

Journal:	*BMJ Open*
Manuscript ID	bmjopen-2017-020371.R2
Article Type:	Research
Date Submitted by the Author:	17-Apr-2018
Complete List of Authors:	Koppe, Uwe; Robert Koch Institut,<br>Nitsch, Dorothea; LSHTM; Royal Free London NHS Foundation Trust, Honorary consultant nephrologist<br>Mansfield, Kathryn; London School of Hygiene and Tropical Medicine, Non-communicable Disease Epidemiology<br>Mathur, Rohini; London School of Hygiene and Tropical Medicine, Department of Non-Communicable Disease Epidemiology; Queen Mary University of London, Centre for Primary Care & Public Health<br>Bhaskaran, Krishnan; LSHTM, NCDE<br>Batterham, Rachel; UCL, Centre for Obesity Research, Rayne Institute, Department of Medicine<br>Smeeth, Liam; London School of Hygiene and Tropical Medicine, Epidemiology and Population Health<br>Douglas, Ian; London School of Hygiene and Tropical Medicine, Epidemiology and Population Health
**Primary Subject Heading**:	Epidemiology
Secondary Subject Heading:	Renal medicine
Keywords:	Clinical Practice Research Datalink, Acute Kidney Injury, Obesity, Bariatric Surgery

SCHOLARONE™
Manuscripts

Title Long-term effects of bariatric surgery on acute kidney injury: A propensity-matched cohort in the United Kingdom Clinical Practice Research Datalink

Running headline Bariatric surgery and acute kidney injury

Authors Uwe Koppe[1,2*], Dorothea Nitsch[1,3], Kathryn E. Mansfield[1], Rohini Mathur[1], Krishnan Bhaskaran[1], Rachel L. Batterham[4,5,6], Liam Smeeth[1], Ian J. Douglas[1]

[1] Faculty of Epidemiology and Population Health, London School of Hygiene & Tropical Medicine, London, United Kingdom,

[2] Department of Infectious Disease Epidemiology, Robert Koch Institute, Berlin, Germany,

[3] Royal Free London NHS Trust, Honorary consultant nephrologist

[4] Centre for Obesity Research, Rayne Institute, Department of Medicine, University College London, London, United Kingdom,

[5] University College London Hospital Bariatric Centre for Weight Management and Metabolic Surgery, London, United Kingdom,

[6] National Institute of Health Research, University College London Hospital Biomedical Research Centre, London, United Kingdom

* Correspondence to:

Dr. Uwe Koppe
Robert Koch Institut
Seestr. 10
13353 Berlin, Germany
Tel: +49 30 18754 2262
Fax: +49 30 18754 3533
koppeu@rki.de

Word count: 3,486

For peer review only

ABSTRACT

Objective: Bariatric surgery is an effective method of weight reduction and has been associated with acute kidney injury (AKI) as a perioperative event. However, the long-term effects of the weight reduction after surgery on AKI are unknown. The objective of this study is to quantify the association of bariatric surgery with later risk of AKI.

Design: This study uses a propensity-score matched cohort of patients from the United Kingdom Clinical Practice Research Datalink database with and without bariatric surgery to compare rates of AKI episodes derived from linkage to the Hospital Episode Statistics.

Setting: England, United Kingdom

Participants: We included 2,643 patients with bariatric surgery and 2,595 patients without.

Results: Results were compatible with an increased risk of AKI in the first 30 days following surgery compared with patients without surgery, but AKI incidence was substantially decreased in patients with bariatric surgery during long-term follow-up (rate ratio 0.37, 95% CI 0.23, 0.61) even after accounting for chronic kidney disease status at baseline. Over the whole period of follow-up, bariatric surgery had a net protective effect on risk of AKI (rate ratio 0.45, 95% CI 0.28, 0.72).

Conclusions: Bariatric surgery was associated with protective effects on AKI incidence during long-term follow-up. While the risk of AKI may be increased within the first 30 days, the net effect seen was beneficial.

Keywords: Acute Kidney Injury; Obesity; Bariatric Surgery; Clinical Practice Research Datalink

For peer review only

STRENGTHS AND LIMITATIONS OF THIS STUDY

- This study uses high quality data from linked databases in England (Clinical Practice Research Datalink and Hospital Episode Statistics) to describe long-term effects of bariatric surgery on acute kidney injury (AKI) for the first time.
- Data are captured prospectively and continuously thus allowing follow-up of patients over long time periods.
- Outcome measures are obtained with standardised ICD-10 codes, which have been shown to accurately identify AKI, but do not allow grading of severity and may therefore underestimate true AKI incidence.
- Only AKI events recorded during a hospital admission were included in the analysis likely representing the more serious events of AKI.
- The study population was mostly female, of middle age, and had a history of type 2 diabetes mellitus. Thus the results might not be applicable for other groups suffering from obesity such as adolescents.

For peer review only

INTRODUCTION

The proportions of overweight and obese adults in England in 2014 are estimated to be 61.7% and 25.6%, respectively, and are increasing over time[1]. Obesity is associated with serious health consequences including type 2 diabetes mellitus (T2DM), cardiovascular diseases, cancers, and chronic kidney disease (CKD)[2-4]. Bariatric surgery has been shown to be a highly effective intervention for achieving weight loss and reducing the burden of co-morbidities, such as T2DM, metabolic syndrome, and hypertension[5 6]. A recent observational study on recipients of bariatric surgery from the United Kingdom (UK) confirmed sustained weight loss as well as resolution of T2DM and hypertension over a period of 4 years[7].

Acute kidney injury (AKI) is defined as a sudden (over hours or days) drop in kidney function characterised by increased serum creatinine and/or reduced urine output. AKI has been linked to increased in-hospital mortality, length of hospital stay, and subsequent development of CKD[8]. While T2DM, CKD, and obesity have been described as risk factors for AKI, it can also be precipitated by nephrotoxic drugs, surgical interventions, and sepsis[8-10]. AKI has been described as a short-term complication of bariatric surgery, stemming from rhabdomyolysis[10-16]. In addition, AKI has been linked to nephrolithiasis, which can develop over time after Roux-En-Y Gastric Bypass surgery[11 17]. To the best of our knowledge, no studies have been published examining the long-term effects of bariatric surgery on AKI.

In this study, we investigate the long-term effects of bariatric surgery on AKI to see whether the expected reduction in BMI has any impact on subsequent renal health. We used routinely collected electronic health record data from primary and secondary care. For this, we conducted a matched cohort study using prospectively collected data from patients in the United Kingdom Clinical Practice Research Datalink (CPRD).

For peer review only

METHODS

Patient and Public Involvement

Patients or public were not involved in the design or conduct of the study.

Study design

We undertook a matched cohort study using prospectively collected data from CPRD patients registered before 31st December 2014 linked to the Hospital Episodes Statistics (HES) database to investigate long-term effects of bariatric surgery on AKI.

Data source

The CPRD database contains anonymised, routinely collected data on approximately 10 million patients in participating primary care practices in the UK, including demographic characteristics, current and previous diagnoses, prescribing, test results, and lifestyle factors. Diagnoses, signs, and symptoms are recorded using Read codes[18]. Patients are broadly representative of the UK population and the data have been validated for a wide range of outcomes[19-21]. The HES database contains patient data from hospital admissions to English hospitals within the National Health Service[22]. For each hospital admission, the diagnoses are recorded using standardised codes of the International Classification of Diseases, Tenth Revision (ICD-10)[23 24]. Data from 70% of CPRD practices in England has been linked at patient level with HES admission data thus allowing the combined analysis of data from primary and acute hospital care for a subset of patients[19].

Cohort design and propensity matching

For peer review only

A detailed description of how the cohort was constructed is described elsewhere[7]. In brief, records of patients who underwent bariatric surgery (n=3,882) between 1997 and 2015 were matched to individuals who did not undergo surgery (n=3,882) using propensity scores.

Study population matching and the propensity score incorporated information on age, sex, calendar period, history of T2DM, hypertension, coronary heart disease, cerebrovascular disease, peripheral vascular disease, other atheroma, use of insulin, use of oral antidiabetic medication, use of statins, smoking status, and alcohol consumption.

Patients with bariatric surgery were identified using Read codes for surgery in the CPRD database (S1 Appendix) and were included in the study if they had been registered in the CPRD ≥12 months prior to the intervention. We excluded those with a record of prior bariatric surgery reversal.

For the comparison group, the inclusion criteria were to have at least one BMI measurement ≥40 kg/m^2 during their CPRD registration, which could span 10 years or more, ≥12 months of follow-up prior to the index date in the database, and no prior record of bariatric surgery or bariatric surgery reversal. Based on this, it is therefore possible that the BMI recorded closest to the index date was lower than 40 kg/m^2.

The study sample was restricted to eligible patients registered at practices linked to the HES database and information on AKI events was obtained, resulting in a final cohort comprising 2,643 patients who underwent bariatric surgery, and 2,595 patients who did not.

Follow-up started on the day of surgery for those with bariatric surgery, and for the comparison group who did not undergo bariatric surgery, on the surgery date of their matched case. Patient records were censored at the earliest of: AKI, death, leaving the

For peer review only - http://bmjopen.bmj.com/site/about/guidelines.xhtml

practice, latest data collection from current practice, or end of linkage period to the HES database.

Outcomes and covariates

The primary outcome of this study was the incidence rate of the first AKI episode during follow-up in patients with and without bariatric surgery. AKI episodes were obtained from the HES database using ICD-10 codes: N17.0 (“Acute kidney failure with tubular necrosis”), N17.1 (“Acute renal failure with acute cortical necrosis), N17.2 (“Acute renal failure with medullary necrosis”), N17.8 (“Other acute renal failure”), N17.9 (“Acute kidney failure, unspecified”), and N19 (“Unspecified kidney failure”). In this cohort, events coded with N17.1, N17.2, and N17.8 were not found. AKI events that occurred before the start of follow-up were recorded as a binary variable “history of AKI”, while AKI events occurring during follow-up were used to analyse AKI incidence.

Recorded serum creatinine values from the CPRD database were not routinely standardised with isotope-dilution mass spectrometry before 2013. Thus, we assumed all measurements to be unstandardized and multiplied the creatinine measures with the factor 0.95 before calculating the estimated glomerular filtration rate (eGFR) using the “Chronic Kidney Disease Epidemiology Collaboration” (CKD-EPI) equation[25]. Ethnicity was not considered in the eGFR calculation due to incomplete recording in the database and the low proportion of Afro-Caribbean people in the population. CKD stages were defined according to eGFR values in ml/min/1.73m^2 according to current guidelines[26]: eGFR ≥60 = no known CKD; eGFR 45-59 = stage 3a; eGFR 30-44 = stage 3b; eGFR 15-29 = stage 4; eGFR <15 = stage 5. Baseline CKD

status was derived from eGFR measurements in the year prior to start of follow-up by: 1) taking the last two measurements before the index date ≥90 days apart – with the higher eGFR value corresponding to the CKD baseline status, or 2) taking the most recent serum creatinine result if only one suitable test result was available. Since serum creatinine is more likely to be tested in the acutely unwell or in people who are routinely monitored as part of incentivised programs (e.g. people with diabetes), patients without measurements of CKD baseline status were assumed to have no CKD[27] and were analysed as such.

Statistical Analysis

Though propensity score matching was employed to minimise confounding, we compared the distribution of baseline characteristics between the exposed and unexposed groups to check for any imbalances that may be relevant to the outcome of AKI. The baseline distribution of categorical variables was analysed using percentages and χ^2-tests. Continuous variables were analysed as means with standard deviations for normally distributed variables and medians with interquartile ranges for non-normally distributed variables. Differences in continuous variables were analysed with Student's t-tests or Wilcoxon rank sum tests for normally and non-normally distributed data, respectively.

The association between bariatric surgery and AKI was analysed using a Poisson regression model with a time to first event analysis. P-values were calculated using Wald tests. In order to separate short-term effects of the surgery from potential long-term effects, we analysed the association separately for: a) events within the first 30 days, and b) events after 30 days. When the cohort was initially constructed, propensity score matching was used to deal with confounding [7]. This study uses a subset of this cohort since patients from practices without

For peer review only

186 linkage between the CPRD and HES databases had to be excluded (as AKI was assessed using hospital admission data). To identify variables for the multivariable model, potential confounders that were not deemed to be on the causal pathway were added individually to the univariable model. If the addition changed the effect estimate ≥10% these variables were included in the multivariable model. Consequently, history of AKI, history of taking oral antidiabetics, and BMI at baseline were included (S2 Appendix). In addition, age at baseline, sex, calendar period (1997-2005, 2006-2010, 2011-2015), and CKD status at baseline were selected *a priori* as forced variables. For models with <40 outcomes, only age and sex were included in the multivariable model due to data sparsity.

195 The 5% bands of patients with the highest and lowest propensity scores were excluded from the primary analysis ("trimming") since these contain patients that are treated in stark contrast to their health status, potentially causing bias [28].

198 Heterogeneity of effect estimates between the calendar periods was tested with a Likelihood Ratio Test.

200 The analysis was performed for all patients with bariatric surgery and also further stratified by type of surgery. Patients with stage 5 CKD (baseline eGFR < 15 ml/min/1.73m^2) were excluded from the analyses since this constitutes end-stage renal failure (ESRD). In addition, patients with missing data in ≥1 variable of the multivariable model were excluded from both uni- and multivariable analyses.

205 All analyses were performed with Stata 14.1.

207 Subgroup analyses

208 Several planned sensitivity analyses were undertaken: 1) To determine the net effect of the intervention we calculated the risk of AKI over the whole period of follow-up; 2) The

For peer review only

prevalence of decreased kidney function in the CPRD database was similar to that in a nationally representative kidney disease registry [27] indicating that patients with missing eGFR measurements are unlikely to have CKD. To identify potential differences in the effect between patients with known and unknown eGFR measurements, we restricted the analysis to a) patients known to have no CKD at baseline (baseline eGFR ≥ 60 ml/min/1.73m^2), b) patients without known CKD at baseline (as above but including patients with missing creatinine values at baseline and assuming these individuals to have no CKD), and c) patients with known CKD at baseline.[27]; 3) Moreover, to investigate the effect in a group of particular interest which is under more scrutiny for measuring kidney function we restricted the analysis to patients with: a) T2DM, and b) a history of taking insulin; 4) To avoid misclassification of low eGFR values as AKI [29] we excluded patients with stage 4 CKD at baseline; 5) We restricted the analysis to ICD-10 codes N17.0 and N17.9, which have a high positive predictive value for AKI [24]; 6) We increased the immediate post-surgery time span from 30 to 60 days; 7) We included people with extreme propensity scores; and 8)We excluded patients with a BMI<35 kg/m^2 at baseline.

Ethical approval

This study was approved by the London School of Hygiene & Tropical Medicine ethics committee (LSHTM MSc Ethics Ref: 11065) and the Independent Scientific Advisory Committee on Medicines & Healthcare Products Regulatory Agency database research (approval number: 16_106R).

231 **RESULTS**

Since linkage to the HES-database was only possible for patients whose GPs had agreed for their practice data to be linked to HES (S3 Appendix), there were 2,643 patients with bariatric surgery and 2,595 people without surgery resulting in a cohort of overall 5,238 people with a median follow-up of 2.9 years (Table 1). The median follow-up prior to baseline was similar between the groups: 8.8 years (IQR: 8.1 years) for patients with bariatric surgery and 9.3 years (IQR: 8.0 years) for people without surgery.

For peer review only

Table 1: Baseline data for CPRD/HES-linked cohort study of people with bariatric surgery and the corresponding propensity score-matched comparison cohort (data are n (%) unless otherwise specified)*

	Bariatric Surgery (n = 2,643)	Matched Comparison group without surgery (n = 2,595)	p-value[1]
**Follow-up (years), median (IQR)**	2.9 (3.2)	2.9 (3.4)	0.616
**Age (years), mean (SD)**	45.2 (10.7)	45.0 (10.8)	0.417
**17 – 39, n (%)**	818 (31.0)	826 (31.8)	
**40 – 49, n (%)**	945 (35.8)	928 (35.8)	0.727
**50 – 85, n (%)**	880 (33.3)	841 (32.4)	
**BMI at baseline, mean (SD)**	44.9 (8.9)	42.2 (6.5)	<0.001
**13 – 34, n (%)**	297 (11.2)	287 (11.1)	
**35 – 39, n (%)**	448 (17.0)	456 (17.6)	
**40 – 44, n (%)**	625 (23.7)	1,118 (43.1)	<0.001
**45 – 49, n (%)**	571 (21.6)	438 (16.9)	
**50 – 94, n (%)**	667 (25.2)	253 (9.8)	
**Missing, n (%)**	35 (1.3)	43 (1.7)	
**Female**	2,131 (80.6)	2,131 (82.1)	0.166
**History of**			
**Cerebrovascular disease**	37 (1.4)	26 (1.0)	0.186
**Coronary heart disease**	104 (3.9)	82 (3.2)	0.130
**Peripheral vascular disease**	11 (0.4)	15 (0.6)	0.405
**Other atheroma**	0	<5[2]	0.313
**T2DM**	900 (34.1)	853 (32.9)	0.365
**Taking oral antidiabetic**	571 (21.6)	455 (17.5)	<0.001
**Taking insulin**	180 (6.8)	156 (6.0)	0.238
**Hypertension**	890 (33.7)	869 (33.5)	0.886
**Statin use**	699 (26.4)	640 (24.7)	0.139
**AKI**	30 (1.1)	11 (0.4)	0.003
**Alcohol status**			
**Non-drinker**	435 (16.5)	397 (15.3)	
**Ex-drinker**	278 (10.5)	236 (9.1)	
**Current drinker (amount unknown)**	15 (0.6)	13 (0.5)	
**<2 units/day**	659 (24.9)	644 (24.8)	0.366
**3-6 units/day**	862 (32.6)	909 (35.0)	
**>6 units/day**	170 (6.4)	164 (6.3)	
**Unknown**	224 (8.5)	232 (8.9)	
**Smoking status**			
**Non-smoker**	1,126 (42.6)	1,151 (44.4)	
**Current smoker**	403 (15.3)	345 (13.3)	0.093
**Ex-smoker**	1,112 (42.1)	1,099 (42.4)	
**Unknown**	<5[2]	0	
**CKD at baseline**			
**Baseline CKD status absent**	1,119 (42.3)	1,299 (50.1)	
**No CKD**	1,470 (55.6)	1,242 (47.9)	
**Stage 3a**	27 (1.0)	37 (1.4)	<0.001
**Stage 3b**	16 (0.6)	10 (0.4)	
**Stage 4**	10 (0.4)	5 (0.2)	
**Stage 5**	<5[2]	<5[2]	
**Type of bariatric surgery**			
**Gastric band**	1,193 (45.1)		
**Sleeve gastrectomy**	364 (13.8)		
**Gastric bypass**	1,075 (40.7)		
**Other**	11 (0.4)		
**ICD-10 code for AKI during follow-up**	**n = 44**	**n = 62**	
**N17.0 (Acute kidney failure with tubular necrosis)**	<5[2]	<5[2]	
**N17.9 (Acute kidney failure, unspecified)**	38 (86.4)	52 (83.9)	0.927
**N19 (Unspecified kidney failure)**	5 (11.4)	8 (12.9)	

[1] categorical variables: χ^2-test; continuous variables: t-test + SD if normally distributed, rank sum test + IQR if non-normally distributed
[2]cell counts <5 have been suppressed to ensure anonymity
*In the original study, each surgery patient was matched 1:1 to the person without surgery with the closest propensity score, choosing matches at random where more than one possible match had the same score [7]
AKI = acute kidney injury, BMI = body mass index, CKD = chronic kidney disease, ICD-10 = International Classification of Diseases, Tenth Revision, IQR = interquartile range, SD = standard deviation, T2DM = type 2 diabetes mellitus

For peer review only

This cohort was comparable to the cohort from the original study regarding sex, mean age, mean BMI, history of T2DM, type of bariatric surgery and the imbalance of BMI at baseline [7]. More patients in the intervention group had a history of AKI compared to the comparison group (1.1% vs. 0.4%). Of the 106 included events during follow-up, 84.9% were classified with the ICD-10 code N17.9 ("acute kidney failure, unspecified"), 12.3% were coded as N19 ("unspecified kidney failure"), and 2.8% had a code of N17.0 ("Acute kidney failure with tubular necrosis"). CKD status at baseline was unknown for about half of the patients in each group with a slightly higher proportion in the unexposed group (50.1% vs. 42.3%). The majority of the patients with creatinine tests at baseline did not have CKD (96.2 %).

The number of AKI events recorded in the first 30 days of follow-up was low. All five events happened in patients with bariatric surgery and none were recorded in the control group, which is consistent with the possibility of an increased risk of AKI directly after surgery (Table 2).

For peer review only

Table 2: Association of bariatric surgery with first incident AKI, stratified by length of follow-up. Unexposed refers to the propensity matched comparison group

	PY	Events	Rate per 1000 PY (95% CI)	Crude RR (95% CI)[1]	p-value[2]	Adjusted RR (95% CI)[3]	p-value[2]
**All patients**							
**Day 1-30**							
Unexposed	203	0	0	-			
Bariatric surgery	199	5	25.1 (10.5, 60.4)	-			
**> Day 30**							
Unexposed	7,882	54	6.9 (5.2, 8.9)	-			
Bariatric surgery	8,061	34	4.2 (3.0, 5.9)	0.62 (0.40, 0.95)	0.027	0.37 (0.23, 0.61)	<0.001
**All patients analysed by type of surgery[4]**							
**Day 1-30**							
Unexposed							
Gastric band							
Sleeve gastrectomy							
Gastric bypass							
Other							
**> Day 30**							
Unexposed	7,882	54	6.9 (5.2, 8.9)	-			
Gastric band	4,614	17	3.7 (2.3, 5.9)	0.54 (0.31, 0.93)	0.026		
Sleeve gastrectomy	728	<5[5]	5.5 (2.1, 14.6)	0.80 (0.29, 2.21)	0.670		
Gastric bypass	2,655	13	4.9 (2.8, 8.4)	0.71 (0.39, 1.31)	0.277		
Other	63	0	-	-			
**All patients over whole period of follow-up**							
Unexposed	8,085	54	6.7 (5.1, 8.7)	-			
Bariatric surgery	8,259	39	4.7 (3.5, 6.5)	0.71 (0.47, 1.07)	0.099	0.45 (0.28, 0.72)	0.001

[1] Poisson regression model
[2] Wald test
[3] Poisson regression model adjusted for age at baseline, sex, calendar time, CKD at baseline, history of AKI, history of taking oral antidiabetics, and BMI at baseline
[4] No analysis for day 1-30 owing to sparse data
[5] Cell counts <5 have been suppressed to ensure anonymity
AKI = acute kidney injury, CKD = chronic kidney disease, PY = person-years, RR = rate ratio

From 30 days onwards, bariatric surgery had a protective association with AKI risk (crude RR = 0.62, 95% CI 0.40, 0.95). The effect estimate of the multivariable model indicated an even stronger protective effect associated with bariatric surgery (RR = 0.37, 95% CI 0.23, 0.61), largely due to the confounding by AKI prior to baseline.

The analysis by type of surgery yielded protective effect estimates for all types but the confidence intervals were wide and no comparison between individual procedures was feasible. Sensitivity analyses yielded similar results (S4 Appendix). A sensitivity analysis restricted to patients with known CKD at baseline could not be done owing to sparse data. Investigation of the effect of bariatric surgery over the whole follow-up period resulted in a protective net effect associated with the intervention in univariable (RR = 0.71, 95% CI 0.47, 1.07) and multivariable (RR = 0.45, 95% CI 0.28, 0.72) analyses.

For peer review only

DISCUSSION

In this study using prospectively recorded routine healthcare data from a representative sample in the UK, bariatric surgery was associated with a potentially increased risk of AKI within the first 30 days after surgery (5 events in patients with bariatric surgery, no events in control patients) but a strongly protective association thereafter (adjusted RR = 0.37, 95% CI 0.23, 0.61). The association was consistent across subgroups and sensitivity analyses. To the best of our knowledge, this is the first study to describe long-term effects of bariatric surgery on AKI.

AKI has been described as a perioperative event for bariatric surgery [12 13 15 16]. Our results are consistent with an increased risk in the early stages after surgery, however our analysis lacked enough early events to rule out chance as a reason for the results observed. Since patients do not have kidney function measures routinely checked by their family physician after bariatric surgery, many events could remain unnoticed. Patients with known CKD are more thoroughly checked for AKI and are a valuable subgroup to investigate, but the numbers in this dataset were too low to analyse.

This study uses high quality data from routine medical care in the UK. The healthcare system allows universal patient access to primary and secondary care so that the data is representative of the population. Patients are followed continuously while they are registered with a general practitioner allowing prospective data capture over long observation periods and avoiding problems with reverse causality.

Some limitations need to be considered. Even though the data is taken from a representative sample of the UK population, the baseline data indicate that patients who undergo bariatric surgery are mostly female, of middle age, and with a history of T2DM. While the results were adjusted for age and sex they might not be applicable for other

For peer review only

groups suffering from obesity like adolescents. Linkage between the CPRD and HES databases was restricted to England. However, there is no cogent reason why the results should not be applicable to regions with similar healthcare systems, both in the UK and internationally. We had insufficient data to determine whether the association with AKI varied between different types of bariatric surgery; we found a protective effect for gastric band but results were inconclusive for sleeve gastrectomy and gastric bypass.

Of all AKI episodes identified in the HES database, the ICD-10 codes N17.0 and N17.9 comprised 87.7% of all events and have previously been shown to accurately identify AKI in a single centre study [24]. However, there are no ICD-10 codes for grades of AKI severity. Thus, we were not able to investigate whether there was an association between bariatric surgery and AKI severity. In general, AKI diagnosed during hospitalisation is likely to represent more serious AKI events and therefore may underestimate AKI incidence. Thus, the conclusions drawn from this study may only be applicable to severe AKI diagnosed during hospitalisation, however, we would argue these are the most clinically relevant outcomes. Moreover, a patient who experienced a previous AKI episode might be under more scrutiny for detection of future episodes. Since more patients in the bariatric surgery group had a history of AKI they might have a higher chance of detection of an AKI episode during follow-up, which we adjusted for in our analyses.

For peer review only

Misclassification of diagnostic codes is likely non-differential between the bariatric surgery patients and the matched comparison group and would bias the effect towards the null value. However, it is also conceivable during the immediate post-operative period those undergoing bariatric surgery might have been under more scrutiny to detect potential AKI events than people without surgery. In this case our current relative risk estimate for the immediate postoperative period would be an overestimate. Another problem of primary

For peer review only - http://bmjopen.bmj.com/site/about/guidelines.xhtml

317 care data is that not every patient is routinely checked for their kidney function, as
318 incentives of testing apply primarily for those at risk of kidney disease due to diabetes and
319 hypertension. The study relied on AKI events recorded in HES as part of a hospital admission
320 and over time, the awareness of the importance of AKI has likely changed resulting in
321 secular changes in recording of AKI [30]; analyses have adjusted for calendar period to account
322 for this [23 31]. Future studies with hospital creatinine data should compare the AKI severity
323 between the groups to investigate this issue.

324 In addition, CKD status at baseline was missing in almost half of the patient population.
325 However, a recent study indicated that the prevalence of CKD in the CPRD database was
326 comparable to that found in nationally representative registry studies [27]. This indicates that
327 patients without a GP record of eGFR-measurements at baseline are unlikely to have CKD. In
328 addition, sensitivity analyses investigating the effect in patients with known or unknown
329 CKD status at baseline yielded comparable results.

330 Since access to bariatric surgery is restricted within the UK healthcare system, some
331 patients might have funded their operation privately, resulting in selection bias. In a recent
332 analysis about 40% of bariatric surgery operations in the UK were privately funded [32]. Thus,
333 the intervention group might have a higher socioeconomic status than the non-exposed
334 group, in which similar patients would not be able to afford surgery. Since the
335 socioeconomic background is an important determinant of health outcomes and was an
336 unmeasured potential confounder not considered in the matching process, this could have
337 led to more positive health outcomes in the intervention group irrespective of surgery and
338 to an overestimation of the effect. In this study setting it was not possible to determine
339 which patients had privately funded surgery.

For peer review only

Even though most baseline variables were evenly distributed due to the matching process this does not guarantee that unmeasured variables are evenly distributed as well, which can constitute residual confounding. Incorrect, imprecise, or missing measurements of covariates could also have led to residual confounding. For the multivariable model, adjusting for history of AKI led to the strongest change of the effect estimate. AKI events are likely under-recorded in the HES database, for reasons described above, and thus residual confounding is possible. Since adjusting for AKI history led to a stronger effect estimate, the protective effect we report here may be an underestimate if AKI history is missing to the same degree in surgery and non-surgery patients.

This study adds to the evidence of long term effects of bariatric surgery, and appears to be the first study to quantify a long-term beneficial effect on AKI. Future studies with higher patient numbers may be able to determine differences in effect between types of surgery, investigate the effect in patients with CKD, and elucidate mechanisms of the association between bariatric surgery and AKI.

For peer review only

SUPPLEMENTARY INFORMATION

Supplementary information is available at the *BMJ open* website.

FUNDING

There was no specific funding to conduct this research project.

RM is supported by a Sir Henry Wellcome Postdoctoral Fellowship from the Wellcome Trust. KB holds a Sir Henry Dale fellowship jointly funded by the Wellcome Trust and the Royal Society. RLB is an NIHR Research Professor and supported by funding from the Rosetrees Trust and the Sir Jules Thorn Charitable Trust. LS is supported by a senior clinical fellowship from the Wellcome Trust. IJD is funded by an unrestricted grant from GlaxoSmithKline. None of the funders had any involvement in the design of the study, the data collection and analysis, the writing of the report, or the decision to submit the paper for publication.

CONFLICT OF INTEREST

The authors have no conflicts of interest to disclose.

DATA SHARING

The data were obtained from the Clinical Practice Research Datalink (CPRD). CPRD data governance does not allow us to distribute patient data to other parties. Researchers may apply for data access at www.CPRD.com. The codes used to produce the data for this study are provided in the Supporting Information.

For peer review only

379 **CONTRIBUTIONS**

380 UK, DN, RLB, IJD, and LS were responsible for conceptualisation of the study and formulate
381 the research goals and aims. UK, DN, KEM, RM, KB, RLB, LS, and IJD developed the
382 methodology and models. UK, KEM, KB, IJD, and RM worked on the data curation. UK
383 performed the statistical analysis and wrote the original draft. UK, DN, KEM, RM, KB, RLB,
384 LS, and IJD reviewed and commented the draft and gave input on editing.

For peer review only

REFERENCES

1. Public Health England. Obesity - UK and Ireland prevalence and trends. *Available from: https://wwwnooorguk/NOO_about_obesity/adult_obesity/UK_prevalence_and_trends, accessed 25th August 2016*
2. Burton JO, Gray LJ, Webb DR, et al. Association of anthropometric obesity measures with chronic kidney disease risk in a non-diabetic patient population. *Nephrol Dial Transplant* 2012;27(5):1860-6. doi: 10.1093/ndt/gfr574
3. Nguyen S, Hsu CY. Excess weight as a risk factor for kidney failure. *Current Opinion in Nephrology and Hypertension* 2007;16(2):71-76.
4. World Health Organisation. Obesity and overweight - Fact sheet. *Available from: http://wwwwhoint/mediacentre/factsheets/fs311/en/, accessed 5th August 2016*
5. Colquitt JL, Pickett K, Loveman E, et al. Surgery for weight loss in adults. *Cochrane Database Syst Rev* 2014(8):CD003641. doi: 10.1002/14651858.CD003641.pub4
6. Gloy VL, Briel M, Bhatt DL, et al. Bariatric surgery versus non-surgical treatment for obesity: a systematic review and meta-analysis of randomised controlled trials. *BMJ* 2013;347:f5934. doi: 10.1136/bmj.f5934
7. Douglas IJ, Bhaskaran K, Batterham RL, et al. Bariatric Surgery in the United Kingdom: A Cohort Study of Weight Loss and Clinical Outcomes in Routine Clinical Care. *PLoS Med* 2015;12(12):e1001925. doi: 10.1371/journal.pmed.1001925
8. Lewington AK, S. Clinical Practice Guidelines - Acute Kidney Injury. *UK Renal Association* 2011
9. Shashaty MG, Meyer NJ, Localio AR, et al. African American race, obesity, and blood product transfusion are risk factors for acute kidney injury in critically ill trauma patients. *J Crit Care* 2012;27(5):496-504. doi: 10.1016/j.jcrc.2012.02.002
10. Suneja M, Kumar AB. Obesity and perioperative acute kidney injury: A focused review. *Journal of Critical Care* 2014;29(4)
11. Currie A, Chetwood A, Ahmed AR. Bariatric surgery and renal function. *Obesity Surgery* 2011;21(4):528-39.
12. Thakar CV, Kharat V, Blanck S, et al. Acute kidney injury after gastric bypass surgery. *Clin J Am Soc Nephrol* 2007;2(3):426-30. doi: 10.2215/CJN.03961106
13. Weingarten TN, Gurrieri C, McCaffrey JM, et al. Acute kidney injury following bariatric surgery. *Obesity Surgery* 2013;23(1):64-70.
14. Chakravartty S, Sarma DR, Patel AG. Rhabdomyolysis in bariatric surgery: A Systematic review. *Obesity Surgery* 2013;23(8):1333-40.
15. Abdullah HR, Tan TP, Vaez M, et al. Predictors of Perioperative Acute Kidney Injury in Obese Patients Undergoing Laparoscopic Bariatric Surgery: a Single-Centre Retrospective Cohort Study. *Obes Surg* 2016;26(7):1493-9. doi: 10.1007/s11695-015-1938-6
16. Sharma SK, McCauley J, Cottam D, et al. Acute changes in renal function after laparoscopic gastric surgery for morbid obesity. *Surg Obes Relat Dis* 2006;2(3):389-92. doi: 10.1016/j.soard.2006.02.002
17. Bhatti UH, Duffy AJ, Roberts KE, et al. Nephrolithiasis after bariatric surgery: A review of pathophysiologic mechanisms and procedural risk. *Int J Surg* 2016;36(Pt D):618-23. doi: 10.1016/j.ijsu.2016.11.025
18. Chisholm J. The Read clinical classification. *BMJ* 1990;300(6732):1092.

For peer review only

19. Williams T, van Staa T, Puri S, et al. Recent advances in the utility and use of the General Practice Research Database as an example of a UK Primary Care Data resource. *Ther Adv Drug Saf* 2012;3(2):89-99. doi: 10.1177/2042098611435911
20. Herrett E, Gallagher AM, Bhaskaran K, et al. Data Resource Profile: Clinical Practice Research Datalink (CPRD). *Int J Epidemiol* 2015;44(3):827-36. doi: 10.1093/ije/dyv098
21. Herrett E, Thomas SL, Schoonen WM, et al. Validation and validity of diagnoses in the General Practice Research Database: a systematic review. *Br J Clin Pharmacol* 2010;69(1):4-14. doi: 10.1111/j.1365-2125.2009.03537.x
22. National Health Service. The Information Centre. Hospital Episode Statistics. *Available from: http://digitalnhsuk/hes, accessed 12th August 2016*
23. McDonald HI, Shaw C, Thomas SL, et al. Methodological challenges when carrying out research on CKD and AKI using routine electronic health records. *Kidney Int* 2016 doi: 10.1016/j.kint.2016.04.010
24. Tomlinson LA, Riding AM, Payne RA, et al. The accuracy of diagnostic coding for acute kidney injury in England - a single centre study. *BMC Nephrol* 2013;14:58. doi: 10.1186/1471-2369-14-58
25. Levey AS, Stevens LA, Schmid CH, et al. A new equation to estimate glomerular filtration rate. *Ann Intern Med* 2009;150(9):604-12.
26. National Institute for Health and Care Excellence. Chronic kidney disease in adults: assessment and management, Clinical guideline. 2014
27. Iwagami M, Tomlinson LA, Mansfield KE, et al. Validity of estimated prevalence of decreased kidney function and renal replacement therapy from primary care electronic health records compared with national survey and registry data in the United Kingdom. *Nephrol Dial Transplant* 2017 doi: 10.1093/ndt/gfw318
28. Sturmer T, Rothman KJ, Avorn J, et al. Treatment effects in the presence of unmeasured confounding: dealing with observations in the tails of the propensity score distribution--a simulation study. *Am J Epidemiol* 2010;172(7):843-54. doi: 10.1093/aje/kwq198
29. Hill RS. Acute Kidney Injury Warning Algorithm - Best Practice Guidance 2014 [Available from: https://www.thinkkidneys.nhs.uk/wp-content/uploads/2014/12/AKI-Warning-Algorithm-Best-Practice-Guidance-final-publication-0112141.pdf.
30. McDonald HI, Shaw C, Thomas SL, et al. Methodological challenges when carrying out research on CKD and AKI using routine electronic health records. *Kidney Int* 2016;90(5):943-49. doi: 10.1016/j.kint.2016.04.010
31. James M, Pannu N. Methodological considerations for observational studies of acute kidney injury using existing data sources. *J Nephrol* 2009;22(3):295-305.
32. Gulliford MC, Charlton J, Booth HP, et al. Costs and outcomes of increasing access to bariatric surgery for obesity: cohort study and cost-effectiveness analysis using electronic health records. Southampton (UK)2016.

For peer review only

Long-term effects of bariatric surgery on acute kidney injury: A propensity-matched cohort in the United Kingdom Clinical Practice Research Datalink

Supporting Information

Overview

S1 Appendix – Code List for Identification of patients with bariatric surgery

S2 Appendix – Association of potential confounders with bariatric surgery and AKI

S3 Appendix – Patient selection from the original cohort

S4 Appendix – Sensitivity Analyses

For peer review only

S1 Appendix

Appendix 1: Code List for identification of patients with bariatric surgery from the CPRD database as published by Douglas et al. [7]

Read code	*description*
76132.00	Laparoscopic adjustable gastric banding
76134.00	Partitioning of stomach using staples
76131.11	Mason vertical banded gastroplasty
76133.00	Partitioning of stomach using band
76116.00	Laparoscopic sleeve gastrectomy
76115.00	Sleeve gastrectomy NEC
76425.00	Duodenal switch
76135.00	Partitioning of stomach NEC
76114.00	Sleeve gastrectomy and duodenal switch
76166.00	Laparoscopic gastric bypass

For peer review only

S2 Appendix

Appendix 2: Identification of potential confounders in the association of bariatric surgery (exposure) and the endpoint of incident AKI (outcome) in patients of the linked CPRD/HES cohort

	RR (95%CI)	Change in %	Selection for multivariable model
**Crude effect estimate**	0.62 (0.40, 0.95)		
**Effect estimates when individually adjusting for**			
**Age**	0.62 (0.40, 0.95)	0.2 %	yes (a priori)
**Sex**	0.60 (0.39, 0.92)	2.7 %	yes (a priori)
**Calendar Time**	0.61 (0.40, 0.94)	0.9%	yes (a priori)
**CKD status at baseline**	0.59 (0.38, 0.91)	4.4 %	yes (a priori)
**BMI at baseline**	0.53 (0.34, 0.83)	13.9 %	yes
**Alcohol Status**	0.61 (0.40, 0.93)	1.3 %	no
**Smoking Status**	0.61 (0.40, 0.94)	0.3 %	no
**History of cerebrovascular disease**	0.61 (0.40, 0.94)	0.6 %	no
**History of coronary heart disease**	0.60 (0.39, 0.91)	3.3 %	no
**History of peripheral vascular disease**	0.64 (0.41, 0.98)	3.2 %	no
**History of other atheroma**	0.62 (0.40, 0.95)	0.0 %	no
**History of diabetes**	0.60 (0.39, 0.92)	2.7%	no
**History of taking oral antidiabetics**	0.55 (0.36, 0.85)	10.4%	yes
**History of taking insulin**	0.57 (0.37, 0.87)	7.9 %	no
**History of hypertension**	0.61 (0.40, 0.94)	1.1 %	no
**History of statin use**	0.58 (0.38, 0.89)	5.5 %	no
**History of AKI**	0.42 (0.26, 0.67)	31.9 %	yes

Variables were added individually to the univariable model testing the association between bariatric surgery and AKI. If the addition of the respective variable changed the model ≥10% then the variable was selected to be included in the multivariable model.
AKI = acute kidney injury, BMI = body mass index, CKD = chronic kidney disease

For peer review only

S3 Appendix

Appendix 3: Patient selection from the original cohort as described in Douglas et al [7]

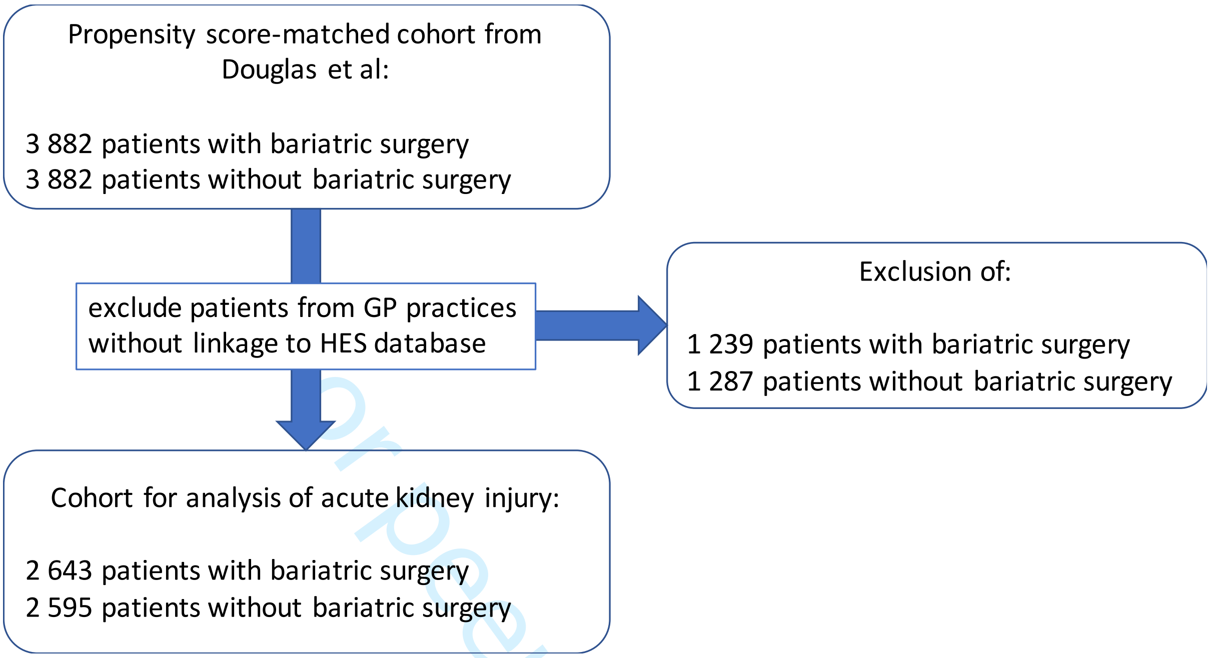

For peer review only

S4 Appendix

Appendix 4: Sensitivity analyses for the association of bariatric surgery with acute kidney injury

	PY	Events	Rate per 1000 PY (95% CI)	Crude RR (95% CI)[1]	p-value[2]	Adjusted RR (95% CI)[3]	p-value[2]
**Restricted to patients without CKD at baseline (available serum creatinine measures + eGFR ≥60)**							
**Day 1-30**							
Unexposed	98	0	0	-			
Bariatric surgery	111	<5[6]	36.2 (13.6, 96.3)	-			
**>Day 30**							
Unexposed	3,550	27	7.6 (5.2, 11.1)	-			
Bariatric surgery	4,311	22	5.1 (3.4, 7.7)	0.67 (0.38, 1.18)	0.165	0.53 (0.29, 1.00)	0.050
**Restricted to patients without known CKD at baseline (available serum creatinine measures + eGFR ≥60 or missing eGFR at baseline)**							
**Day 1-30**							
Unexposed	199	0	0	-			
Bariatric surgery	195	<5[6]	20.5 (7.7, 54.7)	-			
**>Day 30**							
Unexposed	7,735	42	5.4 (4.0, 7.3)	-			
Bariatric surgery	7,930	27	3.4 (2.3, 5.0)	0.63 (0.39, 1.02)	0.058	0.42 (0.25, 0.73)	0.002
**Excluding patients with CKD stage 4**							
**Day 1-30**							
Unexposed	203	0	0	-			
Bariatric surgery	198	5	25.2 (10.5, 60.6)	-			
**> Day 30**							
Unexposed	7,875	52	6.6 (5.0, 8.7)	-			
Bariatric surgery	8,037	32	4.0 (2.8, 5.6)	0.60 (0.39, 0.94)	0.024	0.35 (0.21, 0.59)	<0.001
**Restricted to patients with T2DM**							
**Day 1-30**							
Unexposed	65	0	0	-			
Bariatric surgery	69	<5[6]	43.6 (14.1, 135.1)	-			
**>Day 30**							
Unexposed	2,325	33	14.2 (10.1, 20.0)	-			
Bariatric surgery	2,548	18	7.1 (4.5, 11.2)	0.50 (0.28, 0.88)	0.017	0.25 (0.13, 0.51)	<0.001
**Restricted to patients with a history of taking insulin**							
**Day 1-30**							
Unexposed	11	0	0	-			
Bariatric surgery	13	0	0	-			
**>Day 30**							
Unexposed	321	11	34.3 (19.0, 61.9)	-			
Bariatric surgery	502	9	17.9 (9.3, 34.5)	0.52 (0.22, 1.26)	0.150	0.22 (0.08, 0.64)	0.005
**Restricted to ICD-10 codes N17.0 and N17.9**							
**Day 1-30**							
Unexposed	202	0	0	-			
Bariatric surgery	199	5	25.2 (10.5, 60.5)	-			
**>Day 30**							
Unexposed	7,871	48	6.1 (4.6, 8.1)	-			
Bariatric surgery	8,055	31	3.8 (2.7, 5.5)	0.63 (0.40, 0.99)	0.046	0.40 (0.24, 0.67)	<0.001
**Having an initial post-surgery time span of 60 days instead of 30**							
**Day 1-60**							
Unexposed	403	<5[6]	2.5 (0.3, 17.6)	-			
Bariatric surgery	395	6	15.2 (6.8, 33.8)	6.11 (0.74, 50.8)	0.094	[4]	
**> Day 60**							
Unexposed	7,682	53	6.9 (5.3, 9.0)	-			
Bariatric surgery	7,864	33	4.2 (3.0, 5.9)	0.61 (0.39, 0.94)	0.025	0.38 (0.23, 0.63)	<0.001
				Test for interaction[5]	0.011		

**Including patients with extreme propensity scores**							
**Day 1-30**							
Unexposed	208	0	0	-			
Bariatric surgery	206	5	24.3 (10.1, 58.3)	-			
> **Day 30**							
Unexposed	8,054	59	7.3 (5.7, 9.5)	-			
Bariatric surgery	8,324	34	4.1 (2.9, 5.7)	0.56 (0.37, 0.85)	0.007	0.33 (0.20, 0.54)	<0.001
**Excluding patients with BMI < 35 kg/m² at baseline**							
**Day 1-30**							
Unexposed	180	0	0	-			
Bariatric surgery	175	<5[6]	22.8 (8.6, 60.9)	-			
> **Day 30**							
Unexposed	6,714	48	7.1 (5.4, 9.5)	-			
Bariatric surgery	7,100	32	4.5 (3.2, 6.4)	0.63 (0.40, 0.99)	0.043	0.39 (0.23, 0.65)	<0.001

[1] Poisson regression model
[2] Wald test for RR, Likelihood-Ratio Test for interaction
[3] Poisson regression model adjusted for age at baseline, sex, calendar time, CKD at baseline, history of AKI, history of taking oral antidiabetics, and BMI at baseline
[4] No analysis for day 1-30 owing to sparse data
[5] Test for interaction of the effect estimate with the time periods 1-30 days and >30 days
[6] cell counts <5 have been suppressed to ensure anonymity
AKI = acute kidney injury, CKD = chronic kidney disease, PY = person-years, RR = rate ratio

For peer review only

Supporting Information:
STROBE statement checklist to ensure appropriate reporting of study information of long-term effects of acute kidney injury for the propensity-matched cohort study of patients with and without bariatric surgery

	Item No	Report
**Title and abstract**	1	(*a*) Indicate the study's design with a commonly used term in the title or the abstract<br><br>"Long-term effects of bariatric surgery on acute kidney injury: A propensity-matched cohort in the United Kingdom Clinical Practice Research Datalink"
		b) Provide in the abstract an informative and balanced summary of what was done and what was found<br><br>Abstract: on page 2 containing Background, Methods, Results and Conclusions
**Introduction**		
Background/rationale	2	Explain the scientific background and rationale for the investigation being reported<br><br>See page 4 for description of background;<br>Rationale (p4): "To the best of our knowledge, no studies have been published examining the long-term effects of bariatric surgery on AKI."
Objectives	3	State specific objectives, including any prespecified hypotheses<br><br>Page 4: "In this study, we investigate the long-term effects of bariatric surgery on AKI to see whether the expected reduction in BMI has any impact on subsequent renal health."
**Methods**		
Study design	4	Present key elements of study design early in the paper<br><br>See page 5: "We undertook a matched cohort study using prospectively collected data from CPRD patients registered before 31st December 2014 linked to the Hospital Episodes Statistics (HES) database to investigate long-term effects of bariatric surgery on AKI."
Setting	5	Describe the setting, locations, and relevant dates, including periods of recruitment, exposure, follow-up, and data collection<br><br>Page 5: "The CPRD database contains anonymised, routinely collected data on approximately 10 million patients in participating primary care practices in the UK, including demographic characteristics, current and previous diagnoses, prescribing, test results, and lifestyle factors. […].The HES database contains patient data from hospital admissions to English hospitals within the National Health Service […].Data from 70% of CPRD practices in England has been linked at patient level with HES admission data thus allowing the combined analysis of data from primary and acute hospital care for a subset of patients."<br>"A detailed description of how the cohort was constructed is described elsewhere. In brief, records of patients who underwent bariatric surgery (n=3,882) between 1997 and 2015 were matched to individuals who did not undergo surgery (n=3,882) using propensity scores."<br>Page 6: "Follow-up started on the day of surgery for those with bariatric surgery, and for the comparison group who did not undergo bariatric surgery, on the surgery date

For peer review only - http://bmjopen.bmj.com/site/about/guidelines.xhtml
For peer review only

		of their matched case. Patient records were censored at the earliest of: AKI, death, leaving the practice, latest data collection from current practice, or end of linkage period to the HES database.”
Participants	6	(*a*) Give the eligibility criteria, and the sources and methods of selection of participants. Describe methods of follow-up<br><br>Page 6: “Patients with bariatric surgery were identified using Read codes for surgery in the CPRD database (S1 Appendix) and were included in the study if they had been registered in the CPRD ≥12 months prior to the intervention. We excluded those with a record of prior bariatric surgery reversal.<br>For the comparison group, the inclusion criteria were to have at least one BMI measurement ≥40 kg/m2, ≥12 months of follow-up prior to the index date in the database, and no prior record of bariatric surgery or bariatric surgery reversal.”<br><br>Page 5: “The CPRD database contains anonymised, routinely collected data on approximately 10 million patients in participating primary care practices in the UK, including demographic characteristics, current and previous diagnoses, prescribing, test results, and lifestyle factors. Diagnoses, signs, and symptoms are recorded using Read codes […]. The HES database contains patient data from hospital admissions to English hospitals within the National Health Service “
		(*b*) For matched studies, give matching criteria and number of exposed and unexposed<br><br>Page 6: “Study population matching and the propensity score incorporated information on age, sex, calendar period, history of T2DM, hypertension, coronary heart disease, cerebrovascular disease, peripheral vascular disease, other atheroma, use of insulin, use of oral antidiabetic medication, use of statins, smoking status, and alcohol consumption.”<br>“The study sample was restricted to eligible patients registered at practices linked to the HES database and information on AKI events was obtained, resulting in a final cohort comprising 2,643 patients who underwent bariatric surgery, and 2,595 patients who did not.”
Variables	7	Clearly define all outcomes, exposures, predictors, potential confounders, and effect modifiers. Give diagnostic criteria, if applicable<br><br>Page 6/7: “AKI episodes were obtained from the HES database using ICD-10 codes: N17.0 (“Acute kidney failure with tubular necrosis”), N17.1 (“Acute renal failure with acute cortical necrosis), N17.2 (“Acute renal failure with medullary necrosis”), N17.8 (“Other acute renal failure”), N17.9 (“Acute kidney failure, unspecified”), and N19 (“Unspecified kidney failure”). In this cohort, events coded with N17.1, N17.2, and N17.8 were not found. AKI events that occurred before the start of follow-up were recorded as a binary variable “history of AKI”, while AKI events occurring during follow-up were used to analyse AKI incidence.<br>Recorded serum creatinine values from the CPRD database were not routinely standardised with isotope-dilution mass spectrometry before 2013. Thus, we assumed all measurements to be unstandardized and multiplied the creatinine measures with the factor 0.95 before calculating the estimated glomerular filtration rate (eGFR) using the “Chronic Kidney Disease Epidemiology Collaboration” (CKD-EPI) equation.

For peer review only

For peer review only - http://bmjopen.bmj.com/site/about/guidelines.xhtml

Ethnicity was not considered in the eGFR calculation due to incomplete recording in the database and the low proportion of Afro-Caribbean people in the population. CKD stages were defined according to eGFR values in ml/min/1.73m^2 according to current guidelines: eGFR ≥60 = no known CKD; eGFR 45-59 = stage 3a; eGFR 30-44 = stage 3b; eGFR 15-29 = stage 4; eGFR <15 = stage 5. Baseline CKD status was derived from eGFR measurements in the year prior to start of follow-up by: 1) taking the last two measurements before the index date ≥90 days apart – with the higher eGFR value corresponding to the CKD baseline status, or 2) taking the most recent serum creatinine result if only one suitable test result was available. Since serum creatinine is more likely to be tested in the acutely unwell or in people who are routinely monitored as part of incentivised programs (e.g. people with diabetes), patients without measurements of CKD baseline status were assumed to have no CKD and were analysed as such."

Page 6: "Study population matching and the propensity score incorporated information on age, sex, calendar period, history of T2DM, hypertension, coronary heart disease, cerebrovascular disease, peripheral vascular disease, other atheroma, use of insulin, use of oral antidiabetic medication, use of statins, smoking status, and alcohol consumption."

Page 8: "When the cohort was initially constructed, propensity score matching was used to deal with confounding. This study uses a subset of this cohort since patients from practices without linkage between the CPRD and HES databases had to be excluded (as AKI was assessed using hospital admission data). To identify variables for the multivariable model, potential confounders that were not deemed to be on the causal pathway were added individually to the univariable model. If the addition changed the effect estimate ≥10% these variables were included in the multivariable model. Consequently, history of AKI, history of taking oral antidiabetics, and BMI at baseline were included (S2 Appendix). In addition, age at baseline, sex, calendar period (1997-2005, 2006-2010, 2011-2015), and CKD status at baseline were selected a priori as forced variables."

Page 8: "In order to separate short-term effects of the surgery from potential long-term effects, we analysed the association separately for: a) events within the first 30 days, and b) events after 30 days."

Data sources/ measurement	8*	For each variable of interest, give sources of data and details of methods of assessment (measurement). Describe comparability of assessment methods if there is more than one group<br><br>Page 5: "The CPRD database contains anonymised, routinely collected data on approximately 10 million patients in participating primary care practices in the UK, including demographic characteristics, current and previous diagnoses, prescribing, test results, and lifestyle factors. Diagnoses, signs, and symptoms are recorded using Read codes […]The HES database contains patient data from hospital admissions to English hospitals within the National Health Service. For each hospital admission, the diagnoses are recorded using standardised codes of the International Classification of Diseases, Tenth Revision (ICD-10). Data from 70% of CPRD practices in England has been linked at patient level with HES admission data thus allowing the combined analysis of data from primary and acute hospital care for a subset of patients."<br>Page 5: "Patients with bariatric surgery were identified using Read codes for surgery in the CPRD database (S1 Appendix) and were included in the study if they had been

For peer review only - http://bmjopen.bmj.com/site/about/guidelines.xhtml

		registered in the CPRD ≥12 months prior to the intervention. We excluded those with a record of prior bariatric surgery reversal."<br>Page 6/7: "AKI episodes were obtained from the HES database using ICD-10 codes: N17.0 ("Acute kidney failure with tubular necrosis"), N17.1 ("Acute renal failure with acute cortical necrosis), N17.2 ("Acute renal failure with medullary necrosis"), N17.8 ("Other acute renal failure"), N17.9 ("Acute kidney failure, unspecified"), and N19 ("Unspecified kidney failure")."
Bias	9	Describe any efforts to address potential sources of bias<br><br>Page 9: "The 5% bands of patients with the highest and lowest propensity scores were excluded from the primary analysis ("trimming") since these contain patients that are treated in stark contrast to their health status, potentially causing bias."
Study size	10	Explain how the study size was arrived at<br><br>Pages 5/6: "A detailed description of how the cohort was constructed is described elsewhere. In brief, records of patients who underwent bariatric surgery (n=3,882) between 1997 and 2015 were matched to individuals who did not undergo surgery (n=3,882) using propensity scores." […] "The study sample was restricted to eligible patients registered at practices linked to the HES database and information on AKI events was obtained, resulting in a final cohort comprising 2,643 patients who underwent bariatric surgery, and 2,595 patients who did not."
Quantitative variables	11	Explain how quantitative variables were handled in the analyses. If applicable, describe which groupings were chosen and why<br><br>Page 7: "Recorded serum creatinine values from the CPRD database were not routinely standardised with isotope-dilution mass spectrometry before 2013. Thus, we assumed all measurements to be unstandardized and multiplied the creatinine measures with the factor 0.95 before calculating the estimated glomerular filtration rate (eGFR) using the "Chronic Kidney Disease Epidemiology Collaboration" (CKD-EPI) equation. Ethnicity was not considered in the eGFR calculation due to incomplete recording in the database and the low proportion of Afro-Caribbean people in the population. CKD stages were defined according to eGFR values in ml/min/1.73m2 according to current guidelines: eGFR ≥60 = no known CKD; eGFR 45-59 = stage 3a; eGFR 30-44 = stage 3b; eGFR 15-29 = stage 4; eGFR <15 = stage 5. Baseline CKD status was derived from eGFR measurements in the year prior to start of follow-up by: 1) taking the last two measurements before the index date ≥90 days apart – with the higher eGFR value corresponding to the CKD baseline status, or 2) taking the most recent serum creatinine result if only one suitable test result was available. Since serum creatinine is more likely to be tested in the acutely unwell or in people who are routinely monitored as part of incentivised programs (e.g. people with diabetes), patients without measurements of CKD baseline status were assumed to have no CKD and were analysed as such."<br>Page 8: "In addition, age at baseline, sex, calendar period (1997-2005, 2006-2010, 2011-2015), and CKD status at baseline were selected a priori as forced variables."
Statistical methods	12	(*a*) Describe all statistical methods, including those used to control for confounding<br><br>Page 8/9: "The association between bariatric surgery and AKI was analysed using a Poisson regression model with a time to first event analysis. P-values were calculated

For peer review only

using Wald tests. In order to separate short-term effects of the surgery from potential long-term effects, we analysed the association separately for: a) events within the first 30 days, and b) events after 30 days. When the cohort was initially constructed, propensity score matching was used to deal with confounding. This study uses a subset of this cohort since patients from practices without linkage between the CPRD and HES databases had to be excluded (as AKI was assessed using hospital admission data). To identify variables for the multivariable model, potential confounders that were not deemed to be on the causal pathway were added individually to the univariable model. If the addition changed the effect estimate ≥10% these variables were included in the multivariable model. Consequently, history of AKI, history of taking oral antidiabetics, and BMI at baseline were included (S2 Appendix). In addition, age at baseline, sex, calendar period (1997-2005, 2006-2010, 2011-2015), and CKD status at baseline were selected a priori as forced variables. For models with <40 outcomes, only age and sex were included in the multivariable model due to data sparsity.

The 5% bands of patients with the highest and lowest propensity scores were excluded from the primary analysis ("trimming") since these contain patients that are treated in stark contrast to their health status, potentially causing bias.

Heterogeneity of effect estimates between the calendar periods was tested with a Likelihood Ratio Test.

The analysis was performed for all patients with bariatric surgery and also further stratified by type of surgery. Patients with stage 5 CKD (baseline eGFR < 15 ml/min/1.73m2) were excluded from the analyses since this constitutes end-stage renal failure (ESRD). In addition, patients with missing data in ≥1 variable of the multivariable model were excluded from both uni- and multivariable analyses."

(*b*) Describe any methods used to examine subgroups and interactions

page 8: "In order to separate short-term effects of the surgery from potential long-term effects, we analysed the association separately for: a) events within the first 30 days, and b) events after 30 days."

page 9: "Heterogeneity of effect estimates between the calendar periods was tested with a Likelihood Ratio Test."

Page 9: "The analysis was performed for all patients with bariatric surgery and also further stratified by type of surgery."

(*c*) Explain how missing data were addressed

page 7: "Since serum creatinine is more likely to be tested in the acutely unwell or in people who are routinely monitored as part of incentivised programs (e.g. people with diabetes), patients without measurements of CKD baseline status were assumed to have no CKD and were analysed as such."

page 9: "In addition, patients with missing data in ≥1 variable of the multivariable model were excluded from both uni- and multivariable analyses."

(*d*) If applicable, explain how loss to follow-up was addressed

page 6: "Patient records were censored at the earliest of: AKI, death, leaving the practice, latest data collection from current practice, or end of linkage period to the

For peer review only

		HES database.”
		(*e*) Describe any sensitivity analyses<br><br>Pages 9/10: “Several planned sensitivity analyses were undertaken: 1) To determine the net effect of the intervention we calculated the risk of AKI over the whole period of follow-up; 2) The prevalence of decreased kidney function in the CPRD database was similar to that in a nationally representative kidney disease registry indicating that patients with missing eGFR measurements are unlikely to have CKD. To identify potential differences in the effect between patients with known and unknown eGFR measurements, we restricted the analysis to a) patients known to have no CKD at baseline (baseline eGFR $\geq$ 60 ml/min/1.73m^2), b) patients without known CKD at baseline (as above but including patients with missing creatinine values at baseline and assuming these individuals to have no CKD), and c) patients with known CKD at baseline.; 3) Moreover, to investigate the effect in a group of particular interest which is under more scrutiny for measuring kidney function we restricted the analysis to patients with: a) T2DM, and b) a history of taking insulin; 4) To avoid misclassification of low eGFR values as AKI we excluded patients with stage 4 CKD at baseline; 5) We restricted the analysis to ICD-10 codes N17.0 and N17.9, which have a high positive predictive value for AKI; 6) We increased the immediate post-surgery time span from 30 to 60 days; and 7) We included people with extreme propensity scores.”
**Results**		
Participants	13*	(a) Report numbers of individuals at each stage of study—eg numbers potentially eligible, examined for eligibility, confirmed eligible, included in the study, completing follow-up, and analysed<br><br>see S3 Appendix
		(b) Give reasons for non-participation at each stage<br><br>not applicable
		(c) Consider use of a flow diagram<br><br>see S3 Appendix
Descriptive data	14*	(a) Give characteristics of study participants (eg demographic, clinical, social) and information on exposures and potential confounders<br><br>page 12: see Table 1
		(b) Indicate number of participants with missing data for each variable of interest<br><br>page 12: see Table 1
		(c) Summarise follow-up time (eg, average and total amount)<br><br>page 12: see Table 1
Outcome data	15*	Report numbers of outcome events or summary measures over time<br><br>page 12: see Table 1
Main results	16	(*a*) Give unadjusted estimates and, if applicable, confounder-adjusted estimates and their precision (eg, 95% confidence interval). Make clear which confounders were

For peer review only

For peer review only - http://bmjopen.bmj.com/site/about/guidelines.xhtml

		adjusted for and why they were included<br><br>page 13: see Table 2
		(*b*) Report category boundaries when continuous variables were categorized<br><br>page 12: see Table 1
		(*c*) If relevant, consider translating estimates of relative risk into absolute risk for a meaningful time period<br><br>not applicable
Other analyses	17	Report other analyses done—eg analyses of subgroups and interactions, and sensitivity analyses<br><br>Page 13: see table 2<br>see S4 Appendix
**Discussion**		
Key results	18	Summarise key results with reference to study objectives<br><br>Page 15: "In this study using prospectively recorded routine healthcare data from a representative sample in the UK, bariatric surgery was associated with a potentially increased risk of AKI within the first 30 days after surgery (5 events in patients with bariatric surgery, no events in control patients) but a strongly protective association thereafter (adjusted RR = 0.37, 95% CI 0.23, 0.61). The association was consistent across subgroups and sensitivity analyses. To the best of our knowledge, this is the first study to describe long-term effects of bariatric surgery on AKI."
Limitations	19	Discuss limitations of the study, taking into account sources of potential bias or imprecision. Discuss both direction and magnitude of any potential bias<br><br>Pages 15-18: "Some limitations need to be considered. Even though the data is taken from a representative sample of the UK population, the baseline data indicate that patients who undergo bariatric surgery are mostly female, of middle age, and with a history of T2DM. While the results were adjusted for age and sex they might not be applicable for other groups suffering from obesity like adolescents. Linkage between the CPRD and HES databases was restricted to England. However, there is no cogent reason why the results should not be applicable to regions with similar healthcare systems, both in the UK and internationally. We had insufficient data to determine whether the association with AKI varied between different types of bariatric surgery; we found a protective effect for gastric band but results were inconclusive for sleeve gastrectomy and gastric bypass.<br>Any misclassification of diagnostic codes is likely non-differential between the bariatric surgery patients and the matched comparison group and would bias the effect towards the null value. Another problem of primary care data is that not every patient is routinely checked for their kidney function, as incentives of testing apply primarily for those at risk of kidney disease due to diabetes and hypertension. The study relied on AKI events recorded in HES as part of a hospital admission and over time, the awareness of the importance of AKI has likely changed resulting in secular changes in recording of AKI ; analyses have adjusted for calendar period to account for this. Future studies with hospital creatinine data should compare the AKI severity between the groups to investigate this issue. In general, AKI diagnosed during hospitalisation

For peer review only

		is likely to represent more serious AKI events, though we would argue these are the most clinically relevant outcomes. Moreover, a patient who experienced a previous AKI episode might be under more scrutiny for detection of future episodes. Since more patients in the bariatric surgery group had a history of AKI they might have a higher chance of detection of an AKI episode during follow-up. This would bias the estimate towards the null value and could indicate that the association we report is an under-estimate.<br>In addition, CKD status at baseline was missing in almost half of the patient population. However, a recent study indicated that the prevalence of CKD in the CPRD database was comparable to that found in nationally representative registry studies. This indicates that patients without eGFR-measurements at baseline are unlikely to have CKD. In addition, sensitivity analyses investigating the effect in patients with known or unknown CKD status at baseline yielded comparable results.<br>Since access to bariatric surgery is restricted within the UK healthcare system, some patients might have funded their operation privately, resulting in selection bias. In a recent analysis about 40% of bariatric surgery operations in the UK were privately funded. Thus, the intervention group might have a higher socioeconomic status than the non-exposed group, in which similar patients would not be able to afford surgery. Since the socioeconomic background is an important determinant of health outcomes and was an unmeasured potential confounder not considered in the matching process, this could have led to more positive health outcomes in the intervention group irrespective of surgery and to an overestimation of the effect. In this study setting it was not possible to determine which patients had privately funded surgery.<br>Even though most baseline variables were evenly distributed due to the matching process this does not guarantee that unmeasured variables are evenly distributed as well, which can constitute residual confounding. Incorrect, imprecise, or missing measurements of covariates could also have led to residual confounding. For the multivariable model, adjusting for history of AKI led to the strongest change of the effect estimate. AKI events are likely under-recorded in the HES database, for reasons described above, and thus residual confounding is possible. Since adjusting for AKI history led to a stronger effect estimate, the protective effect we report here may be an underestimate if AKI history is missing to the same degree in surgery and non-surgery patients.“
Interpretation	20	Give a cautious overall interpretation of results considering objectives, limitations, multiplicity of analyses, results from similar studies, and other relevant evidence<br><br>Page 18: “This study adds to the evidence of long term effects of bariatric surgery, and appears to be the first study to quantify a long-term beneficial effect on AKI. Future studies with higher patient numbers may be able to determine differences in effect between types of surgery, investigate the effect in patients with CKD, and elucidate mechanisms of the association between bariatric surgery and AKI.”
Generalisability	21	Discuss the generalisability (external validity) of the study results<br><br>Page 15/16: “Even though the data is taken from a representative sample of the UK population, the baseline data indicate that patients who undergo bariatric surgery are mostly female, of middle age, and with a history of T2DM. While the results were adjusted for age and sex they might not be applicable for other groups suffering from obesity like adolescents. Linkage between the CPRD and HES databases was restricted to England. However, there is no cogent reason why the results should not

For peer review only

		be applicable to regions with similar healthcare systems, both in the UK and internationally."
**Other information**		
Funding	22	Give the source of funding and the role of the funders for the present study and, if applicable, for the original study on which the present article is based<br><br>RM is supported by a Sir Henry Wellcome Postdoctoral Fellowship from the Wellcome Trust. KB holds a Sir Henry Dale fellowship jointly funded by the Wellcome Trust and the Royal Society. RLB is an NIHR Research Professor and supported by funding from the Rosetrees Trust and the Sir Jules Thorn Charitable Trust. LS is supported by a senior clinical fellowship from the Wellcome Trust. IJD is funded by an unrestricted grant from GlaxoSmithKline.

*Give information separately for exposed and unexposed groups.

Note: An Explanation and Elaboration article discusses each checklist item and gives methodological background and published examples of transparent reporting. The STROBE checklist is best used in conjunction with this article (freely available on the Web sites of PLoS Medicine at http://www.plosmedicine.org/, Annals of Internal Medicine at http://www.annals.org/, and Epidemiology at http://www.epidem.com/). Information on the STROBE Initiative is available at http://www.strobe-statement.org.

For peer review only
